# Robust but independent sex differences in human brain function, structure, and behavior

**DOI:** 10.1038/s41467-026-73262-2

**Published:** 2026-05-21

**Authors:** Siyuan Liu, Bridget W. Mahony, Ethan T. Whitman, Stephen J. Gotts, Dustin Moraczewski, Adam Thomas, Alex Martin, Armin Raznahan

**Affiliations:** 1 Section on Developmental Neurogenomics, Human Genetics Branch, National Institute of Mental Health, Bethesda, MD USA; 2 Section on Cognitive Neuropsychology, Laboratory of Brain and Cognition, National Institute of Mental Health, Bethesda, MD USA; 3 Data Science and Sharing Team, National Institute of Mental Health, Bethesda, MD USA

**Keywords:** Neuroscience, Psychology

## Abstract

The neurobiological accompaniments of well-established sex differences in human behavior and disease remain unclear — in part due to a lack of large, diverse functional neuroimaging studies. We address this gap using over 700 h of fMRI data across seven tasks from 978 individuals with extensive structural and behavioral measures. We find that sex differences in task-activation are widespread (85% of cortex) and reproducible,  largely task-specific, of small to moderate effect size, and unaligned with brain volume differences. While machine learning can classify sex from brain activation, volume, or behavior, these data types provide orthogonal information. Brain-wide association studies reveal that links between brain activation and behavior are highly conserved between sexes. The few subtle sex differences in brain-behavior linkage that do exist are not preferentially localized to sex-biased behaviors. Our findings clarify the nature of sex differences in human brain function and their links with neuroanatomy and behavior, providing a useful foundation for future research.

## Abstract (web summary)

Although robust sex differences exist in human brain function, anatomy, and behavior, these traits are largely independent. A sex-typical brain function does not necessarily translate to sex-typical brain structure or behavior.

## Introduction

Human males and females show differences in the prevalence, presentation, and course of diverse neuropsychiatric conditions (e.g., autism and mood disorders)^1–7^, as well as in specific aspects of behavior (e.g., physical aggression)^8–11^ and cognition (e.g., mental rotation, face perception, and language)^12–15^. These sex differences likely reflect complex influences of diverse biological, psychological, and social factors on human brain function and its behavioral manifestations^16–19^. However, despite major research investments in neuroimaging and behavioral research, there remains intense debate and controversy regarding the very existence of sex differences in human brain function and whether any such differences relate to sex differences in behavior (SDBs).

Currently, the largest available corpus of functional measures from the living human brain comes from functional magnetic resonance imaging (fMRI), which can resolve regional brain activation while participants are completing tasks designed to tap different aspects of behavior and cognition^20,21^. However, in application to the study of sex differences, task-based fMRI studies have so far largely consisted of individual experiments that are each focused on just one of many conceivable tasks (e.g., isolated studies of verbal^22,23^ and visuospatial processing^24,25^, emotional recognition^26–28^, gambling^29,30^, and social interaction^31,32^) in participant cohorts that are often too small to allow tests of reproducibility. Moreover, the small sample sizes and methodological heterogeneity of these published studies frustrate attempts at meta-analytic integration^4,33,34^. As such, the magnitude, reproducibility, and task-dependency of sex-biased brain activation in humans remain unclear. It is even less clear if any observed sex differences in brain activation are related to sex differences in behavior   because addressing this question hinges on the rare combination of multi-task fMRI data in large cohorts with high-dimensional behavioral data.

Here, we address these fundamental gaps in knowledge by leveraging a globally unique dataset that combines over 700 h of fMRI data across seven different tasks spanning diverse domains of brain function, with accompanying measures of brain anatomy and over 85 different behavioral scales in close to 1000 individuals (Human Connectome Project, HCP)^35^. We harness this remarkably rich multimodal dataset to comprehensively map the magnitude, reproducibility, and spatial distribution of sex differences in human brain activation (SDAs) and to also determine the extent to which any such differences are task-specific (task-specific SDAs) vs consistently present regardless of task content (task-general SDAs). Having systematically profiled SDAs, we then apply a series of complementary analyses to contextualize SDAs relative to co-occurring sex-differences in regional human brain volume (SDVs) and then address the crucial question of whether SDAs are related to sex differences in human behavior (SDBs).

We find that variations in human brain activation, brain volume, and behavior reproducibly differ in their mean values between males and females and can be used to accurately predict sex. However, the degree of a person’s “sex typicality” in one domain of measurement is largely uncoupled from that in another.  Moreover, although reliable associations do exist between variation in brain activation and behavior within each sex, these associations are largely indistinguishable between sexes. This comprehensive multiscale analysis provides rich empirical data to inform longstanding debates regarding the nature and behavioral relevance of sex differences in human brain function—helping to shape our current understanding and future queries of sex as a neurobiological variable.

## Results

### Humans show both task-specific and task-general sex differences in brain activation

We used fMRI data from the HCP S1200 data release (March 2017)^35^ to quantify sex differences in brain activation (SDAs) in 978 healthy young adults (455 males and 523 females, aged 22–37 years) who each completed seven different fMRI tasks: emotion processing, gambling, relational processing, social cognition, language, working memory and motor (Supplementary Fig. 1). These fMRI data were processed using gold-standard methods (HCP V3 preprocessing and Multimodal Surface Matching All pipelines^36,37^, “Methods”) to generate subject-level activation maps for each task across 360 cortical regions (HCP Multi-modal Cortical Parcellation: 180 regions per hemisphere^37^, characterized in Supplementary Data 1, “Methods”). Group-level activation maps for each task (“Methods”, Supplementary Fig. 1 and Data 1) verified the diverse patterns of regional activations and deactivations that have been previously reported for each task^38–43^—setting the stage to test for SDAs across multiple cognitive, affective, and sensorimotor domains. To achieve this test, we used a linear mixed effect model to estimate activation at each cortical region as a function of task, sex, and their interaction (controlling for the fixed effect of age and nested random effect term for person|family, “Methods”). This approach quantifies both task-specific SDAs (the interaction between sex and task) and task-general SDAs (the main effect of sex).

**Fig. 1:**
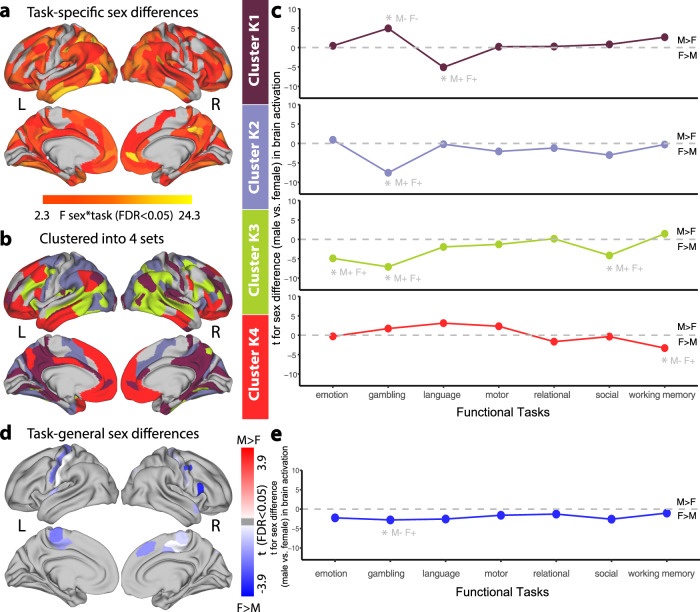
Humans show task-specific and task-general sex differences in brain activation. **a** Surface projections showing statistically significant (FDR < 0.05) task-specific sex differences in activation (SDAs,*F* values of sex × task in a mixed-effect model 1) on the HCP parcellation of 360 cortical regions. **b** Clustering of regions in (**a**) by k-means based on their profiles of effect sizes for sex-biased activation across all 7 tasks. **c** Each cluster exhibited a distinct line-graph profile of SDAs across the seven tasks, reflected by task-dependent variation in *t* values comparing males and females that differed between clusters; ^*^Denotes statistical significance of two-sided sex differences in the given task, evaluated by age-adjusted regression models (d*f* = 975) after Bonferroni correction across tasks and clusters (*P* < 0.05/7/4) and exact *P* values are provided in Supplementary Data 4; plus/minus sign marks activation/deactivation per task in males [M] and females [F]). **d** Regions with statistically significant task-general SDAs after FDR correction across brain regions (FDR *q* < 0.05). **e** Profiles of SDAs for regions exhibiting task-general SDAs across all seven tasks, represented by line graphs, showing consistently negative *t* values (i.e., female-biased activation) for the male–female contrast, indicating sex differences that are independent of task variation.^*^Denotes statistical significance of two-sided sex differences in the given task, evaluated by age-adjusted regression models (d*f* = 975) after Bonferroni correction across tasks (*P* < 0.05/7) and exact *P* values are provided in Supplementary Data 4; plus/minus sign marks activation/deactivation per task in males [M] and females [F]. Source data are provided as Source Data files.

We identified statistically significant task-specific SDAs in >80% of all cortical regions (296 out of 360 HCP parcellations surviving False Discovery Rate (FDR) correction for multiple comparisons across regions at q < 0.05, “Methods”, Fig. 1a and Supplementary Data 2). These significant task-specific SDAs reflect spatial dissimilarities between the maps of SDAs for individual tasks (“Methods”, Supplementary Fig. 2 and Supplementary Data 3)—which exist above and beyond small and isolated sex differences in task performance (Supplementary Fig. 3). Cortical regions showing statistically significant task-specific SDAs (Fig. 1a) could be clustered into 4 groups based on their SDA profiles across all seven tasks (K-means clustering, “Methods”, Fig. 1b, c, Supplementary Fig. 4a–c, and Supplementary Data 2). Each of these 4 regionally distributed clusters possessed a unique profile of task-specific SDAs across tasks (statistically significant cluster-level task-specific SDAs denoted with asterisks in Fig. 1c and Supplementary Fig. 4d, Supplementary Data 4, sex-specific profiles in Supplementary Fig. 4e). For example, cluster K1—which encompassed posterior cingulate, retrosplenial, parieto-occipital, dorsolateral prefrontal, and frontal opercular cortices—showed greater deactivation in females than males during gambling and greater activation in females than males during the language task. In contrast, cluster K3—which encompassed superior temporal and temporo-parieto-occipital, auditory, perirhinal, middle frontal, and inferior parietal cortices – showed greater activation in females than males during gambling, emotion, and social tasks. Testing for task-general SDAs beyond the K1-K4 regions of task-specific SDAs, revealed statistically significant task-general SDAs (FDR q < 0.05) within bilateral motor and somatosensory cortices (14 out of 64 HCP parcellations that did not show task-specific SDAs, Fig. 1d and Supplementary Data 5), which showed a generic tendency across all seven tasks of a greater activity magnitude in females than in males (a difference which reached statistical significance during the gambling task, Fig. 1e and Supplementary Data 5, sex-specific profiles in Supplementary Fig. 4f). Effect sizes for statistically significant regional, task-specific and task-general SDAs were generally small to moderate. At the cluster level within individual tasks, effect sizes fell in the small-to-moderate range (|Cohen’s *d*|: median = 0.3, range = 0.2–0.5; Supplementary Data 4), whereas at the region level across tasks, effect sizes were small (partial eta-squared: median = 0.005, range = 0.0021–0.022; Supplementary Fig. 5a, b; Data 2, 5). Despite these small to moderate effect sizes, spatial patterns showed moderate to strong reproducibility across regions, as demonstrated by 1000 independent split-half analyses of the HCP dataset (spatial *r* = 0.6 for task-specific SDAs and 0.8 for task-general SDAs, respectively, “Methods”, Supplementary Fig. 5c, d). Thus, large swaths of the human cortex show reproducible SDAs of small to moderate effect size, which are organized into spatially distinct subsets with unique profiles of SDAs across different tasks.

To contextualize SDAs against regional functional neuroanatomy, using Spin Tests^44,45^, we assessed their spatial correspondence with meta-analytic brain activation maps (Neurosynth^46–48^) and canonical resting-state connectivity networks (Yeo-Krienen 17 network parcellation^49^, Supplementary Text 1,Supplementary Fig. 6). Both task-specific and task-general SDAs were enriched within distributed systems supporting motor, attentional, language, and social processes, suggesting differential engagement of functional networks by males and females during task performance. Importantly, however, this spatial alignment alone does not speak to whether SDAs are relevant for domains of behavior associated with each highlighted brain system.

### Sex differences in brain activation and brain volume are not spatially correlated

Sex differences in fMRI measures of brain activation could potentially be related to well-described sex differences in brain anatomy^4,50–54^ for both biological and methodological^55^ reasons. We therefore next tested if the spatial patterning of those reproducible cortical SDAs we defined by fMRI (Fig. 1) is related to similarly reproducible sex differences in regional cortical gray matter volume (GMV), which have been described by structural MRI and exist above and beyond sex differences in overall brain volume^4,50–54^.

**Fig. 2:**
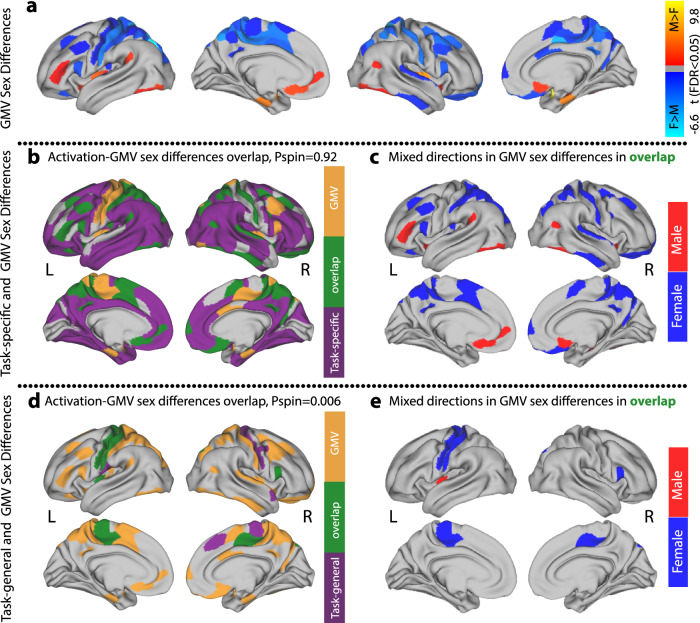
Limited spatial convergence of sex differences in brain activation and brain volume. **a** Statistically significant sex differences in brain volume (SDVs) surviving correction for multiple comparisons across brain regions (FDR *q* < 0.05) after co-variation for total GMV. **b** Conjunction between regions of statistically significant task-specific SDAs and SDVs (Supplementary Data 6). The observed degree of overlap between these region sets was not elevated beyond a null distribution based on Spin Tests with 10k permutations (*P*_spin_ = 0.92). **c** The direction of SDV in those regions showing statistical significance for both task-specific SDAs and SDVs (green in **b**). **d** Conjunction between regions of statistically significant task-general SDAs and SDVs (Supplementary Data 6). The observed degree of overlap between these region sets was greater than a distribution based on Spin Tests with 10k permutations (*P*_spin_ = 0.006). **e** The direction of SDVs in those regions showing statistical significance for both task-general SDAs and SDVs (green in **d**). GMV gray matter volume.

Structural MRI scans for the same individuals used to estimate SDA were submitted to FreeSurfer for surface-based estimation of regional cortical volume^56–58^ (“Methods”), and regression models were used to estimate regional volume as a function of sex, controlling for age and total GMV (“Methods”). We confirmed the existence of statistically significant (FDR < 0.05) sex differences in regional cortical volume (SDV, “Methods”, Fig. 2a) that range in absolute effect size from 0.2 to 0.6 (Supplementary Fig. 7). These SDVs have been previously described in the HCP dataset^50^ and closely resemble those reported in independent datasets^53^.

Given that both task-specific SDAs and SDVs were evident in large swaths of the cortex, we observed several cortical regions that display statistically significant sex differences in both activation and volume (green regions, Fig. 2b and Supplementary Data 6), but there was no consistent alignment in the direction of sex differences for fMRI and structural MRI data within these overlap regions with a mixture of both male- and female-biases in volume (Fig. 2c) and task-specific activation (Supplementary Fig. 8). Moreover, the observed degree of spatial overlap between regions of statistically significant task-specific SDAs and SDVs did not exceed that observed across 10k random spatial permutations of these maps (*P*_spin_ = 0.9, “Methods”)—indicating a lack of significant spatial enrichment of regional task-specific SDAs within regional SDVs. Supplementary analyses also established a lack of spatial correlation between the unthresholded absolute amplitudes of task-specific SDAs and SDVs (*r* = −0.06, *P*_spin_ = 0.4, Supplementary Fig. 8).

In contrast to the lack of spatial alignment between task-specific SDAs and SDVs, we did observe a significant spatial overlap between task-general SDAs and SDVs (*P*_spin_ = 0.006)—defining bilateral supplementary motor, left posterior insular and right Broca’s area (green in Fig. 2d and Supplementary Data 6) as cortical regions that show a tendency towards female bias in both their volume and cross-task activation (Fig. 2d, e and Supplementary Fig. 9a). Of note, however, there was no correlation between interindividual variation in task-general activation and volume within these overlap regions (*r* = 0.05, Supplementary Fig. 9b).

Taken together, these findings reveal a general lack of spatial correspondence between SDAs and SDVs within the human brain—with the exception of overlapping female-biased activation and volume in motor cortices (albeit with a lack of interindividual correlation between volume and activation). These observations suggest that sex differences in activation and volume may capture largely distinct facets of sex-biased brain organization.

### Multivariate patterns of brain activation, brain anatomy, and behavior are all highly but orthogonally predictive of participant sex

**Fig. 3:**
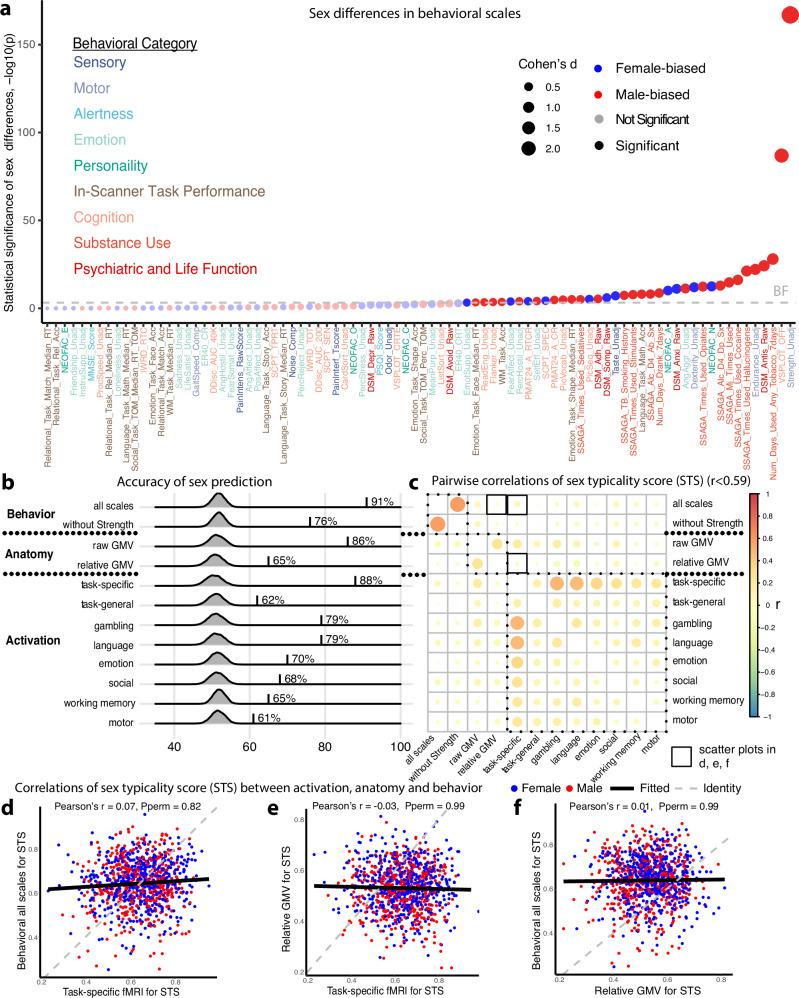
Patterns of brain activation, anatomy, and behavior can predict sex, but individual scores for sex typicality in these features are uncorrelated. **a** A ranked circle plot showing sex differences in 86 raw, unadjusted, numerical, behavioral scales (Supplementary Data 7). Scales are ranked on the *x*-axis by decreasing statistical significance of the two-sided sex difference [−log10(*P*) value on *y*-axis]. Significant scales, marked by non-transparent dots, survive Bonferroni correction for 86 scales (i.e., above the dashed gray line) and vice versa. Dot size and color encode the Cohen’s *d* effect size and direction (red: male > female; blue: female > male) of the sex difference, respectively. **b** The accuracy of sex prediction by partial least squares discriminant analysis (PLSDA) with 10-fold cross-validation for 12 different feature sets derived from behavioral, structural MRI, and fMRI data (“Methods”). The achieved accuracy of sex prediction for each feature set (bar) is shown relative to a null distribution of accuracies from 10k permutations of sex. **c** Dot plot showing correlations between person-level sex typicality scores (STSs) for each of the 12 feature sets used in PLSDA. Each person’s STS of a feature set is the probability assigned to that person for membership of their true sex group based on PLSDA of that feature set. Dot size and color encode correlations. The three cells with black borders denote correlations for which scatterplots are provided in (**d**–**f**). **d**–**f** Scatter plots for pairwise correlations between selected STS scores: STS from task-specific activation vs STS from all behavioral scales (**d**); STS from task-specific activation vs STS from anatomy (relative regional GMV) (**e**); STS from anatomy (relative regional GMV) vs STS from all behavioral scales (**f**). Source data are provided as Source Data files.

The above findings establish that the human brain shows reproducible and regionally specific sex differences in activation and volume, but that these differences are in large part not spatially coordinated across the cortical sheet—suggesting they may reflect dissociable phenomena. To further probe this provocative idea, we next turned to the domain of interindividual variation—asking if individuals with more sex-typical profiles in one trait are also more sex-typical in another. This approach enabled us to also incorporate sex differences in behavior (SDBs) by harnessing available measures of 86 different behavioral traits from 9 categories (Fig. 3a and Supplementary Data 7, “Methods”) in the same individuals used for neuroimaging analyses of SDAs and SDVs. Figure 3a shows these 86 traits measured ranked by the magnitude of SDBs as estimated using linear regression models for continuous scales and generalized linear models with a Poisson distribution for counts (age was controlled as a covariate in both, “Methods”). Over 40% of these traits showed statistically significant SDBs (Bonferroni correction, *P* < 0.05/86) with absolute effects sizes (Cohen’s *d*) ranging from 0.2 −2.2 and differences being most prominent for grip strength^59^, visual judgment of line orientation^60,61^, and smoking frequency (all male-biased), as well as tendency to experience psychological distress^62^ and anxiety^63^, and possess greater dexterity in the dominant hand^59^ (all female-biased).

We used a machine learning framework^64^ to score each person in the HCP dataset for the “sex-typicality” of their individual profiles of brain activity, brain volume, and behavior. Specifically, for each data modality (activation, anatomy, and behavior), we trained a partial least squares discriminant analysis (PLSDA) algorithm with 10-fold cross-validation to predict an individual’s sex (Methods). This approach yielded accuracy scores for each modality in the prediction of sex, as well as modality-specific person-level sex typicality scores (STSs)—defined as the probability assigned to an individual for their true sex group based on their multivariate patterns of brain activation, brain volume, or behavior. The input data for these analyses were individual-level measures of brain activation and volume in regions showing statistically significant SDAs and SDVs, respectively (Figs. 1a, d and 2a), and individual-level measures of behavior for those scales showing statistically significant SDBs (Fig. 3a).

Individual profiles of brain activity, brain anatomy, and behavior all showed a capacity for high accuracy in correctly predicting an individual’s sex (Fig. 3b). For brain activation, sex was best-predicted by individual profiles of task-specific activation (88% accuracy), while task-general activation was more weakly predictive of sex (62% accuracy). Amongst individual tasks, activation profiles were most strongly predictive of sex during gambling and language tasks (both 79% accuracy) and least so during the motor task (61% accuracy). Sex could also be accurately predicted by individual profiles of regional brain volume (raw GMV: 86% accuracy), although this deteriorated when total GMV was regressed out of regional measures (relative GMV: 65% accuracy). Individual profiles of behavior showed the highest accuracy of all traits for sex prediction (91%) and remained high (76% accuracy) when the single measure of grip strength (with an outlier effect size for sex) was excluded.

Strikingly, despite the capacity of brain activation, brain anatomy, and behavior to each correctly predict an individual’s sex, we observed universally weak correlations between person-level STSs in these three phenotypic domains (Fig. 3c)—as detailed in Fig. 3d–f for person-level STSs from task-specific brain activations, relative regional brain volume, and behavior. Pairwise correlations between these three STSs were weak (all |*r*| < 0.07) and not statistically significant compared to an empirical null distribution based on sex permutation (all *P*_perm_ > 0.8, Fig. 3d–f and Supplementary Data 8, “Methods”). These weak interrelationships between multivariate measures were also observed when we considered non-volumetric measures of regional brain anatomy, particularly, surface area and cortical thickness (Supplementary Fig. 10). In line with these findings, person-level STSs exhibit substantial variability across all 16 measures, with only a small fraction of individuals (<7% of females and <2% of males) achieving accurate sex prediction across all models (Supplementary Fig. 11).

It is important to note that these sex-prediction accuracy and associated STSs are based on 10-fold cross-validation, where the definition of sex-biased brain regions and behavioral traits for input to the Partial Least Squares Discriminant Algorithm was conducted in the same HCP dataset, where held-out individuals were used to test model performance. Because the HCP dataset is unique in its large battery of fMRI tasks and behavioral traits, we were only able to directly test for evidence of potential data leakage in analyses based on structural MRI data, which are extensively available in large independent datasets such as the UK Biobank^65^. We therefore defined maps of sex-biased cortical GMV, surface area, and cortical thickness in this independent dataset (“Methods”)—which were highly similar to those in the HCP dataset (spatial correlations > 0.63; Supplementary Fig. 12a, b)—and used these to define input data for Partial Least Squares Discriminant Algorithm classification of sex in the HCP dataset. These supplementary analyses found almost identical sex prediction accuracy and STSs as our primary analyses in the HCP (Supplementary Fig. 12c), arguing for the good generalizability of our findings for structural MRI across datasets.

Thus, although (i) the human brain shows highly reproducible regional SDAs (Fig. 1) accompanied by SDVs (Fig. 2a) and SDBs (Fig. 3a), and (ii) individual profiles of brain activation, brain anatomy and behavior are each capable of correctly classifying an individual’s sex (Fig. 3b), (iii) these profiles appear to be largely orthogonal to each other such that an individual’s sex typicality in one is not predictive of that in another.

### Brain-wide association studies link behavior and task-induced brain activation within each sex

The lack of any correlation between an individual’s overall sex-typicality in task-induced brain activation and behavior (Fig. 3d) does not speak to the possibility of interactive effects—where sex modulates the association between interindividual variation in brain activation and behavior. We therefore next sought to systematically test for such interactions at fine-grain, considering all possible combinations of behavior, task, and brain region. This required estimating cross-individual associations between variation in behavior and variation in task-specific regional brain activation—a procedure also known as a Brain Wide Association Study (BWAS). However, prior BWAS reports have considered brain activation at rest^66^, and there is evidence that the task-dependent brain activations we study herein may show boosted associations with behavior^67^. We therefore first quantified BWAS signals for task-dependent brain activation within each sex before seeking to compare BWAS signals between sexes.

**Fig. 4:**
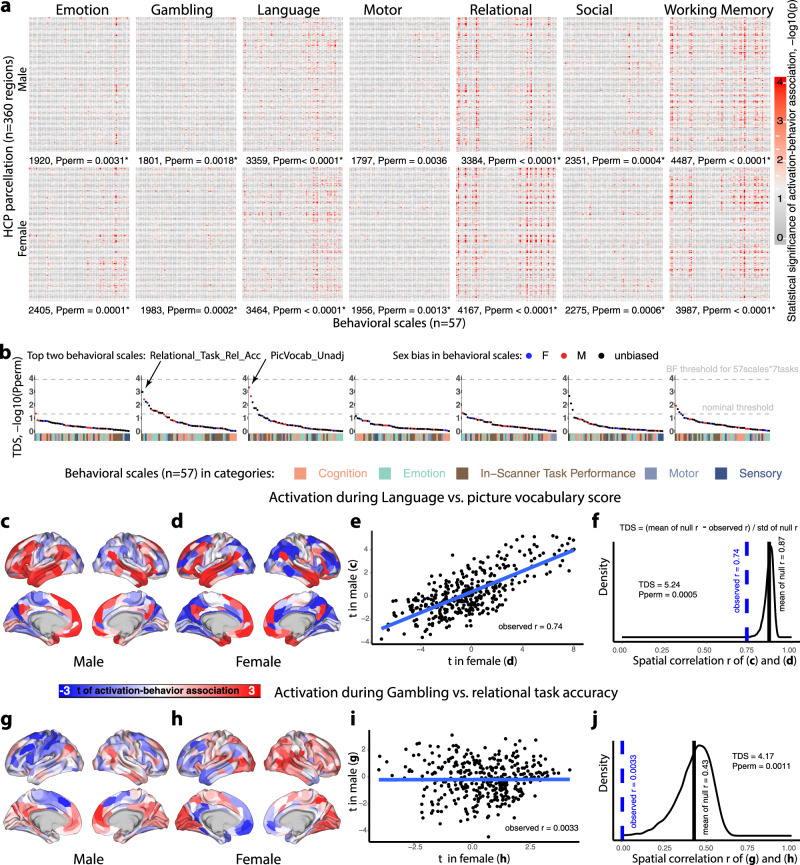
Males and females show largely similar brain-wide associations between task activation and behavior. **a** Matrices showing statistical significance [−log10(P)] of brain-wide association studies (BWAS) in males (top row of matrices) and females (bottom row) for each of seven fMRI tasks (columns). Each matrix shows pairwise associations between interindividual variation in task-induced activation and interindividual variation in behavior for all unique pairs of 360 cortical regions (matrix rows) and 57 continuous behavioral scales (matrix columns). *P*_perm_ values at the bottom of each matrix show results of one-sided permutation-based tests for whether the observed count of matrix cells with nominally significant *P* values is statistically larger (Bonferroni corrected, *P*_perm_ < 0.05/14) relative to the distribution of counts from 10k permutations of behavior scores across participants within each sex group. **b** Dot plots rank the 57 behaviors (shown in *x*-axis with colors matching Fig. 3a to denote behavioral category) by descending topographical divergence scores (TDSs) between sexes for each task (greater TDS indicting greater topographic divergence between sexes in brain-wide association study maps, *P* values are determined by a separate one-sided 10k sex-shuffling permutation test for spatial correlation of BWAS maps in males and females, “Methods”). Dot color indicates whether the mean behavioral score is female-biased (blue), male-biased (red), or neither (black). No behaviors showed TDS scores that were significantly elevated above zero after Bonferroni correction for multiple comparisons across all 57 scales and 7 tasks (*P*_perm_ < 0.05/399), although 29 out of 399 behavior-task combinations (Supplementary Data 10) showed nominally significant between-sex TDS elevation (*P*_perm_ < 0.05), with the greatest TDS score being seen for associations between a behavioral measure of picture vocabulary performance and brain activation during the language task fMRI, and the second greatest for a measure of in-scanner performance accuracy in a relation task and brain activation during the gambling task fMRI. **c**–**j** *Post hoc* analyses of elevated between-sex TDS scores for BWAS maps in these top two combinations. **c**–**f** For the first combination, the BWAS maps in males (**c**) and females (**d**) for the association between regional brain activation during the language task fMRI and picture vocabulary performance. **e** Scatter plot showing a high spatial correlation between the BWAS maps in (**c**, **d**). However, **f** density plot showing TDS (reflecting the distance between this observed correlation in blue vs the mean of 10k sex-shuffling null correlations in black as illustrated in the formula here and “Methods”) remains elevated because the observed correlation is significantly lower than the null mean. **g**–**j** For the second combination, the BWAS maps in males (**g**) and females (**h**) for association between regional brain activation during the gambling task fMRI and in-scanner performance accuracy in a relation task during the relational task fMRI. **i** Scatter plot showing the lack of any spatial correlation between the BWAS maps in males (**g**) and females (**h**)—indicating that the topography of activation-behavior associations is highly dissimilar between males and females for this specific behavioral scale and fMRI task paradigm combination. **j** Density plot confirming large TDS due to the low observed spatial correlation relative to the null mean. Again, in (**f**, **j**), *P*_perm_ values are determined by a one-sided 10k sex-shuffling permutation as described in (**b**). Source data are provided as Source Data files.

We tested for detectable BWAS signals in each sex using a permutation that randomized behavioral scores on each scale across individuals within each sex group. Specifically, for each of the seven fMRI tasks assessed and separately within each sex, we first estimated the full matrix of 360 × 57 pairwise correlations between regional brain activation (360 cortical regions) and scores on continuous behavioral scales (*n* = 57, Supplementary Data 7). Then, in each of the 14 matrices generated (7 tasks in each of 2 sexes), we counted the number of associations that were statistically significant at nominal *P* < 0.05, and compared this count to those from 10k random permutations of behavioral scores within sex to derive an empirical *P*_perm_ value which was then Bonferroni-corrected for multiple comparisons across the 14 matrices (*P*_perm_ < 0.05/14). These procedures revealed a statistically significant global BWAS signal for activations from each of the 7 fMRI tasks in each sex group—with the sole exception of the motor task in males (Fig. 4a). The distribution of significant BWAS associations across all possible behavior-region combinations showed strong reproducibility based on 1000 independent split-half analyses within each sex for each task (*r* values all >0.7, “Methods”, Supplementary Fig. 13).

We noted a striking columnal organization to activation-behavior associations in each task (Fig. 4a)—indicating that for any given task—a subset of behaviors shows association with activations across multiple regions rather than a subset of regions showing activation associations across multiple behaviors. We therefore conducted column-wise *post hoc* analyses that compared the observed counts of nominal activation-behavior associations for every column (i.e., behavioral scale) of each 360 × 57 matrix with corresponding counts from the 10k permutations above to specify those behaviors with enriched BWAS signal for each of the 7 HCP fMRI tasks in each sex (“Methods”). A total of 19 behavioral scales exhibited significant activation-behavior associations in at least one sex in at least one task (Supplementary Fig. 14 and Data 9 for the complete per-scale results in each sex). Many of these behavioral scales are related to working memory, and higher cognitive and social functions like delayed discounting (a measure of self-regulation and neuroeconomic decision making) and language. Strikingly, however, in several instances, the significant BWAS signals seen for an fMRI task involved behavioral measures that were ostensibly unrelated to that fMRI task paradigm (e.g., measures of endurance and delay discounting were significantly associated with brain activation during a language task, Supplementary Fig. 14 and Data 9).

Taken together, these analyses suggest that significant BWAS signals can indeed be detected between behavior and task-induced brain activation in humans (Fig. 4a). For a given fMRI task, certain behaviors tend to show BWAS signals across multiple regions (including some behaviors involving domains not obviously tapped by the fMRI task). These findings set the stage for comparing BWAS signals between sexes.

### Males and females show broadly similar brain-wide association study signals with a few isolated exceptions

Having established the presence of significant BWAS signals between task-induced brain activation and behavior within each sex, we turned to our core test for sex-based interactions: comparing these activation-behavior associations between males and females. We compared these associations between sexes at two complementary levels of spatial resolution for each fMRI task—the overall topography of BWAS signals across the cortical sheet, and the nature of activation-behavior associations at individual brain regions.

First, within each of the seven task fMRI paradigms, we quantified how dissimilar males and females were in their BWAS maps for each of the 57 behavioral scales and compared the observed dissimilarity scores to those from a null distribution based on 10k permutations of sex (“Methods”). Spatial dissimilarity in BWAS maps between sexes was computed as a topographic divergence score (TDS), which is a standardized *Z*-score where higher values reflect greater spatial dissimilarity between males and females than between mixed sex groups (“Methods”). We screened for evidence of significantly elevated TDS scores across all behavior-task combinations using both a strict and conservative Bonferroni correction across 57 × 7 combinations, and a nominal *P* < 0.05 threshold. No single behavioral scale showed a significantly elevated TDS surviving strict Bonferroni correction (*P*_perm_ < 0.05/399, Fig. 4b, complete values in Supplementary Data 10). A total of 29 behavior-task combinations (7% of all possible behavior-task combinations) showed elevated TDS between sexes at nominal statistical significance. Notably, for each of the seven tasks where these nominally significant TDS elevations were seen, the behavior-task combinations involved were not enriched for behavioral scales showing a statistically significant SDB (*P* > 0.2 in Fisher’s Exact tests for associations between whether a scale shows statistically significant SDB, Fig. 3a, and TDS, Fig. 4b, across seven tasks)—indicating that observing a difference in the mean score of a behavioral trait between sexes is not predictive of that trait showing a divergent BWAS map between sexes. We provide illustrative details for the two behavior-task combinations with the most significantly (albeit nominally) elevated TDSs in Fig. 4c–j). The most prominent TDS elevation in our dataset was seen for the BWAS of picture vocabulary performance (a significantly male-biased behavior, Fig. 3a) during the language fMRI task (Fig. 4b): the BWAS topography was positively correlated between males and females (*r* = 0.74), but this observed correlation was still significantly below that observed between 10k sex-shuffled groups (*r* = 0.87). The second most prominent TDS elevation was seen for the BWAS of relational reasoning accuracy (a sex-unbiased behavior, Fig. 3a) during the gambling fMRI task: the BWAS topography for the behavior-task combination was totally uncorrelated between males and females (*r* = 0.003, Fig. 4g–j). Taken together, these analyses indicate that the overall topography of activity-behavior associations is broadly similar between males and females for most of the fMRI tasks and behavior combinations assessed — although there are isolated instances where the topography of these associations can be highly divergent between sexes.

Second—for a more spatially fine-grained analysis—we directly quantified the interaction between sex and brain activation in predicting behavior for all possible task-region combinations and asked if the observed magnitude of such interactions exceeded null expectations. Specifically, for each of the seven task fMRI paradigms assessed, we computed the full 360 × 57 matrix of such interactions (at each of 360 cortical regions for each of 57 behaviors, Supplementary Fig. 15a) and compared the observed count of interactions surviving a nominal *P* < 0.05 threshold to a distribution of counts from 10k permutations of sex labels across participants (“Methods”). None of the seven fMRI tasks examined shows a statistically surprising omnibus elevation in sex modulation of regional activation-behavior associations surviving correction for multiple comparisons across tasks (*P*_perm_ < 0.05/7, Supplementary Fig. 15b). Moreover: (i) a broad screen failed to find any behavior-task combinations with a significant elevation in sex modulation of activation-behavior associations that survived correction across all unique behavior-task combinations (*P*_perm_ < 0.05/399, Supplementary Fig. 15c); and (ii) those few behavior-task combinations that showed nominal evidence for sex modulation of activation-behavior associations without any correction for multiple comparisons (10% of all behavior-task combinations, Supplementary Data 11) were again not enriched for behavioral scales with evidence for a statistically significant SDB (Fig. 3a, *P* > 0.2).

Collectively, these exhaustive BWAS analyses establish that associations between task-dependent brain activation and behavior are present in humans, but broadly similar between males and females. Those few subtle sex differences that can be observed: are narrowly isolated to specific behavior-task combinations, do not reach statistical significance in the context of a large analysis of diverse fMRI tasks and behaviors, and do not preferentially involve sex-biased behavioral scales.

## Discussion

By integrating regional measures of brain activation across multiple tasks with complementary measures of regional brain anatomy and behavior, our study usefully advances understanding of sex differences in human brain organization on four key fronts as detailed below.

First, we establish that humans show reproducible and spatially extensive sex differences in mean regional brain activation as estimated by fMRI across a diverse set of seven different tasks. The vast majority of these sex differences in regional brain activation are task-specific, with the exception of those in bilateral sensorimotor cortices, which show a generic female-bias in activation across all assayed tasks. Task-specific sex differences were evident across multiple clusters of brain regions that exhibited sex differences in distinct individual tasks, including gambling, language, and social cognition, with gambling emerging across several clusters (potentially reflecting the robust capacity of this task to strongly modulate activity across the brain, Supplementary Fig. 1). These findings echo prior reports of sex differences observed in isolated task-based fMRI paradigms of gambling^29,30^, verbal processing^22,23^, and social interaction^31,32^, as well as in behavioral studies of gambling-related decision-making and impulsivity^9,10^. Together with the small effect sizes observed here, this heterogeneity in task-specific sex differences in brain function may help explain the inconsistent findings reported in previous meta-analyses^4,33,34^ of largely small-sample studies. The fact that those sex differences in activation  which we report herein are enriched in several canonical functional brain systems implies that males and females subtly differ in their relative engagement of different functional brain systems to achieve the same task (notwithstanding small and isolated sex differences for some measures of task performance). This interpretation aligns with orthogonal evidence from prior lesion mapping studies for sex differences in the spatial patterning of outcome prediction after stroke^68^. If sex is indeed associated with use of different neural strategies to support the same behavior, then this could set up sex-specific pathways to behavioral risk and resilience in the absence of sex differences in mean behavioral performance—further reinforcing the value of sex as a potential stratifier for advancement of precision medicine^69–72^.

Second, we show that the spatial patterning of sex differences in brain activation  is not organized by co-occurring sex differences in brain volume—although the regionally distributed nature of these features defines a subset of brain regions that are sex-biased in both their structure and function and may therefore represent high-priority targets for future research. Nevertheless, the overall lack of spatial coupling between regional sex-biased brain activation and volume indicates that sex differences in activation are unlikely to arise as a methodological artifact of sex differences in brain volume^55,73^ and must also reflect partly dissociable biological processes. Interestingly, our findings for task-based measures of brain activation are consistent with recent studies of sex-differences in resting state brain activation, which were also found to be independent of sex differences in cortical morphometry^74^. Nevertheless, future studies that directly compare sex differences in task-evoked and resting-state functional organization alongside brain anatomy will be valuable for determining whether sex differences in resting-state function show greater spatial congruence with anatomical sex differences than task-evoked activation.

Third, by also mapping sex differences in behavior and considering these alongside measures of brain activation and brain volume, we show that multivariate measures of all three features—brain activation, brain volume, and behavior—can accurately predict an individual’s sex, but that the degree of a person’s sex typicality for one set of features is largely uncorrelated with that of another. The weak interindividual coupling between sex typicality scores for measures of regional brain activation and volume echoes the weak spatial coupling between these sex differences across the cortex. Sex typicality scores were even weakly correlated between multivariate measures of brain activation from fMRI of different tasks—indicating, for example, that a female with highly female-typical patterns of brain activation during a gambling task may have less female-typical patterns of activation during a social task. The inclusion of non-volumetric measures such as surface area and cortical thickness does not alter these findings. Taken together, these observations suggest that the degree of sex-biased functional organization within a person’s brain is highly context-dependent and untethered from the degree of their sex typicality in brain anatomy and behavior. Indeed, less than 10% of individuals had sex typicality scores that were consistently concordant with their self-reported sex across all 16 feature sets (spanning brain activation, brain anatomy, and behavior) used for sex classification. These insights have important implications for how we think about the manifestation of sex as a biological construct in the brain. They also crucially inform future efforts to use sex as a potential stratifier in precision behavioral medicine because while a person’s sex (e.g., as defined by self-report, karyotype, or bodily anatomy) is a highly stable and relatively easily measured trait, their sex typicality appears to be highly variable across different measures of brain and behavior. Consequently, incorporation of sex as a predictive feature in precision behavioral medicine is likely to require effortful tailoring of sex typicality scores for context-specific measures of brain and behavior.

Fourth, our brain wide association study (BWAS) analyses provide several advances in the understanding of brain-behavior relationships in humans and their potential modulation by sex. As a foundation for analysis of sex differences, we extend upon recent high-profile BWAS studies of resting state fMRI data^66^ and establish that coherent BWAS signals can be detected for task fMRI data at sample sizes of ~500 individuals. We then reveal that the global topographies of behavior-activation associations are highly conserved between males and females across >90% of all examined combinations of behavioral scales and fMRI tasks, as are region-specific associations between interindividual variation in brain activation and behavior. Moreover, those few behavioral scales that do show subtle sex differences in the spatial patterning of their correlations with brain activation tend not to show sex differences in their mean value. Taken together, these observations indicate that: (i) relationships between brain activation and behavior are largely similar between males and females, and (ii) those few subtle differences that do exist are not concentrated in sex-biased behaviors.

Our overall finding of broad orthogonality between sex differences in brain activation, brain anatomy, and behavior is striking, and could reflect several possible scenarios that are not mutually exclusive. One theoretical possibility is that sex differences in the constructs we consider here are, in fact, interrelated, but that this is obscured by error in our current measurement tools. While there remain active concerns about the reliability and validity of measures from functional neuroimaging^75^, we note that sex-difference maps for most studied neuroimaging phenotypes showed high split-sample spatial reproducibility, and that reproducible correlations were evident between interindividual variation in brain activation and behavior within each sex. Nevertheless, it will be important to revisit this work as measurement accuracy and reliability improve. Another possibility is that—despite the unprecedented breadth of HCP fMRI task and behavioral data—there are some as-yet untested combinations of task paradigms and behaviors that could elicit prominent sex differences in the relationship between brain activation and behavior. Future tests of this idea could perhaps focus on sex-biased behaviors that are more pronounced^76,77^ and conserved across species (e.g., suckling, physical aggression) and the subcortical circuits that support these behaviors^78^. Moreover, task-based fMRI represents only one of many approaches to measuring brain function, and cortical morphology captures only a subset of anatomical variation. While these measures account for a substantial proportion of the human neuroimaging literature, other functional modalities (e.g., resting-state fMRI, Electroencephalography, or Magnetoencephalography) and structural measures (e.g., diffusion-weighted imaging, volume-based morphometry, or T1/T2 ratio) may show stronger concordance with behavior and greater sensitivity to sex modulation effects than those examined here. It may also be that sex differences in the neuroimaging and behavioral features examined herein show stronger inter-relationships during dynamic windows of sex-biased brain development^17,79,80^ and ageing^81^. It will be important to test these hypotheses in future work as necessary multi-task and multimodal datasets in these lifespan stages grow. Finally, there is also the more far-reaching possibility that in vivo neuroimaging features are fundamentally constrained in the proportion of interindividual behavioral variation that they can explain, which consequently limits their ability to detect group differences in brain-behavior correlations^66^.

Our findings should be considered in light of several key conceptual distinctions. First, the current study design is focused on the biological construct of sex and the specific contrast between self-identified males and females. There are multiple other ways of assigning sex (sex chromosome dosage, appearance of external genitalia, circulating hormone levels) that do not always result in the same groupings^82^. However, dissociations between these different definitions of sex are estimated to affect well below 1% of individuals^83^ and would therefore be unlikely to alter our findings. Second, it is important to underline that the focus of this study on the biological concept of sex rather than the psychosocial concept of gender. Gender-related measures may show distinct associations with brain activation, and testing this hypothesis would require dedicated study designs. Third, reporting a sex difference says nothing about the possible causes of that difference (be they gender- or sex-related) nor does it speak to the potential functional consequences—if any—of that phenotypic difference. As we hope to show here, separate, dedicated, analyses are needed to test for potential links between a sex-biased brain feature and human behavior, and there are numerous forms these links might take—each needing a dedicated statistical test. More fundamentally, strict causal tests of these brain-behavior hypotheses are hard to achieve in humans, where access to experimental methods is limited.

Notwithstanding these limitations and caveats, our study provides an unprecedentedly comprehensive and integrative analysis of sex differences in task-dependent brain activations, brain anatomy, and behavior in humans. Through this, we show that although robust and reproducible sex differences exist in all three of these phenotypic domains examined, the degree of an individual’s sex typicality is strikingly uncorrelated between domains—revealing these as orthogonal axes of sex-based phenotypic variation. Moreover, while we find that humans do show reproducible associations between regional brain activation and behavior, these are largely indistinguishable between males and females—except for a small subset of behavioral measures, which are mostly not themselves sex-biased but do show subtle sex differences in their associations with regional brain activation. These insights inform longstanding debates regarding the nature and behavioral relevance of sex differences in human brain function—helping to shape our current understanding and future queries of sex as a neurobiological variable.

## Methods

### Participants and data preparation

#### Participants

We leveraged the HCP S1200 release^35^ (March 2017) and included 978 healthy adults (455 males and 523 females) who completed all seven task fMRI scans and successfully passed a suite of stringent and systematic quality control procedures (including filters for head motion and several modality-specific quality metrics as previously detailed in ref.^84^. All of these individuals also had high-resolution T1-weighted 0.7-mm isotropic anatomical scans, and a comprehensive set of behavioral data. This dataset ensured complete multimodal data to allow tests for sex differences in brain activation, brain volume, and behavior (SDAs, SDVs, and SDBs) in the same cohort. Male (27.8 [3.7], mean[SD] yrs) and female (29.5 [3.6] years) participants in this final cohort differed slightly in mean age (*P* < 0.05 in a two-sample t test), but all fell within the same age range (22–37 years), and we controlled the age effect in the following models to determine sex differences. Procedures for recruitment of HCP participants are detailed in ref.^35^.

We additionally leveraged the UK Biobank dataset^65^ (www.ukbiobank.ac.uk) as an independent replication cohort in sensitivity analyses designed to validate the regional distribution of sex differences and sex prediction performance in brain structure observed in the HCP dataset. Because the structure of task fMRI and behavioral data differs substantially between the HCP and UK Biobank datasets, these validation analyses were restricted to brain anatomy, for which high-quality structural MRI data are available in both cohorts. To closely match the age range and degrees of freedom between datasets, we selected the youngest subset of healthy UK Biobank participants (44–50 years; 529 males and 637 females) who were scanned at a single site (site #11025). T1-weighted anatomical images (1-mm isotropic resolution) from these participants were processed using the same structural MRI pipeline applied to the HCP dataset. Details regarding UK Biobank participant recruitment and data acquisition procedures are described in Miller et al. 2016^85^.

Analyses of the HCP and UK Biobank data and the research protocol were approved by the institutional review board at the National Institute of Mental Health. Informed consent was obtained from all participants, and sex was self-reported.

#### Task fMRI data

The set of seven task fMRI experiments included in the HCP study design had been carefully selected to evoke reliable activations in as many well-characterized neural systems as possible within tolerable participant’s burden^38^ and thus included: emotion, gambling, relational, motor, social, language, working memory (full details of paradigms and main contrasts in Supplementary Fig. 1). Together, these seven task fMRI scans yielded 48 min 16secs of fMRI data from each participant gathered on the same customized 3 T Siemens Skyra using a slice-accelerated, multiband, gradient-echo, echo planar imaging sequence, offering an unusually high spatial (2 mm^3^) and temporal (TR = 720 ms) resolution^38^. We directly used the mean effects of activity estimated for an individual participant across two runs for a specific task, outputs of the HCP preprocessing pipelines v3 that were specially designed to capitalize on these high-quality data^36^. In brief, these within-subject fixed-effects analyses include the minimal preprocessing pipeline^36^ for motion correction, distortion correction, registration to standard space, and high-pass filtering at 200 s, and smoothing to 4 mm Full Width at Half Maximum, slice timing and temporal autocorrelation correction, and model estimation using a general linear model implemented in FSL’s FILM^86^. We selected the spatial registration implemented via the Multimodal Surface Matching all method that leverages cortical areal features derived from folding, myelin, and resting-state fMRI in a joint multimodal registration for improved intersubject registration of task fMRI datasets^37^. For the benefits of the sign-to-noise (SNR) increase and dimensionality reduction while preserving individual differences, our subsequent imaging analyses were conducted in the gold-standard HCP-multi-modal parcellation 1.0 cortical parcellation (180 regions per hemisphere) derived directly from the multi-model HCP data^37^. These individual-level measures of task-evoked cortical activity—within-subject fixed effects of activation in the HCP parcellation per participant and per task—were inputs for the downstream fMRI analyses described below.

#### Structural MRI data

High-resolution anatomical images were collected using T1-weighted MPRAGE at 0.7 mm and preprocessed using the PreFreesurfer pipeline^36^. Next, for accurate inter-subject alignment while preserving the advantage of high resolution, we selected the cortical surface based FreeSurfer v7^56–58^ recon-all with the *highres* flag to reconstruct and parcellate the cortex of all individuals^87^—extracting regional cortical GMV using the HCP parcellation. Total GMV was computed as a global measure. To complement the primary volumetric analyses, we additionally extracted two anatomical measures—surface area and cortical thickness—for supplementary analyses described below. In addition, we excluded 15 participants (resulting in 444 males, 519 females) with an Euler number less than—217—a conservative threshold suggested by Rosen et al.^88^ to ensure the high quality of segmentation and registration.

Notably, the three morphometric measures examined here, cortical volume, surface area, and cortical thickness, extend the assessment of sex differences in cortical anatomy beyond the GMV analyzed in our previous study^50^. Importantly, in the current work, cortical volume was derived as the product of cortical thickness and surface area using a surface-based FreeSurfer pipeline, representing the volume of the cortical sheet at each vertex while accounting for cortical folding. This measure is therefore not equivalent to the voxel-based GMV used previously^50^, which was derived from volume-based morphometry and reflects the fraction of gray matter within each voxel in three-dimensional space without explicit consideration of cortical geometry. These methodological differences and associated non-equivalence of regional GMV differences from surface- vs volume-based morphometry mean that the two methods cannot be assumed to generate identical group difference maps^89^.

#### Behavioral data

Following the same rationale behind development of task fMRI experiments, the HCP consortium collected a reliable and well-validated battery of measures that assess a wide range of human behavioral traits^35^. Using the criteria previously suggested by ref.^90^, we considered 86 scales from the HCP battery under the following 9 categories: alertness, cognition, emotion, motor, personality, sensory, psychiatric and life function, substance use, and scanner tasks for the following analyses. All included behavioral scales met the following criteria: (i) were numerical; (ii) had <10% missing values across participants, and (iii) did not constitute a composite that would introduce numerical redundancy with other included scales. For scales provided as both raw scores and scores adjusted for sex, raw scores were used to enable estimation of SDBs.

### Analysis of fMRI data

Before testing for SDAs, we first verified the expected^38–43^ group average (males and females combined) patterns of brain activation and deactivation associated with each of the seven fMRI contrasts after false discovery rate (FDR) correction for multiple comparisons across 360 cortical regions for each task, with *q* (the expected proportion of falsely rejected nulls) set at 0.05 (Supplementary Fig. 1 and Data 1). This same statistical threshold was used for all subsequent regional analyses in the HCP parcellation, and all data analyses and cortical map visualizations were conducted in R version 4.4.3^91^ and Connectome Workbench 1.5^92^ (respectively) unless otherwise specified.

#### Testing for task-specific and task-general SDA

We first harnessed the full fMRI dataset across all seven tasks to test for potential task-specific SDAs in each region of the HCP cortical parcellation. Then, for any regions without statistically significant task-specific SDAs, we tested for task-general SDA (i.e., generic SDAs that are present across all tasks). Both analyses were implemented using linear mixed models (*lmer* in package lme4^93^) to estimate activation of all seven tasks at each HCP region, with task-specific and task-general SDAs being estimated by the models below (task-specific SDA: *β3* coefficient in Model 1 and task-general SDA: *β1* in Model 2):

1 $$	{\mathrm{Model}}\,1 \\ 	 \,{\mathrm{Brain}} \,{\mathrm{activity}} \sim {{\rm{\beta }}}0({\mathrm{intercept}})+{{\rm{\beta }}}1({\mathrm{Sex}})+{{\rm{\beta }}}2({\mathrm{Task}}) \\ 	+{{\rm{\beta }}}3({\mathrm{Sex}}\times {\mathrm{Task}})+{{\rm{\beta }}}4({\mathrm{Age}})+{\mathrm{Participants|Family}}$$

2 $$	{\mathrm{Model}}\,2\, \\ 	 {\mathrm{Brain}} \,{\mathrm{activity}} \sim \beta 0({\mathrm{intercept}})+\beta 1({\mathrm{Sex}})+\beta 2({\mathrm{Task}})+\beta 3({\mathrm{Age}}) \\ 	+{\mathrm{Participants|Family}}$$

We additionally tested for a sex-by-age interaction by including a Sex × Age term in Model 1; as this interaction was not significant in any brain region, it was excluded from the final model.

We applied FDR correction (*q* < 0.05) to identify regions with statistically significant task-specific SDAs (Fig. 1a and Supplementary Data 2) and task-general SDAs (Fig. 1d and Supplementary Data 5). To aid interpretation of these cross-task effects, we generated *post hoc* SDA maps for each individual task using the linear model below (SDA: *β1* coefficient in Model 3, Supplementary Fig. 2 and Data 3):

3 $$	{\mathrm{Model}}\,3 \\ 	 \,{\mathrm{Brain}} \,{\mathrm{activity}} \sim \beta 0({\mathrm{intercept}})+\beta 1({\mathrm{Sex}})+\beta 2({\mathrm{Age}})$$

#### Effect sizes and reproducibility of sex differences in brain activation

Effect sizes of task-specific and task-general SDAs were estimated using the partial eta-squared metric (*eta_squared* function in package effectsize^94^), which ranges from 0 to 1, with the following effect-size brackets: <0.01, small; 0.01–0.06, medium; >0.14, large. This metric estimated the proportion of variance in a dependent variable (brain activity) explained by a specific independent variable, after accounting for the variance explained by other independent variables in the model (Supplementary Fig. 5a, b and Data 2 and 5). Effect sizes for SDAs in each task were estimated using the Cohen’s *d* metric (*t_to_d* function in package effectsize^94^) converted from the *t* values of sex effects for model 3 (Supplementary Data 3). This metric also captures the variance in a dependent variable (activity) explained by a specific independent variable, after accounting for the variance explained by other independent variables, and has the following effect size brackets: <0.2, small; 0.2–0.5, medium; >0.8, large.

The spatial reproducibility of task-specific and task-general SDAs was determined by comparison of maps for these effects (Pearson’s correlation across 360 HCP regions for Model 1 *β3 *F statistic and Model 2 *β1* t statistic, respectively, between 1000 random split-haves of the HCP datasets (Supplementary Fig. 5c, d).

### Clustering task-specific sex differences in brain activation 

We used a kmeans clustering approach to partition brain regions showing statistically significant task-specific SDAs (*n* = 296) into subsets with different profiles of SDAs across the seven tasks. Thus, the input matrix for this clustering was a 296 × 7 matrix containing the effect size for SDA in each region for each task. A range of k-values was tested using the *kmeans* function in R with nstart and iter.max set at 100 and scree plot inspection indicated an optimal 4-cluster (Supplementary Fig. 4a). We examined clustering separation and stability using *prcomp* function (package stats^91^) (a 2-dimensional principal components analysis plot, Supplementary Fig. 4b) and *bootstrapStability* function (package bluster^95^) (a heatmap with higher values indicating more stability within and between clusters, Supplementary Fig. 4c). Taking brain regions defined by each cluster as an independent region-of-interest (ROI), we calculated the average of activity level in these ROIs across males and females per task (Supplementary Fig. 4d) and compared their differences between sexes (Fig. 1c) using regression models (*lm* function) with control of age and statistical significance determined by Bonferroni correction for 28, 4 clusters * 7 tasks (indicated by asterisks in Fig. 1c and Supplementary Fig. 4d and Data 4).

#### Testing for alignment of task-specific and task-general sex differences in activation  with functional anatomy of the human brain

We used spatial permutation tests (Spin Tests^44,45^) to determine if the spatial patterning of task-specific and task-general SDAs was aligned with known functional anatomy of the human brain as defined by two complementary approaches: meta-analysis of regional activation across >11k fMRI studied (Neurosynth^46–48^, Fig. 1f) and functional connectivity during resting state FMRI (rsFMRI, Yeo-Kreinen 17 network parcellation^49^, Supplementary Fig. 6). Both of these cortical annotations can be expressed as a set of binary assignments for HCP parcellation cortical regions: one for each of 24 meta-analytic fMRI “topics” (e.g., an “action,” “actions,” and “motor” topic or a “memory,” “encoding,” and “retrieval” topic) distinguishing regions that are activated by fMRI tasks involving that topic vs regions that are not^48^; and, one for each of the 17 Yeo-Krienen networks (in vs out of network). For each of these two sets of binary cortical annotations, Fisher’s Exact Tests were used to calculate odds ratios of spatial overlap between each binary annotation (i.e., a Neurosynth topic map or a Yeo network) and each of the 5 sets of cortical regions defined by tests for SDA above: the 4 clusters of task-specific SDA (Fig. 1b) and the set of regions showing task-general SDA (Fig. 1d). Spin Tests^44,45^ were used to assess statistical significance of these observed odds ratios by comparing them to null distributions of odds ratios from 10,000 rotations/spins of the HCP parcellation using a python toolbox (markello_spatialnulls)^45^. This procedure yielded empirical P_spin_ values for the statistical significance of overlaps between the 5 SDA clusters and the 24 Neurosynth topic activation maps, as well as the 17 Yeo-Krienen maps (with Bonferroni testing applied for multiple comparison across 24 and 17 maps, respectively). Statistically significant alignments of SDAs with Neurosynth and Yeo-Krienen annotations are shown in Supplementary Fig. 6.

### Analysis of structural MRI data

All participant T_1_-weighted structural MRI scans were submitted to the aforementioned FreeSurfer pipeline for surface-based estimation of regional GMV in each HCP cortical region and total cortical GMV as the sum of these values. Sex differences of regional GMV were estimated using the following linear model [Sex Differences in Volume (SDVs) estimated by the *β1* coefficient].

4 $$	{\mathrm{Model}}\,4 \\ 	 \,{\mathrm{GMV}} \sim {{\rm{\beta }}}0({\mathrm{intercept}})+{{\rm{\beta }}}1({\mathrm{Sex}})+{{\rm{\beta }}}2({\mathrm{Age}})+{{\rm{\beta }}}3({\mathrm{total\; GMV}})$$

We used FDR correction for multiple comparisons (*q* < 0.05) to define cortical regions with statistically significant SDV above and beyond sex differences in total GMV (Fig. 2a), and the effect size of SDV in these regions (Supplementary Fig. 7) was estimated as the Cohen’s *d* value for group differences using the *t_to_d* function in the package effectsize^94^). The same Model 4 was applied to surface area and cortical thickness, controlling for the corresponding global measures (total surface area and mean cortical thickness), to identify regional sex differences in non-volumetric aspects of brain anatomy.

### Testing for spatial correspondence between sex difference in brain activation and volume 

We used Spin Tests^44,45^ (see above) to assess of the spatial patterning of task-specific SDAs (Fig. 1a) and task-general SDAs (Fig. 1d) was statistically significantly aligned with that of SDVs (Fig. 2a) (conjunction results in Fig. 2b–e and Supplementary Data 6). Spatial comparison between task-specific SDAs and SDVs was assessed by two complementary analyses: (1) estimating *P*_spin_ by comparing the observed odds ratio from a Fishers Test of overlap between all regions of statistically significant task-specific SDAs and all regions of statistically significant SDVs (regardless of sign) to a null distribution of odds ratios from 10k Spin Test rotations of the task-specific SDA map (Fig. 2b, c); and, (2) estimating *P*_spin_ (Supplementary Fig. 8d) by comparing the observed cross-region Pearson’s correlation coefficient (Supplementary Fig. 8c) between *F* values for task-specific SDAs (from *β3* coefficient in Model 1, Supplementary Fig. 8a) and *T* values for SDVs (from *β1* coefficient in Model 4, Supplementary Fig. 8b) to a null distribution of correlation coefficients from 10k Spin Test rotations of the task-specific SDA map.

Spatial alignment between task-general SDAs and SDVs was assessed by computing the observed odds ratio from a Fisher's Test of overlap between all regions of statistically significant task-general SDAs (regardless of sign) and all regions of statistically significant SDVs (regardless of sign) and comparing this to a null distribution of odds ratios from 10k Spin Test rotations of the task-general SDAs map. Given that this test revealed a significant overlap (*P*_spin_ = 0.006, Fig. 2d, e), we further probed for evidence of correlation (Supplementary Fig. 9b) across individuals between (i) the average cross-task brain activation (the average of standardized within-subject fixed effect over seven fMRI tasks), and (ii) brain volume (the average of standardized relative GMV controlling for total GMV), over brain regions in this overlap that are both female-biased (green in Fig. 2d except the left posterior insular area).

### Analysis of behavioral data

We tested for SDBs across 86 behavioral scales selected above (Supplementary Data 7). Effects of sex were estimated on these scales using linear regression (lm function in R^91^), except for the 29 scales representing count data, for which we used a generalized linear model (glm with a Poisson distribution^91^). In both, age was controlled as a covariate, and Cohen’s *d* was calculated for effect size. Statistical significance was determined after Bonferroni correction across the 86 scales (*P* < 0.05/86, Fig. 3a and Supplementary Data 7). Scales for reaction times and error measures, were inverted for data visualization (such that higher values indicate better performance, Fig. 3a) and are denoted as such in Supplementary Data 7.

### Predicting sex using fMRI, structural MRI, and behavioral data

We applied machine learning to quantify how accurately fMRI, structural MRI, and behavioral data could predict a participant’s sex, and derive person-level sex typicality scores (STSs) for these data modalities. Given our interest in the degree of interindividual correlation in STSs between data modalities, we applied these machine learning analyses in the 963 HCP participants who had complete data for all three modalities (444 males, 519 females).

Machine learning was implemented in the caret R package^64^—yielding percentages per participant for the likelihood of being male or female. The percentages sum to 100, and the predicted sex for an individual is given by the higher percentage. We applied a PLSDA model with a sampling scheme of repeated 10-fold cross-validation and the partial least squares algorithm for model tuning. The PLSDA model was tuned over the number of PLS components that should be retained to optimize the performance measured by the area under the receiver operating characteristic (ROC) curve. PLSDA combines dimensionality reduction and discriminant analysis into one algorithm and is widely used in predictive modeling for high-dimensional data because it does not assume the data to fit a particular distribution and thus is flexible, and is linear in their parameters, therefore easily interpretable. We applied the PLSDA algorithm to the 12 multivariate datasets (italicized below) drawn from fMRI, structural MRI and behavioral measures as follows: (i) fMRI measures—fMRI activity across all seven tasks over brain regions showing statistically significant *task-specific* SDAs (963 participants of 296 regions × 7 tasks combinations) and statistically significant *task-general* SDAs (963 participants of 14 regions × 7 tasks combinations), as well as activity in set of brain regions showing statistically significant SDAs in a single task [*emotion* (963 participants of 67 regions), *gambling* (963 participants of 196 regions), *language* (963 participants of 121 regions), *motor* (963 participants of 40 regions), *social* (963 participants of 116 regions), *working memory* (963 participants of 21 regions)]; (ii) structural MRI measures—*relative GMV* controlling for total GMV and *raw GMV* without controlling for total GMV in regions showing statistically significant SDV (963 participants of 97 regions); (iii) Behavioral measures—*all scales* showing statistically significant SDBs (963 participants of 15 behavioral scales) and *all without strength* (963 participants of 14 behavioral scales) given the outlier effect size for sex differences shown by this behavioral scale. Note that for sex classification based on behavioral scales, only continuous behavioral scales were used (given the incompatibility of count data with PLSDA prediction models), and missing values were imputed using Multiple Imputation by Chained Equations (MICE, *mice* function in package mice^96^). For all models, the observed accuracy of sex prediction was plotted against the null distribution generated by randomly shuffling sex labels of participants 10k times and repeating sex prediction in these shuffled datasets (Fig. 3b).

### Testing inter-domain correlations of sex typicality across individuals

The 12 PLSDA analyses detailed above each generated a prediction of sex for each participant in the form of sex probability scores that summed to 1. These prediction scores provided STSs in the [0, 1] range, representing the probability assigned to a person for their belonging to their true sex group (i.e., a male assigned a 0.8 probability of being male would have a 0.8 STS). Consequently, our PLSD analyses generated 12 STSs for each participant—one for each of the input data matrices used for sex prediction as detailed above. We used Pearson’s correlations between these 12 STSs (Fig. 3c, d) to quantify the extent to which an individual’s sex typicality for one data set of features (e.g., fMRI measures of brain activation in regions of statistically significant task-specific SDAs) correlated with that based on another (e.g., structural MRI measures of brain volume in regions of statistically significant SDVs). Statistical significance (*P*_perm_) was determined nonparametrically by comparing each observed STS correlation to a null distribution of STS correlations from 10k repeated PLSDA analyses following permutation of sex labels. Incorporating the additional anatomical measures of surface area and cortical thickness increased the number of STSs from 12 to 16 (Supplementary Fig. 10 and Data 8). Finally, for males and females separately, we plotted person-level STSs across these 16 models and computed the percentage of individuals whose sex was misclassified in 0, 1, 2, …, 16 models (Supplementary Fig. 11).

### Cross-validation of anatomical sex differences and sex prediction in the UK Biobank dataset

In two sensitivity analyses, we assessed the spatial reproducibility of sex differences in brain anatomy using an independent UK Biobank dataset and evaluated whether sex prediction performance in the HCP dataset was affected when the masks of significant sex differences in brain anatomy used to define training data were derived from the UK Biobank dataset (as opposed to an HCP dataset mask as in our primary analyses).

Structural MRI data from the UK Biobank sample were preprocessed using the same FreeSurfer pipeline applied to the HCP dataset. Two participants were excluded due to poor surface reconstruction quality (Euler number < −217), resulting in a final sample of 528 males (49.0 [1.0] years) and 636 females (48.9 [1.1] years). Using the same Model 4, we identified significant regional sex differences (q < 0.05) in GMV, surface area, and cortical thickness beyond global measures in the UK Biobank sample (Supplementary Fig. 12a).

First, to quantify the spatial correspondence of sex differences between the HCP and UK Biobank datasets, we computed spatial correlations of effect sizes (Cohen’s *d*) across the 360 regions defined by the HCP parcellation. Statistical significance of this spatial correlation was assessed using spin tests with 10k spatial permutations (Supplementary Fig. 12b).

Second, to address potential inflation of sex prediction accuracy resulting from using the same dataset to define sex differences and perform prediction, we repeated the machine-learning analyses described above using masks of significant sex differences derived from the UK Biobank dataset (Supplementary Fig. 12c), rather than from the HCP dataset (original results shown in Supplementary Fig. 10c). We then quantified changes in prediction accuracy between these two conditions (Supplementary Fig. 12c). We also compared model outputs by computing Pearson correlations of STS across participants between the primary model based on a sex difference mask defined in the HCP and the alternative model where this mask was defined in the UK Biobank (Supplementary Fig. 12c).

### Brain-wide association studies (BWASs) between activation and behavior

#### Sex stratified BWAS

We conducted BWASs between brain activation and behavior for each fMRI task and separately within each sex—yielding 14 (7 fMRI tasks in 2 sex groups) 360 × 57 matrices of *P* values for the observed association at each cortical region (*n* = 360) between interindividual variation in brain activation and interindividual variation in each of the behavioral measures that existed in a continuous scale (*n* = 57, Fig. 4a and Supplementary Data 9). As an initial omnibus test for global evidence of enriched activation-behavior associations in each of these 14 matrices, we compared the observed count of matrix cells showing nominally significant activation-behavior associations to a null distribution of 10k counts from permutations of behavioral scores across individuals within each sex group to derive a *P*_perm_ value for each matrix. Statistical significance of these 14 *P*_perm_ values was determined using Bonferroni correction (*P*_perm_ < 0.05/14). We next tested reproducibility (Supplementary Fig. 13) of these nominally significant activation-behavior associations in each matrix—randomly splitting males and females into halves separately 1k times and repeating BWAS calculations to identify *t* values of activation-behavior associations in each half and computing Pearson’s correlation coefficients of t statistics between halves across regions and behaviors where nominally significant activation-behavior associations were originally observed in the full samples. Given the clear columnar structure of activation-behavior associations in the 14 matrices, we also used the 10k permutations of behavioral scores to compare the observed count of nominally significant (*P* < 0.05) activation-behavior associations for each behavior (i.e., each matrix column), to association counts from the 10k permutations—thereby deriving a *P*_perm_ value for each behavioral scale within each of the 14 matrices (Supplementary Fig. 14 and Data 9).

#### Comparing the topography of brain-behavior associations between sexes

The procedure above yielded two *P* value matrices of identical dimensions (360 regions × 57 tasks) for each of the 7 fMRI tasks—one representing the significance of activation-behavior associations in males and one in females. After replacing *P* values with their associated coefficients (*t*-statistics from the same linear models estimating behavior with regional activation), the correlations between corresponding columns of the two matrices quantify the topographic similarity of brain-behavior associations between sexes—the extent to which the spatial patterning of activation-behavior associations across the cortical sheet is similar between males and females. We used a permutation procedure to derive a topographical divergence *z* score (TDS), estimating the degree to which the observed topographies of activation-behavior associations are more dissimilar between males than females that would be predicted under the null. Specifically, we generated a null distribution of columnar associations from re-estimation of paired 360 × 57 activation-behavior associations after 10k permutations of sex. For each column pair, the observed correlation between sexes was compared to the distribution of 10k null correlations to derive a TDS as (mean of null correlations – observed correlation) / standard deviation of null correlations.

TDS scores were calculated for all 57 behavioral scales in each fMRI task (Fig. 4b and Supplementary Data 10). We examined whether the TDS score is significantly larger than the expected value (indicating that the observed correlation is significantly less than the mean of the null distribution from 10k permutations of sex). Bonferroni correction for 57 scales and 7 tasks was applied to determine the statistical significance of each TDS score (*P*_perm_ < 0.05/399).

### Sex modulatory effects in BWAS

We directly modeled sex interaction effects in BWAS for each fMRI task, yielding 7360 × 57 matrices of *P* values for the β3 coefficient from the linear model below:

5 $$	{Model}\,5 \\ 	 \,{{\rm{behavioral\; performance}}} \sim {\beta }0({{\rm{intercept}}})+{\beta }1({{\rm{brain\; activity}}}) \\ 	+{\beta }2({{\rm{sex}}})+{\beta }3({{\rm{brain\; activity}}}*{{\rm{sex}}})$$

The same omnibus tests, illustrated previously, were applied here to examine the overall sex modulatory effects on activation-behavior associations in each fMRI task, and the null distributions that were generated by 10k sex shuffling, in combination with Bonferroni correction for seven tasks, were used to determine their statistical significance (Supplementary Fig. 15a, b). A refined columnar analysis was applied here as well to dissect sex modulatory effects per behavioral scale for each task—the observed count of nominally significant sex modulatory effects across 360 cortical regions of the HCP parcellation in each behavioral scale was compared against 10k counts from the sex-shuffling null distribution to generate a *P*_perm_ value for each behavioral scale and Bonferroni correction for 399 pairs of associations of 57 behavioral scales and 7 tasks was used to determine the statistical significance (Supplementary Fig. 15c and Data 11).

### Reporting summary

Further information on research design is available in the Nature Portfolio Reporting Summary linked to this article.

## Supplementary information

Supplementary Information

Description of Additional Supplementary Files

Supplementary Dataset 1

Supplementary Dataset 2

Supplementary Dataset 3

Supplementary Dataset 4

Supplementary Dataset 5

Supplementary Dataset 6

Supplementary Dataset 7

Supplementary Dataset 8

Supplementary Dataset 9

Supplementary Dataset 10

Supplementary Dataset 11

Reporting Summary

Transparent Peer Review file

## Source data

Source Data

## Data Availability

The primary HCP data are available by application at https://www.humanconnectome.org/study/hcp-young-adult. The UK Biobank validation data can be accessed by submitting an application at https://www.ukbiobank.ac.uk/enable-your-research/apply-for-access. The data generated in this study are provided in the accompanying Supplementary Data and Source Data files. Source data are provided in this paper. Source data are provided with this paper.

## Code availability

All custom code and scripts used for data processing and statistical analysis in this study are publicly available on GitHub at https://github.com/siyuanliu8/HCPsexdifferences.
