# Supplementary material for: Robust but independent sex differences in human brain function, structure, and behavior
Source: Nat Commun. 2026 May 21;17:6694. doi: 10.1038/s41467-026-73262-2 (PMC13385741; doi:10.1038/s41467-026-73262-2)
Supplement: Supplementary file 1 — Supplementary Information [file 41467_2026_73262_MOESM1_ESM.pdf]

Supplementary Information

Robust but independent sex differences in human brain function, structure and behavior

Siyuan Liu[1], Bridget W. Mahony[1], Ethan T. Whitman[1], Stephen J. Gotts[2], Dustin Moraczewski[3], Adam Thomas[3], Alex Martin[2], Armin Raznahan[1*]

1 Section on Developmental Neurogenomics, Human Genetics Branch, National Institute of Mental Health, Bethesda, MD 20814, USA

2 Section on Cognitive Neuropsychology, Laboratory of Brain and Cognition, National Institute of Mental Health, Bethesda, MD 20814, USA

3 Data Science and Sharing Team, National Institute of Mental Health, Bethesda, MD 20814, USA

*Corresponding author

Supplementary Text 1

Alignment of sex differences in brain activation with canonical functional systems and networks

To better understand how sex differences in activation (SDAs) relate to independent descriptions of functional neuroanatomy, we used Spin Tests[1,2] (**Methods**) to compare the spatial distribution of SDA clusters (**Fig. 1b,d**) with meta-analytically defined maps of brain activation for 24 different neuropsychological domains derived from latent discriminant analysis of text and reported activations for >11k fMRI studies (Neurosynth[3–5], **Supplementary Fig. 6a**). All 4 task-specific and the task-general SDA cluster showed significant spatial overlaps with canonical functional brain systems — establishing a statistically significant concentration of SDAs with brain systems subserving motor function (K1, task-general SDAs), attention and working memory (K2, K3), language (K3), and social cognition (K3, K4). We also found task-specific and task-general SDAs to be enriched within distinct canonical functional brain networks defined by independent resting state fMRI data (Yeo-Krienen 17 network parcellation[6], **Methods, Supplementary Fig. 6b)**. These observations imply that distinct functional systems can be differentially activated by males and females during execution of the same task. For example, the K2 cluster of task-specific SDAs — which shows stronger mean activation in females than males during gambling – overlaps with brain systems that are involved in spatial attention and motor planning (**Supplementary Fig. 6a**) and reside within visual and dorsal attention resting state networks (**Supplementary Fig. 6b**). Thus, brain regions showing distinct SDA profiles are spatially aligned with distributed brain networks subserving different aspects of cognition, behavior and emotion. However, this spatial alignment does not speak to the relevance of SDAs for any cognitive, behavioral or emotional traits.

Supplementary Figures 1 to 15

Task	Paradigm	Selected Contrast	Activation Map
**Emotion**	Valence judgments using face-matching task vs. shape-matching task	faces - shapes	
**Gambling**	Gambling decision making task during a card guessing game	average of punish and reward	
**Language**	Story comprehension task vs. math task (addition or subtraction)	story - math	
**Motor**	Motor task with different body parts (left or right fingers, left or right toes, or tongue)	average of all body parts	
**Relational**	Judgments about relations among features vs. feature matching	relational - match	
**Social**	Judgements about mental vs. random interactions following presentation of video clips	theory of mind - random	
**Working Memory**	N-back task (2-back versus 0-back) embedded in category specific representation task (places, tools, faces and body parts)	2 back - 0 back	

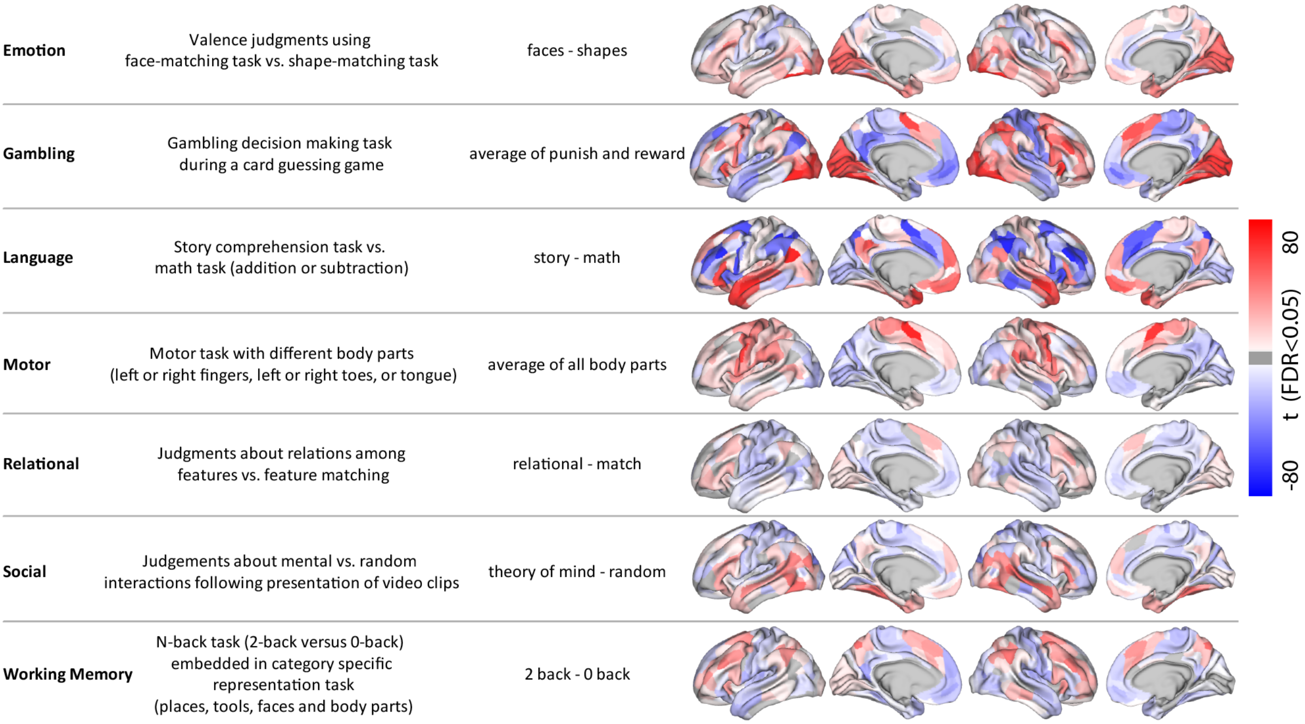

Supplementary Fig. 1: Characteristics and brain activation maps per fMRI task. This figure showing the paradigm, the main contrast selected to examine, and the statistically significant (FDR<0.05) brain activation map (t values of the selected contrast in the HCP parcellation) in all 978 participants for each of seven fMRI tasks including emotion, gambling, language, motor, relational, social and working memory. More details about task design and implementation are listed in the HCP reference manual (https://www.humanconnectome.org/storage/app/media/documentation/s1200/HCP_S1200_Release_Reference_Manual.pdf) and original publications[7–13].

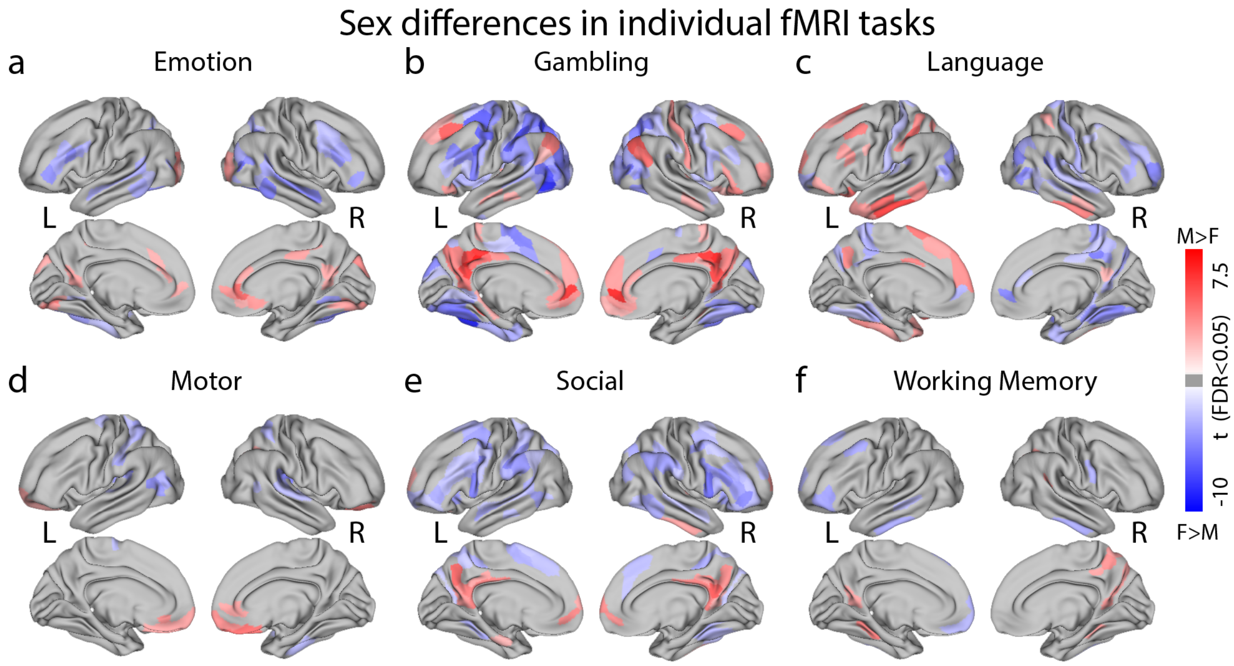

Supplementary Fig. 2: Sex differences in brain activation per fMRI task.
Using separate regression models with control for age, we detected statistically significant (FDR<0.05) sex differences (t values of males vs. females) in different cortical regions of the HCP parcellation during each fMRI task of emotion (**a**), gambling (**b**), language (**c**), motor (**d**), social (**e**), working memory (**f**). No significant sex differences in brain activation were found in the relational processing fMRI task.

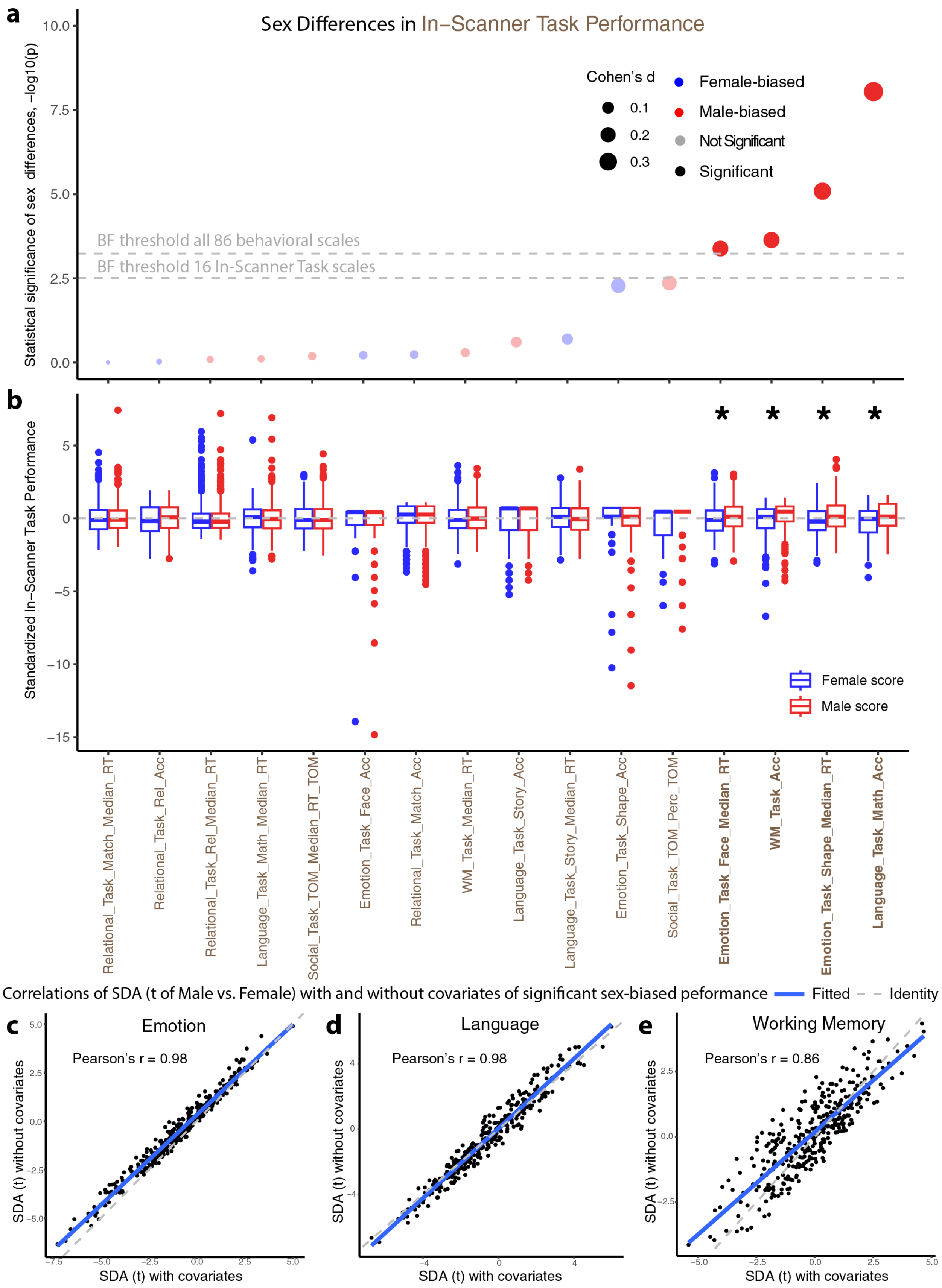

a
Sex Differences in In–Scanner Task Performance
Statistical significance of sex differences, −log10(p)
10.0
7.5
5.0
2.5
0.0
Cohen's d
0.1
0.2
0.3
Female-biased
Male-biased
Not Significant
Significant
BF threshold all 86 behavioral scales
BF threshold 16 In-Scanner Task scales
b
Standardized In–Scanner Task Performance
5
0
−5
−10
−15
*
Female score
Male score
Relational_Task_Match_Median_RT
Relational_Task_Rel_Acc
Relational_Task_Rel_Median_RT
Language_Task_Math_Median_RT
Social_Task_TOM_Median_RT_TOM
Emotion_Task_Face_Acc
Relational_Task_Match_Acc
WM_Task_Median_RT
Language_Task_Story_Acc
Language_Task_Story_Median_RT
Emotion_Task_Shape_Acc
Social_Task_TOM_Perc_TOM
Emotion_Task_Face_Median_RT
WM_Task_Acc
Emotion_Task_Shape_Median_RT
Language_Task_Math_Acc
Correlations of SDA (t of Male vs. Female) with and without covariates of significant sex-biased peformance
Fitted
Identity
c
Emotion
Pearson's r = 0.98
SDA (t) without covariates
SDA (t) with covariates
d
Language
Pearson's r = 0.98
e
Working Memory
Pearson's r = 0.86

Supplementary Fig. 3: Sex differences in brain activation per fMRI task exist above and beyond small and isolated sex differences in task performance.

(**a**) A ranked circle plot showing sex differences in 16 raw, unadjusted, numerical, behavioral scales that were collected accompanying task fMRI scans. Scales are ranked on the x axis by decreasing statistical significance of the sex difference [-log10(P) value on y-axis]. These 16 in-scanner task performance measures (color matches its category color in **Fig. 3a**) are part of the complete set of 86 behavioral scales (**Fig. 3a**, **Supplementary Data 7**) and processed in the same way (**Methods**, *Analysis of behavioral data*). Bonferroni corrections for both 86 and 16 comparisons (denoted by the two dash grey lines) are used to determine the statistical significance, yielding the same results — only four of 16 scales (marked by non-transparent dots) from three different fMRI tasks are significant. Dot size and color encode the Cohen's d effect size and direction (red: male>female; blue: female>male) of the sex difference, respectively. (**b**) A box plot showing the mean and range of standardized scores of these 16 scales in males and females. Note that sex differences in four significant scales are limited with weak to moderate effects (Cohen's d from 0.2 to 0.5). Although these four significant ones are male-biased, there is no clear trend in directionality of sex biases for task performance across all 16 scales. For example, males perform significantly faster than females in both shape and face matching during the emotion task fMRI (Emotion_Task_Shape/Face_Median_RT), but worse in accuracy albeit not significantly (Emotion_Task_Shape/Face_Acc). (**c**,**d**,**e**) Dot plots showing spatial correlations of sex differences (t values of males vs. females) across 360 cortical regions in the HCP parcellation without (y-axis, surface projections in **Supplementary Fig. 2**) vs. with (x-axis) covariates of these significant sex-biased performance measures in three fMRI tasks [**c**, covarying reaction time of shape and face matching in the emotion task fMRI (Emotion_Task_Shape/Face_Median_RT); **d**, covarying accuracy of mathematical task in the language task fMRI (Language_Task_Math_Acc); **e**, covarying accuracy of n-back task in the working memory task fMRI (WM_Task_Acc). Strong spatial correlations (Pearson's r >0.86) of SDA maps with and without covarying sex-biased task performance indicate that sex differences in brain activation (**Supplementary Fig. 2**) exist above and beyond small and isolated sex differences in task performance. Source data are provided as Source Data files.

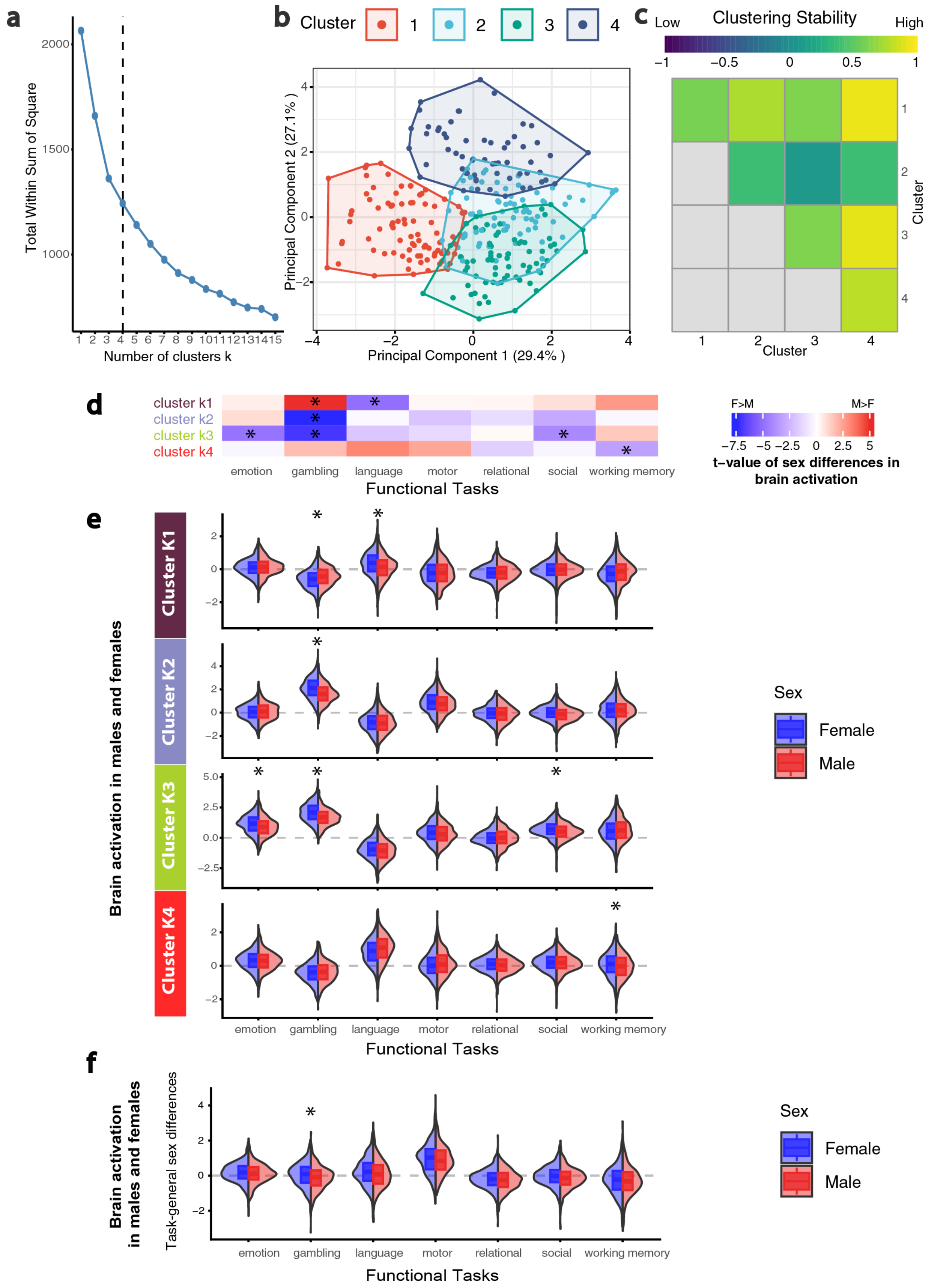
a
Total Within Sum of Square
Number of clusters k
b
Cluster
Principal Component 1 (29.4%)
Principal Component 2 (27.1%)
c
Clustering Stability
Low
High
Cluster
d
cluster k1
cluster k2
cluster k3
cluster k4
emotion
gambling
language
motor
relational
social
working memory
Functional Tasks
F>M
M>F
t–value of sex differences in brain activation
e
Cluster K1
Cluster K2
Cluster K3
Cluster K4
Brain activation in males and females
Sex
Female
Male
f
Brain activation in males and females
Task-general sex differences

Supplementary Fig. 4: k-mean stability of task-specific sex differences in brain activation and sex-stratified brain activation profiles across tasks.

(**a**) The scree plot used for selection of a four-cluster solution in k-means clustering of 296 regions showing significant task-specific sex differences in activation (**Fig. 1b**) based on their regional profiles of sex differences in activation across seven tasks (i.e., a matrix of effect sizes representing males vs. females in activation over 296 brain regions across the seven fMRI tasks). (**b**) Principal component analysis was conducted on the same matrix and the plot of the first two principal components shows separation of these four clusters. (**c**) Using bootstrapping in the bluster R package, we calculated clustering stability of this k-means solution: larger values on the diagonal indicate greater cluster stability across bootstraps and large values off the diagonal indicate greater cluster separability across bootstraps. Most clusters show high internal stability and high inter-cluster separability. (**d)** Heatmap showing t values of sex differences in brain activation across tasks for all four clusters of task-specific sex differences in activation (**Fig. 1b,c**), facilitating comparisons across clusters. (**e,f**) Combined violin and box plots showing male (in red) vs. female (in blue) distributions of brain activation in each task for the four clusters of task-specific sex differences in activation (**Fig. 1b,c**) and for regions exhibiting task-general sex differences in activation (**Fig. 1d,e**). Consistent with their definitions, the four task-specific clusters show no consistent direction of sex bias across the seven tasks (panel **e**), whereas regions exhibiting task-general sex differences show consistently greater activation in females than in males across tasks (panel **f**). In **d-f**, Asterisks indicate statistically significant sex differences in activation for a given task following Bonferroni correction ($P < 0.05/7/4$ for the four clusters of task-specific sex differences in activation in panel **d, e**; $P < 0.05/7$ for regions exhibiting task-general sex differences in activation in panel **f**). Exact P values and effect sizes for all regions of interest are reported in **Supplementary Data 4**.

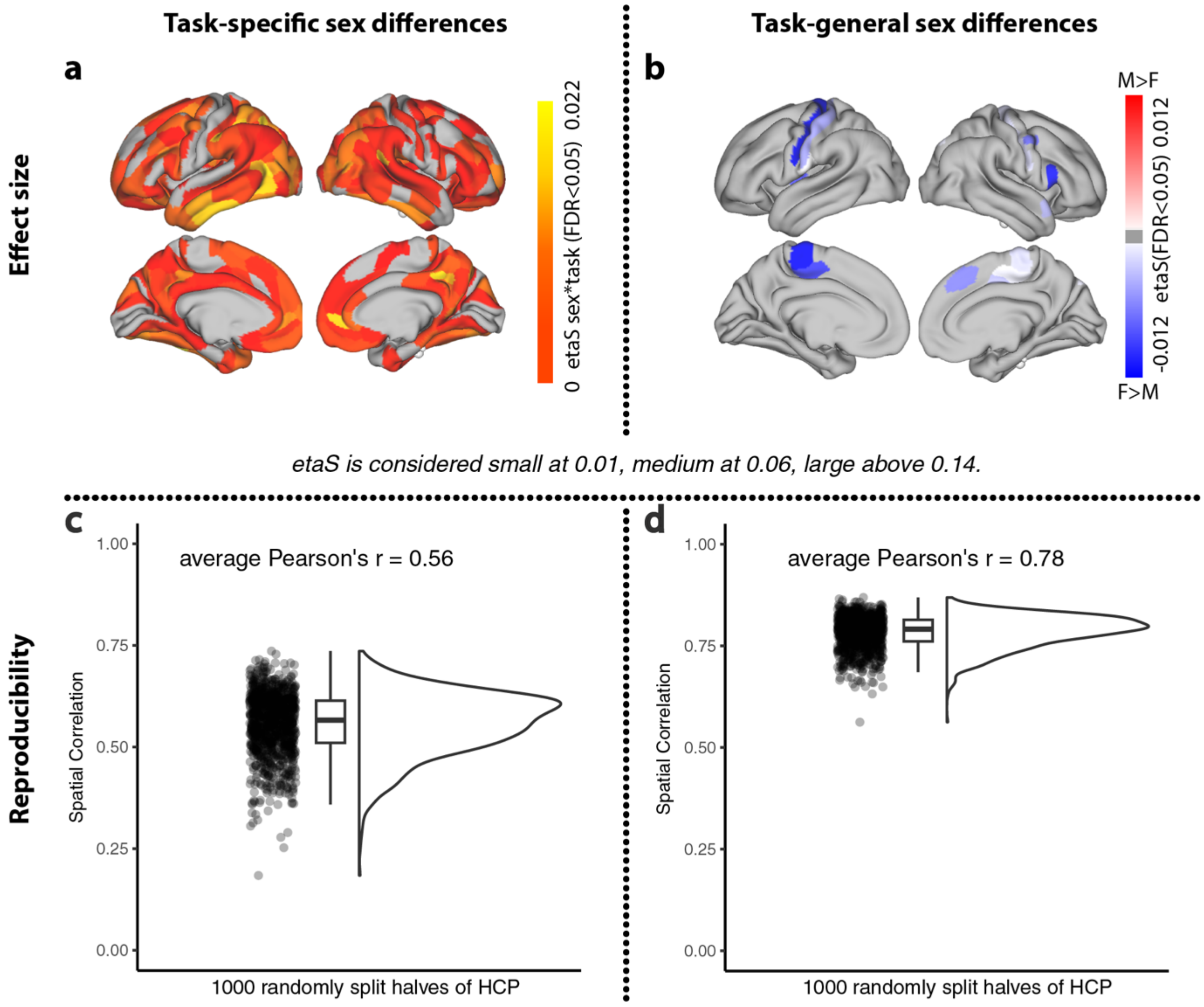

Supplementary Fig. 5: Reproducibility of task-specific and task-general sex differences in brain activation (task-specific and task-general SDAs, respectively) between independent split halves of the HCP dataset. (a, b) Partial eta-squared, an effect size measure in mixed-effect models, showing weak effect sizes (i.e. in 0.0021 – 0.022 range) of statistically significant task-specific SDAs (**a**) and task-general SDAs (**b**, with a negative sign to indicate female-biased). Regional statistics for these maps are provided in **Supplementary Data 2** and **5**. (**c, d**) Using 1k split-half tests, in each of which we randomly split the entire HCP sample (n=978) into halves with a sex ratio matching to the original one, we repeated the mixed-effect models to identify cortical maps of task-specific (F values of sex*task) and task-general (t values of males vs. females) sex differences in brain activation in each half and calculated Pearson's correlation coefficients of them between the two halves. The distribution of map correlations between independent HCP split halves for task-specific SDAs (**c**) and task-general SDAs (**d**) establish moderate-strong reproducibility of the topography of SDAs in the human brain. Source data are provided as Source Data files.

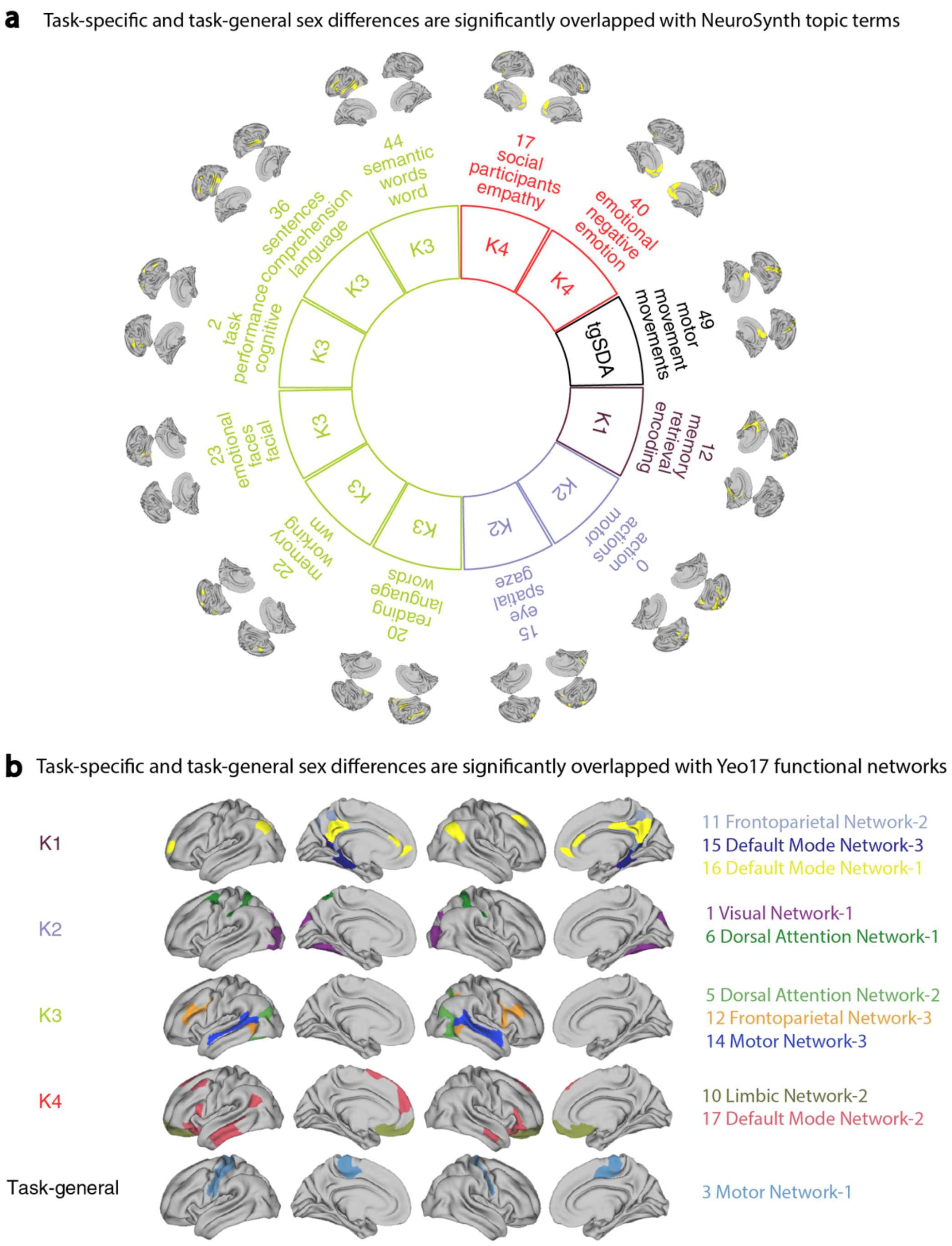

Supplementary Fig. 6: Brain regions exhibiting significant sex differences in activation are enriched within canonical functional systems and networks. (**a**) Conjunction maps (surface projections in the outer circle) for each of the metanalytic Neurosynth "topic terms" (texts in the middle circle) that showed regional activations which significantly overlapped with each cluster of task-specific sex differences in activation (task-specific SDA) in (**a**) and with regions exhibiting task-general sex differences in activation (task-general SDA) in (**b**) (labeled in the inner circle) based on Spin Tests with 10k spatial permutations and additional Bonferroni correction for 24 topic terms ($P_{spin} < 0.05/24$, **Methods**). NeuroSynth topics that

significantly overlap with each cluster based on Spin Tests are referred to by the number and topic terms (https://neurosynth.org/analyses/topics/v4-topics-50/). Colored middle and inner circles match the cluster colors from (**Fig. 1b**). (**b**) Conjunction maps of the 4 task-specific SDA clusters (colors of K1-K4 match the cluster colors from **Fig. 1b**) and the 1 set of task-general SDA regions with those Yeo-Krienen resting state connectivity cortical networks[6] that showed statistically significant overlaps with SDAs based on 10k Spin Test permutations and Bonferroni correction of resulting empirical p values for multiple comparisons across 17 networks of the Yeo-Krienen parcellation (P_{spin}<0.05/17).

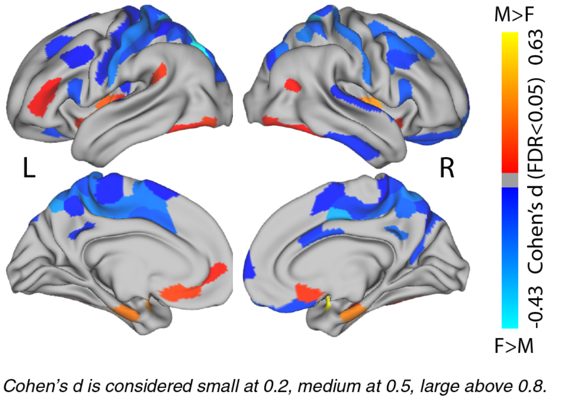

Supplementary Fig. 7: Effect sizes of statistically significant sex differences in brain volume (SDVs).

Cohen's d showing weak to moderate effect sizes (up to 0.63) of those statistically significant sex differences in gray matter volumes (SDVs) shown in **Fig. 2a**.

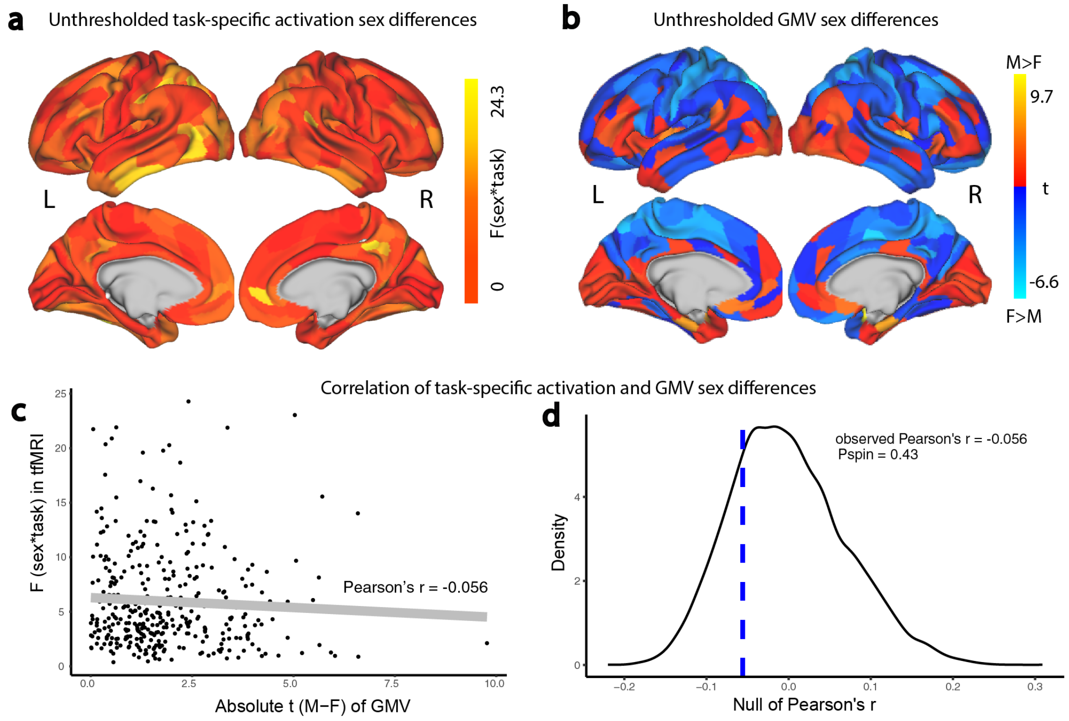

Supplementary Fig. 8: The spatial dissociation between task-specific sex differences in brain activation (task-specific SDAs) and sex differences in brain volume (SDVs).(**a, b**) Surface projection showing the unthresholded test statistics for task-specific SDAs (F values from β3 coefficient in Model 1, **a**) and SDVs (T values from β1 coefficient in Model 4, **b**) for all 360 regions of the HCP cortical parcellation (**c**) Scatter plot showing a low across-region correlation coefficient between these two maps (Pearson's r = -0.056). (**d**) Based on 10k spin tests, the observed correlation was not elevated above chance (P_{spin} = 0.43) — indicating a lack of spatial coupling between task-specific SDAs and SDVs in the human brain. Source data are provided as Source Data files.

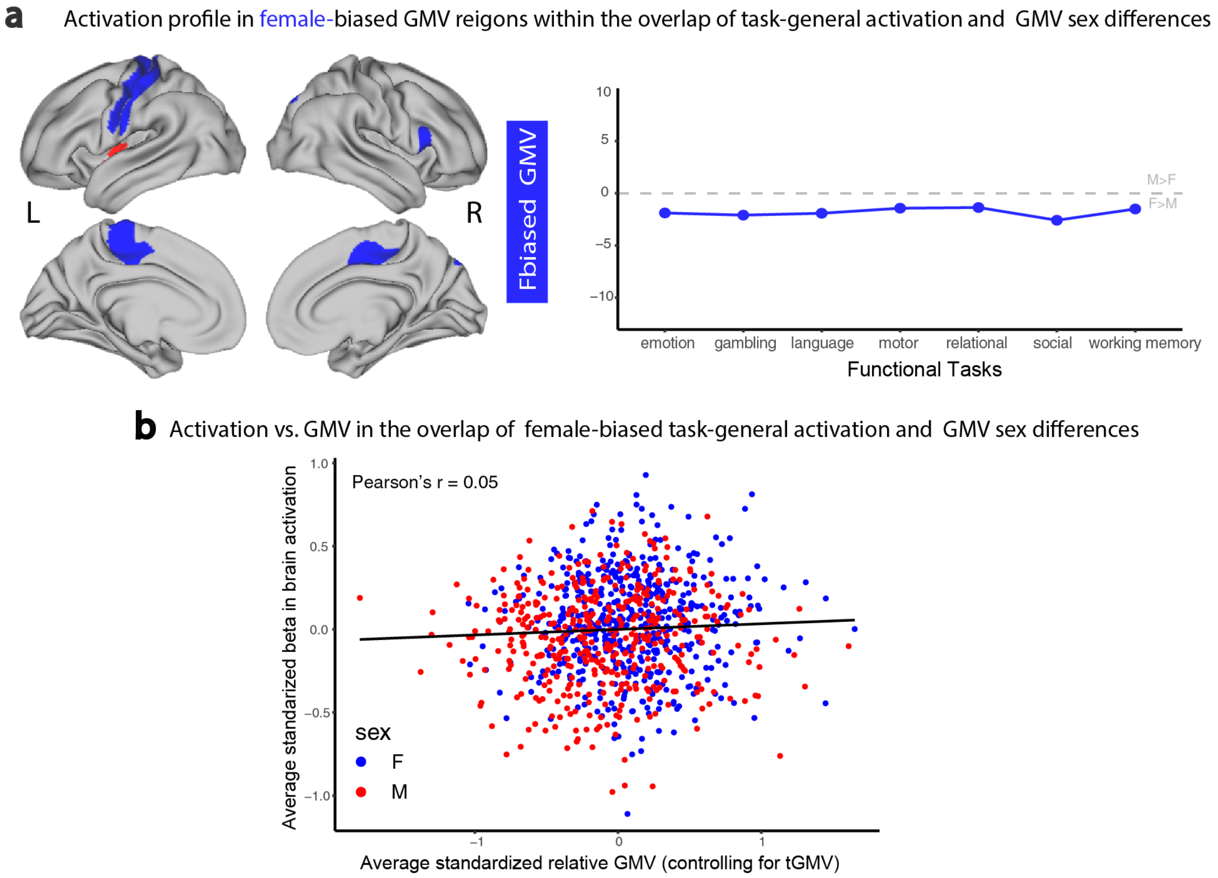

Supplementary Fig. 9: Regions of overlapping task-general sex differences in brain activation (task-general SDAs) and sex differences in volume (SDVs) are both female-biased, but activation and volume of these regions are not inter-correlated across individuals (**a**) Colored regions show both statistically significant task-general SDAs and SDVs (i.e., green regions in **Fig. 2d**). Color denotes the direction of SDV (blue: female>male; red: male>female) — indicating almost all regions show female-biased volume. These regions with female-biased volume also show a tendency towards female-biased activation across all seven HCP tasks as shown in the point-line (plot to right of cortical map). (**b**) Despite the significant spatial overlap between task-general SDAs and SDVs (**Fig. 2d**), with a concordant female-bias in regional volume and activation in the overlap region (blue in **a**), it is not the case that individuals with lesser volume in these regions show lesser activation (r=0.05, **b**). Source data are provided as Source Data files.

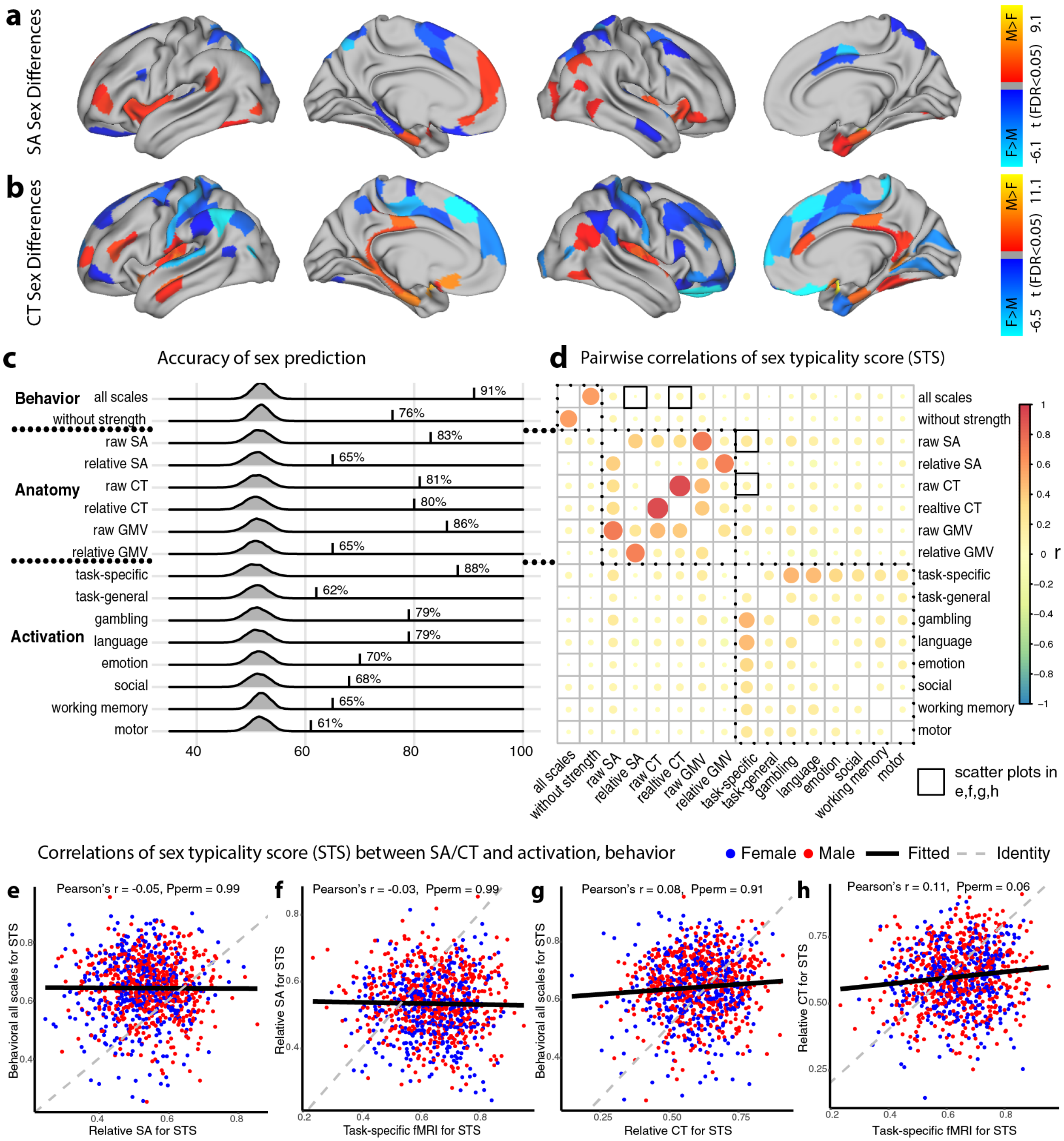

Supplementary Fig. 10: Sex typicality of brain anatomy remains uncorrelated with that for brain activation and behavior when considering two non-volumetric measures of cortical anatomy — surface area (SA) and cortical thickness (CT) (**a**, **b**) By replacing volume with surface area (SA) and cortical thickness (CT) in Model 4, we identified statistically significant sex differences in regional cortical SA (**a**) and CT (**b**) after correction for multiple comparisons across brain regions (FDR q < 0.05), while controlling for total SA and mean CT. (**c**) Sex prediction accuracy was evaluated using Partial Least Squares Discriminant Analysis (PLSDA) with 10-fold cross-validation across 16 distinct feature sets — comprising behavioral, structural MRI, and fMRI

data — following the addition of 4 SA and CT-based measures to the original 12 shown in **Fig. 3b** (**Methods**): *relative SA* controlling for total SA and *raw SA* without controlling for total SA in regions showing statistically significant sex differences in regional SA (**a**); *relative CT* controlling for average CT and *raw CT* without controlling for average CT in regions showing statistically significant sex differences in regional CT (**b**). The achieved accuracy of sex prediction for each feature set (bar) is shown relative to a null distribution of accuracies from 10k permutations of sex. (**d**) Dot plot showing correlations between person-level sex typicality scores (STSs) for each of these 16 feature sets used in PLSDA (the original 12 shown in **Fig. 3c**, the complete values of all 16 shown in **Supplementary Data 8**). Each person's STS of a feature set is the probability assigned to that person for membership of their true sex group based on PLSDA of that feature set. Dot size and color encode correlations. The four cells with black border denote correlations for which scatterplots are provided in **e**, **f**, **g**, **h** for SA and CT-based measures. (**e**,**f**,**g**,**h**) Scatter plots for pairwise correlations between selected STS scores, which remain weak ($r<0.11$) and not significant ($P_{perm}>0.06$): STS from relative regional SA vs. STS from all behavioral scales (**e**); STS from task-specific activation vs. STS from relative regional SA (**f**); STS from relative regional CT vs. STS from all behavioral scales (**g**); STS from task-specific activation vs. STS from relative regional CT. Source data are provided as Source Data files.

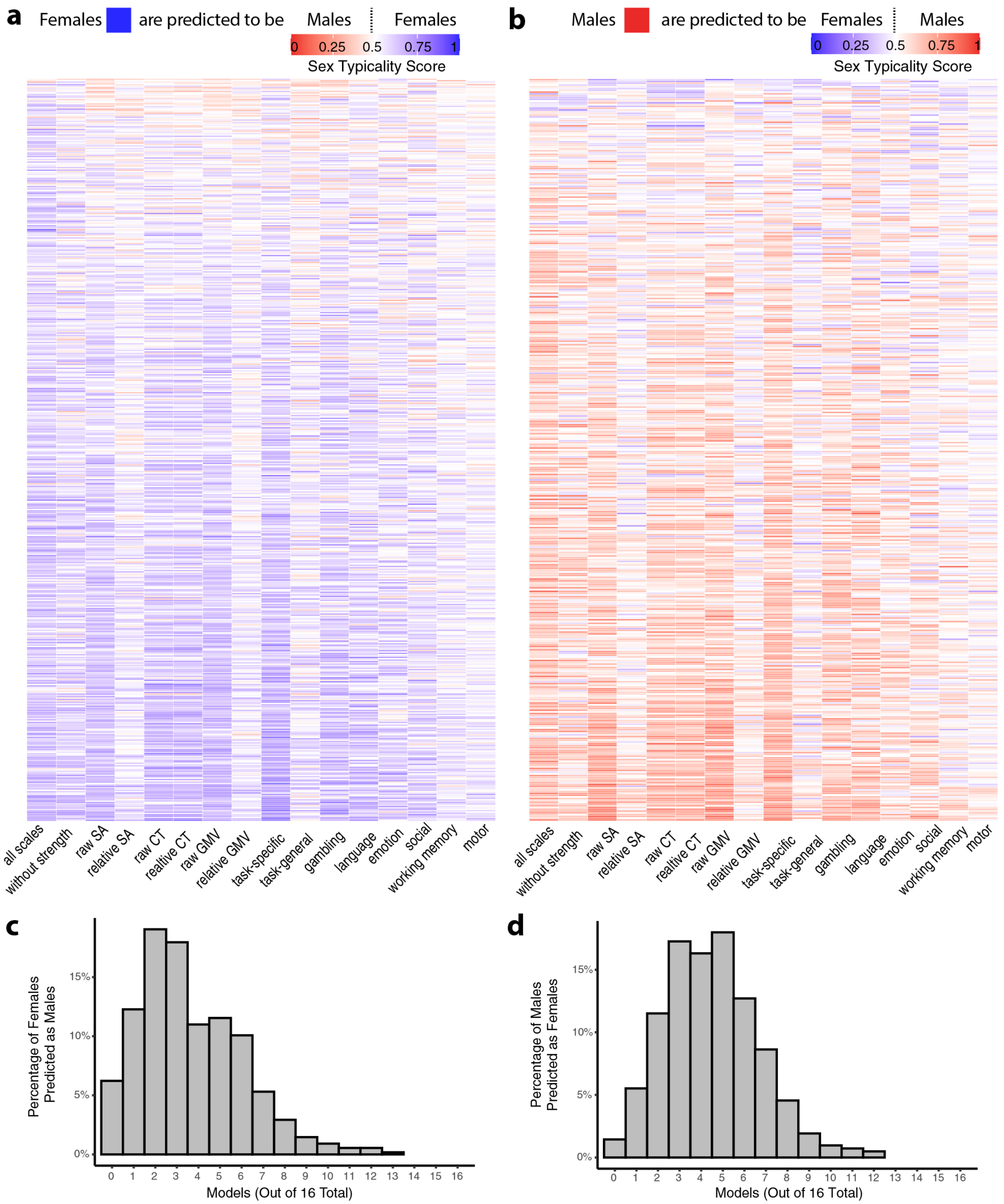

Supplementary Fig. 11: Person-level sex typicality scores and percentage of individuals whose sex was misclassified across 16 sex prediction models (**a**,**b**) Sorted tile plots of sex typicality scores (STSs) for each individual (rows) across 16 sex prediction models (columns, as defined in **Supplementary Fig. 10c**), ordered by average sex typicality scores in ascending order for females (**a**) and males (**b**). By definition, individuals with a sex typicality score above 0.5 are

classified as their actual sex—females as female and males as male—while those with scores below 0.5 are misclassified. (**c,d**) Histograms showing the proportion of individuals as a function of the number of models (0–16) in which their sex was misclassified, separately for females (**c**) and males (**d**). For example, the bin at x = 0 in panel **c** represents the percentage of females who were misclassified in none of the 16 models—that is, females who were correctly classified by all models. Using this criterion, only 6.2% of females (panel **c**) and 1.4% of males (panel **d**) were correctly classified across all 16 models, corresponding to the leftmost bins in each panel.

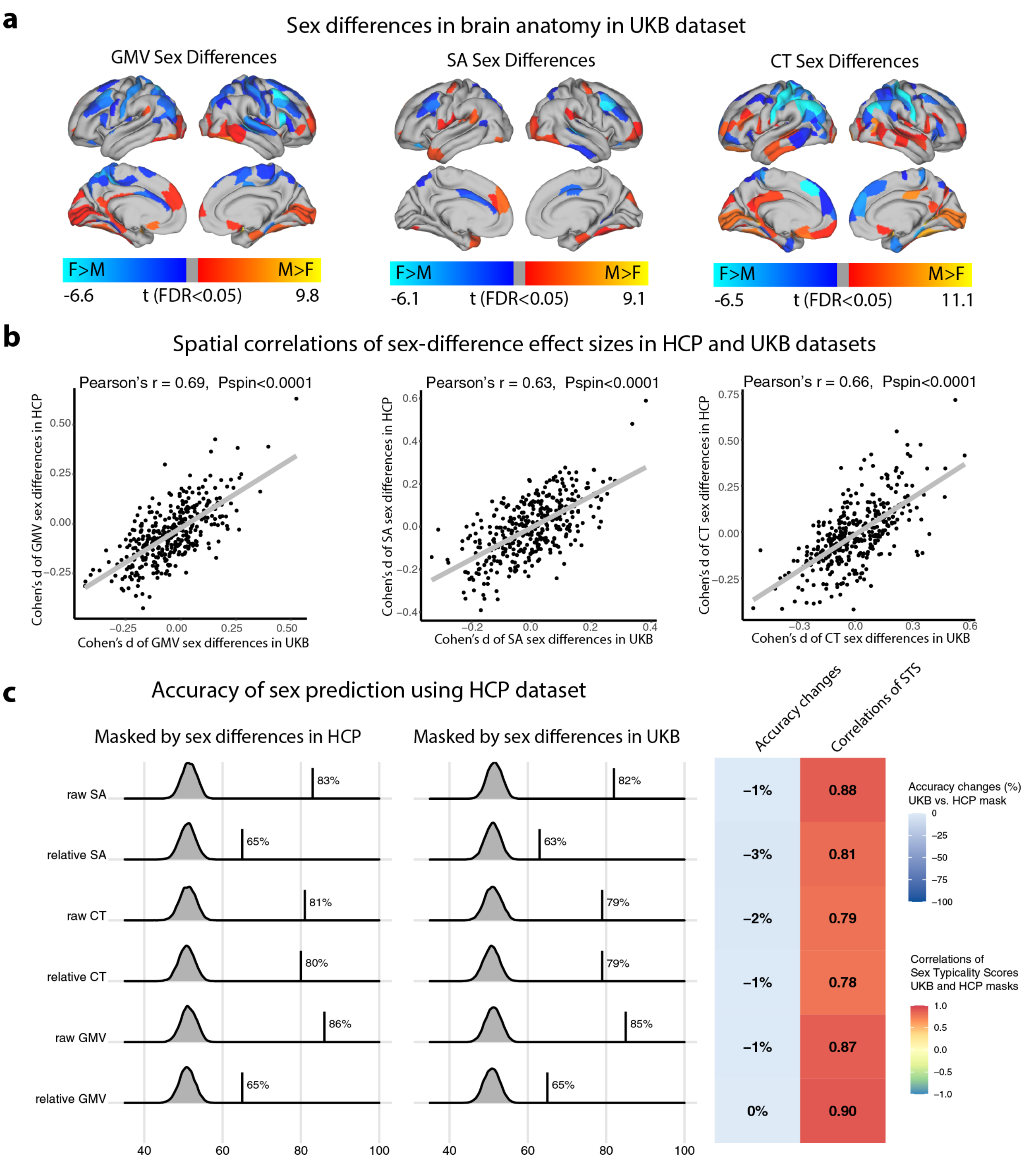

Supplementary Fig. 12: Cross-validation of sex differences and sex prediction performance in brain anatomy using an independent UK Biobank dataset. **(a)** We repeated the structural MRI analyses applied to the primary HCP dataset in an independent UK Biobank (UKB) sample and identified statistically significant sex differences in regional gray matter volume (GMV), surface area (SA), and cortical thickness (CT). Color maps show *t* values for regional sex differences after controlling for corresponding global measures (total GMV, total

SA, and mean CT) and correcting for multiple comparisons across brain regions (FDR q < 0.05). **(b)** Scatter plots showing strong spatial correspondence between structural sex differences derived from the UKB (x-axis) and HCP (y-axis) datasets. Each point represents Cohen's *d* for one of 360 brain regions defined by the HCP parcellation. Effect sizes were highly correlated (r > 0.63) and significantly exceeded chance levels based on 10,000 spin permutations (Pspin<0.0001). **(c)** Effects of using different datasets to define the mask of significant sex differences for sex prediction. In the left column, we reproduced the original sex prediction accuracy for each anatomical feature set (bars; see **Supplementary Fig. 10c**), where the HCP dataset was used for both mask definition and prediction, relative to a null distribution generated from 10,000 sex-label permutations. In the right column, masks of significant sex differences derived from the UKB dataset (panel **a**) were used as model inputs, and sex prediction was performed in the HCP dataset. The first color bar indicates changes in prediction accuracy between the two approaches, showing only a modest reduction (<3%) when using the independent UKB dataset for masking. The second color bar shows high correlations (r > 0.78) between sex typicality scores (STSs) across participants obtained from the two modeling approaches, indicating that shared interindividual information was used for sex prediction in both cases. Source data are provided as Source Data files.

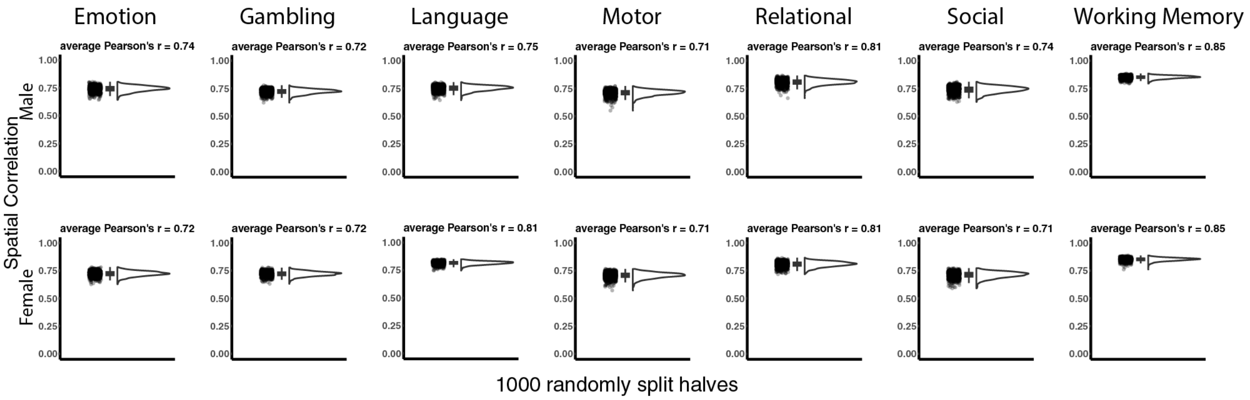

Supplementary Fig. 13: Reproducibility of brain-wide association study (BWAS) signal between independent split halves of the HCP dataset in males and females. Using 1k split-half tests, in each of which we randomly split the HCP male and female samples separately into halves, we repeated the BWAS calculation to identify t values of activation-behavior associations in each half and calculated Pearson's correlation coefficients of them between the two halves across regions and behaviors where nominally significant activation-behavior associations were observed in the full samples shown in **Fig. 4a**. For each task, the distributions of correlations between independent male (top row) and female (bottom row) split halves establish strong reproducibility for BWAS signal identified in each sex (**Fig. 4a)**. Note that here we included the motor task in males where the global BWAS signal failed to reach the Bonferroni-corrected significance (**Fig. 4a**) because there are still pairs of regions and behaviors

in this matrix showing nominally significant activation-behavior associations. Source data are provided as Source Data files.

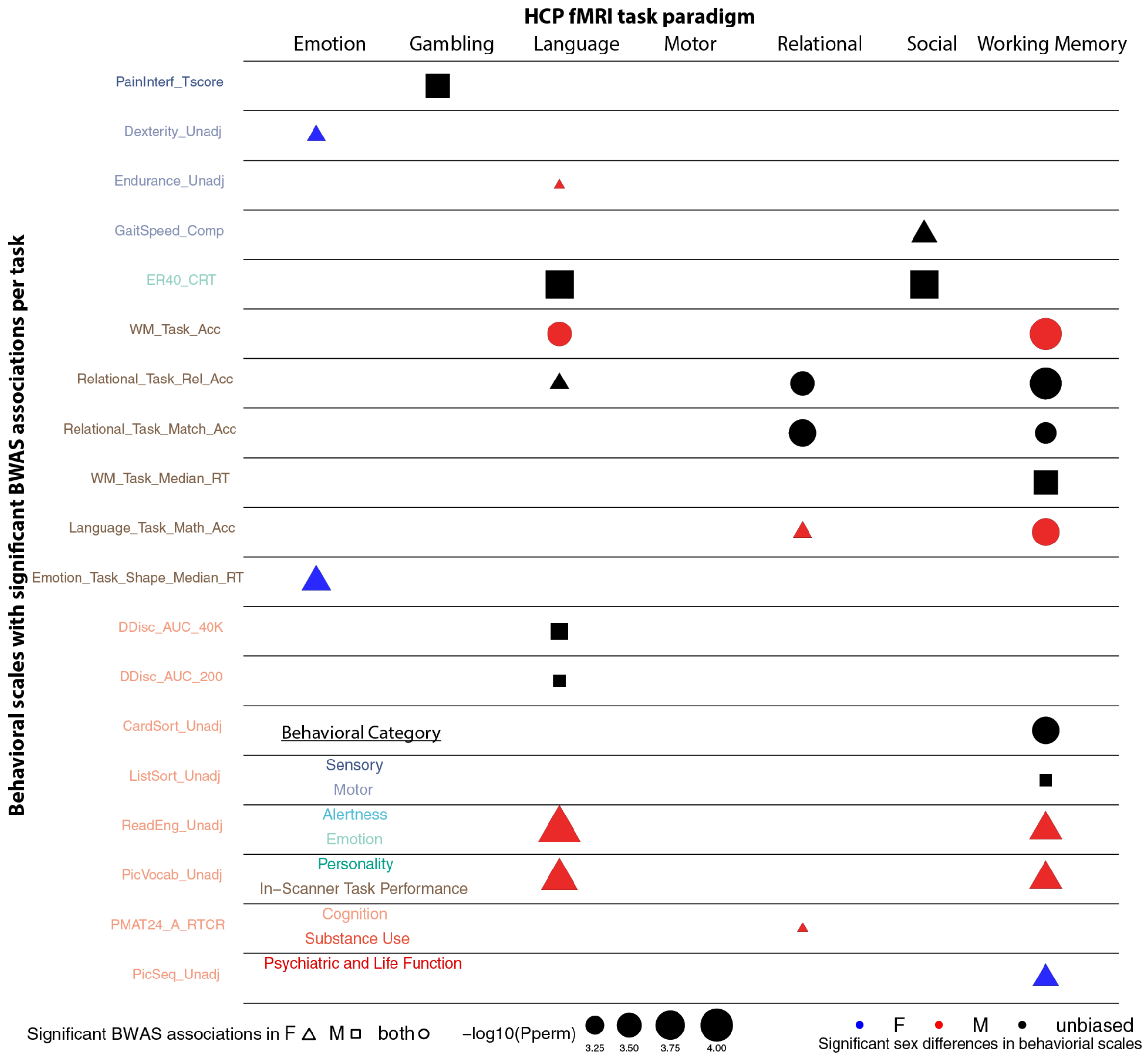

Supplementary Fig. 14: Dot plot showing the specific behavioral scales that show an enriched brain-wide association study (BWAS) signal in each of the seven HCP fMRI tasks except motor. Each row relates to one of the 19 behavioral scales that showed a significantly enriched BWAS signal in at least one sex for one of the seven HCP fMRI tasks (each column relates to one of the seven tasks). Row text color denotes the overarching behavioral and cognitive category in which each behavioral scale lies (see inset color key). Dot size denotes the statistical significance of this BWAS signal. Dot shape shows if the BWAS enrichment was seen in females only (triangle), males only (square) or both sexes (circle). Dot color shows if the behavioral scale shows a statistically significant difference in its mean value between sexes. Note that there is not a consistent congruence between the cognitive domain tapped by a given fMRI task and those behavioral and cognitive scales that those enriched BWAS signal for that task. Although this congruence is present in several instances (e.g. in-scanner response time in emotion

task and activation in fMRI emotion task; scores in measures of language functioning and activation in fMRI language task), we also observe numerous instances of discordance (e.g. behavioral measures of dexterity and activation in fMRI emotion task; measure of pain sensitivity and activation in fMRI gambling task; gait speed and activation in fMRI social task). Moreover, these significant sex-specific activation-behavior associations are not enriched in behaviors that show significant sex differences in mean values as P values of Per task Fisher's tests for associations between significant status of activation-behavior (dictated by shape: not significant, significant in males, in females, in both), and of sex differences in mean values (dictated by dot color: not significant, significant in males, in females) across behavioral scales are greater than 0.06 across tasks. Source data are provided as Source Data files.

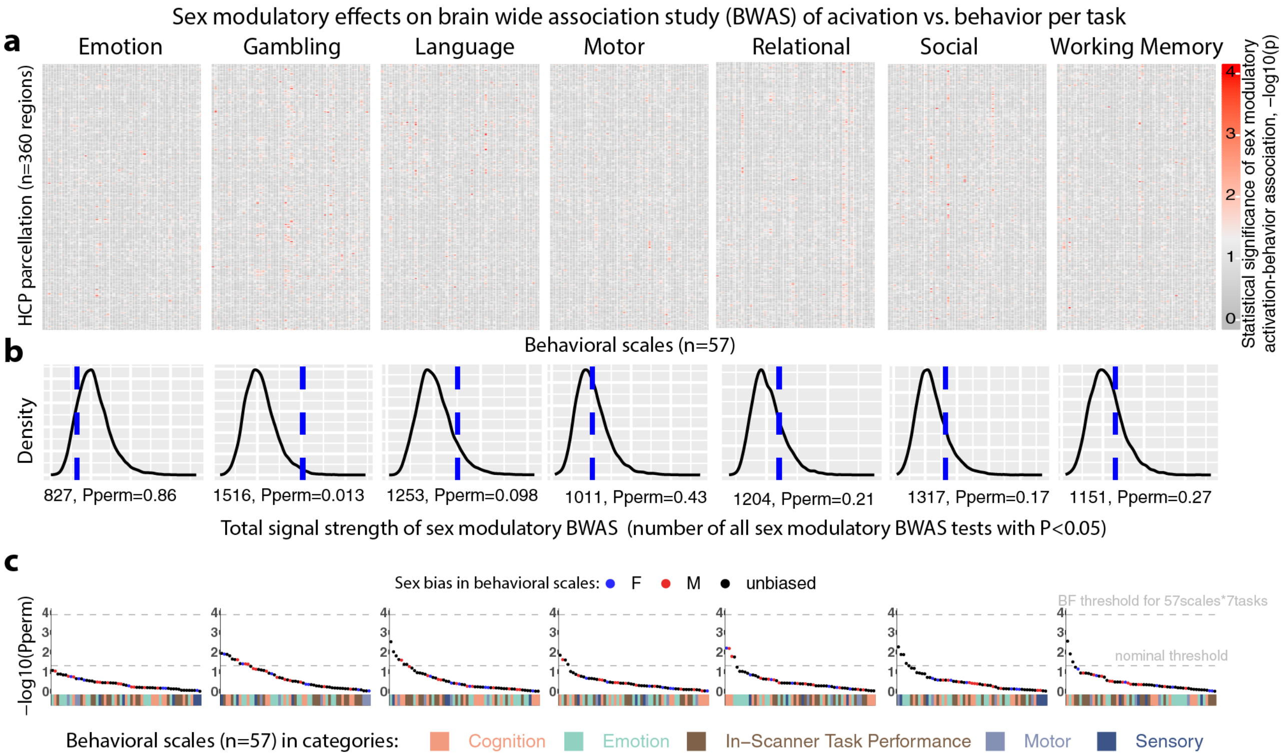

Supplementary Fig. 15: Sex modulatory effects on brain-wide association study (BWAS) results of interindividual activation-behavior associations.

(**a**) We implemented sex modulatory brain-wide association study (BWAS) of activation vs. behavior (i.e., behavior ~ sex*activation) across participants per task. Color in each cell in the heat map specifies the statistical significance [-log10(P)] of sex modulatory activation-behavior association tests in all pairs (360 brain regions in rows by 57 behavioral scales in columns). (**b**) In all seven tasks, the total signal strength of BWAS, measured by the sum of sex modulatory BWAS association tests with normal significance (P<0.05), was not statically significantly (Bonferroni corrected for the number of tasks, $P_{perm}<0.05/7$) higher than the null generated by 10k sex shuffling. And we uncovered the highest sex modulator effect in the gambling task ($P_{perm}=0.013$).

(**c**) Dot plots rank the 57 behaviors (shown in x-axis with colors matching **Fig. 3a** to denote behavioral category) by descending sex modulation effects on activation-behavior associations for each task (greater -log10(P_{perm}) indicting greater sex modulatory effects on BWAS maps of activation vs. behavior, **Methods**, *Sex modulatory effects in BWAS*). Dot color indicates whether the mean behavioral score is female-biased (blue), male-biased (red) or neither (black). No behaviors showed sex modulatory effects that were significantly elevated above zero after Bonferroni correction for multiple comparisons across all 57 behavioral scales and 7 fMRI tasks ($P_{perm}<0.05/399$), although 33 out of 399 behavior-task combinations (source data are provided in **Supplementary Data 11**) showed nominally significant sex modulatory effects on activation-behavior associations ($P_{perm}<0.05$).

References

1. Alexander-Bloch, A. F. *et al.* On testing for spatial correspondence between maps of human brain structure and function. *Neuroimage* **178**, 540–551 (2018).
2. Markello, R. D. & Misic, B. Comparing spatial null models for brain maps. *Neuroimage* **236**, 118052 (2021).
3. Yarkoni, T., Poldrack, R. A., Nichols, T. E., Van Essen, D. C. & Wager, T. D. Large-scale automated synthesis of human functional neuroimaging data. *Nat. Methods* **8**, 665–670 (2011).
4. Poldrack, R. A. *et al.* Discovering relations between mind, brain, and mental disorders using topic mapping. *PLoS Comput. Biol.* **8**, e1002707 (2012).
5. Mallard, T. T. *et al.* X-chromosome influences on neuroanatomical variation in humans. *Nat. Neurosci.* **24**, 1216–1224 (2021).
6. Yeo, B. T. T. *et al.* The organization of the human cerebral cortex estimated by intrinsic functional connectivity. *J. Neurophysiol.* **106**, 1125–1165 (2011).

7. Van Essen, D. C. *et al.* The Human Connectome Project: a data acquisition perspective. *Neuroimage* **62**, 2222–2231 (2012).

8. Barch, D. M. *et al.* Function in the human connectome: task-fMRI and individual differences in behavior. *Neuroimage* **80**, 169–189 (2013).

9. Drobyshevsky, A., Baumann, S. B. & Schneider, W. A rapid fMRI task battery for mapping of visual, motor, cognitive, and emotional function. *Neuroimage* **31**, 732–744 (2006).

10. Binder, J. R. *et al.* Mapping anterior temporal lobe language areas with fMRI: a multicenter normative study. *Neuroimage* **54**, 1465–1475 (2011).

11. Miller, M. B. *et al.* Unique and persistent individual patterns of brain activity across different memory retrieval tasks. *Neuroimage* **48**, 625–635 (2009).

12. Smith, R., Keramatian, K. & Christoff, K. Localizing the rostrolateral prefrontal cortex at the individual level. *Neuroimage* **36**, 1387–1396 (2007).

13. May, J. C. *et al.* Event-related functional magnetic resonance imaging of reward-related brain circuitry in children and adolescents. *Biol. Psychiatry* **55**, 359–366 (2004).
