# Supplementary material for: Robust but independent sex differences in human brain function, structure, and behavior
Source: Nat Commun. 2026 May 21;17:6694. doi: 10.1038/s41467-026-73262-2 (PMC13385741; doi:10.1038/s41467-026-73262-2)
Supplement: Supplementary file 2 — Description of Additional Supplementary Files [file 41467_2026_73262_MOESM2_ESM.pdf]

Description of Additional Supplementary Files

File Name: Supplementary Data 1
Description: Regional distribution of main effects in individual fMRI tasks.

File Name: Supplementary Data 2
Description: Regional distribution of task-specific sex differences in activation and K-mean clustering results.

File Name: Supplementary Data 3
Description: Regional distribution of sex differences in activation in individual fMRI tasks.

File Name: Supplementary Data 4
Description: ROI-based task-specific and task-general sex differences in activation in individual fMRI tasks.

File Name: Supplementary Data 5
Description: Regional distribution of task-general sex differences in activation.

File Name: Supplementary Data 6
Description: Conjunctions between regions of significant Sex Differences in Activation and Sex Differences in Volume.

File Name: Supplementary Data 7
Description: Sex differences in behavioral scales.

File Name: Supplementary Data 8
Description: Pairwise comparisons of Sex-Typicality Scores derived from sex prediction models

File Name: Supplementary Data 9
Description: Associations of brain activation and behavioral scores in males and females.

File Name: Supplementary Data 10
Description: Sorted between sex topographical divergence scores across behavioral scales per task.

File Name: Supplementary Data 11
Description: Sorted sex modulatory effects on activation-behavior associations across behavioral scales per task.
