# Supplementary material for: Robust but independent sex differences in human brain function, structure, and behavior
Source: Nat Commun. 2026 May 21;17:6694. doi: 10.1038/s41467-026-73262-2 (PMC13385741; doi:10.1038/s41467-026-73262-2)
Supplement: Supplementary file 14 — Reporting Summary [file 41467_2026_73262_MOESM14_ESM.pdf]

nature portfolio

Corresponding author(s): Armin Raznahan

Last updated by author(s): Feb 13, 2026

Reporting Summary

Nature Portfolio wishes to improve the reproducibility of the work that we publish. This form provides structure for consistency and transparency in reporting. For further information on Nature Portfolio policies, see our Editorial Policies and the Editorial Policy Checklist.

Statistics

For all statistical analyses, confirm that the following items are present in the figure legend, table legend, main text, or Methods section.

n/a	Confirmed	
☐	☒	The exact sample size (n) for each experimental group/condition, given as a discrete number and unit of measurement
☐	☒	A statement on whether measurements were taken from distinct samples or whether the same sample was measured repeatedly
☐	☒	The statistical test(s) used AND whether they are one- or two-sided<br>*Only common tests should be described solely by name; describe more complex techniques in the Methods section.*
☐	☒	A description of all covariates tested
☐	☒	A description of any assumptions or corrections, such as tests of normality and adjustment for multiple comparisons
☐	☒	A full description of the statistical parameters including central tendency (e.g. means) or other basic estimates (e.g. regression coefficient) AND variation (e.g. standard deviation) or associated estimates of uncertainty (e.g. confidence intervals)
☐	☒	For null hypothesis testing, the test statistic (e.g. F, t, r) with confidence intervals, effect sizes, degrees of freedom and P value noted<br>*Give P values as exact values whenever suitable.*
☒	☐	For Bayesian analysis, information on the choice of priors and Markov chain Monte Carlo settings
☒	☐	For hierarchical and complex designs, identification of the appropriate level for tests and full reporting of outcomes
☐	☒	Estimates of effect sizes (e.g. Cohen's d, Pearson's r), indicating how they were calculated

Our web collection on statistics for biologists contains articles on many of the points above.

Software and code

Policy information about availability of computer code

Data collection	No software was used for data collection.
Data analysis	Details of the data analyses, including primary software packages and mathematical models, are provided in the Methods section. All code is available at https://github.com/siyuanliu8/HCPsexdifferences.

For manuscripts utilizing custom algorithms or software that are central to the research but not yet described in published literature, software must be made available to editors and reviewers. We strongly encourage code deposition in a community repository (e.g. GitHub). See the Nature Portfolio guidelines for submitting code & software for further information.

Data

Policy information about availability of data

All manuscripts must include a data availability statement. This statement should provide the following information, where applicable:

- Accession codes, unique identifiers, or web links for publicly available datasets
- A description of any restrictions on data availability
- For clinical datasets or third party data, please ensure that the statement adheres to our policy

The primary Human Connectome Project (HCP) data are available by application at: https://www.humanconnectome.org/study/hcp-young-adult. The UK Biobank (UKB) validation data can be accessed by submitting an application at: https://www.ukbiobank.ac.uk/enable-your-research/apply-for-access.

Research involving human participants, their data, or biological material

Policy information about studies with human participants or human data. See also policy information about sex, gender (identity/presentation), and sexual orientation and race, ethnicity and racism.

Reporting on sex and gender	Sex was self-reported in the HCP and UKB dataset and incorporated into the study design, as our primary objective was to examine normative sex differences in human brain function, structure, and behavior. Disaggregated information on sex and gender was not collected in the HCP dataset.
Reporting on race, ethnicity, or other socially relevant groupings	Healthy participants in the HCP dataset were recruited to broadly reflect the ethnic and racial composition of the U.S. population. Because our primary objective was to investigate sex differences, and no significant associations were observed between sex and race/ethnicity ($P > 0.05$, chi-square tests), race and ethnicity were not included as covariates in the analyses.
Population characteristics	The final primary HCP cohort included 455 male participants (mean age 27.8 ± 3.7 years) and 523 female participants (mean age 29.5 ± 3.6 years). Although the groups differed slightly in mean age ($P < 0.05$, two-sample t-test), all participants were within the same age range (22–37 years). The youngest subset of healthy UK Biobank participants (44–50 years; 529 males and 637 females) who were scanned at a single site (site #11025) were used for validation. Age was included as a covariate in the models to assess sex differences in both HCP and UKB data.
Recruitment	The primary HCP participant pool consisted of healthy individuals born in Missouri. Detailed recruitment procedures are described in The WU-Minn Human Connectome Project: An Overview (https://pmc.ncbi.nlm.nih.gov/articles/PMC3724347/).The UK Biobank is a large population-based study that is largely representative of the UK population. Recruitment details can be found in https://pubmed.ncbi.nlm.nih.gov/22463865/.
Ethics oversight	Analyses of the HCP and UKB data and the research protocol were approved by the institutional review board at the National Institute of Mental Health.

Note that full information on the approval of the study protocol must also be provided in the manuscript.

Field-specific reporting

Please select the one below that is the best fit for your research. If you are not sure, read the appropriate sections before making your selection.

☒ Life sciences ☐ Behavioural & social sciences ☐ Ecological, evolutionary & environmental sciences

For a reference copy of the document with all sections, see nature.com/documents/nr-reporting-summary-flat.pdf

Life sciences study design

All studies must disclose on these points even when the disclosure is negative.

Sample size	Sample size was determined by the number of participants included in the HCP S1200 data release (March 2017).
Data exclusions	For analyses of sex differences in brain structure, we excluded 15 participants (yielding 444 males and 519 females) with an Euler number below –217, a conservative threshold recommended by Rosen et al. (2018) to ensure high-quality segmentation and registration.
Replication	The HCP dataset is globally unique, comprising over 700 hours of fMRI data from seven tasks spanning diverse domains of brain function, along with structural brain measures and more than 85 behavioral scales in nearly 1,000 individuals. Consequently, full replication with an independent dataset is not feasible. To address reproducibility, we implemented a split-half approach within the HCP dataset, as detailed in the manuscript. We also conducted a partial replication of our findings on brain structure using a subset of the publicly available UK Biobank dataset (www.ukbiobank.ac.uk).
Randomization	Participants were grouped according to sex; randomization was not applicable.
Blinding	Participants were grouped by sex; blinding was neither feasible nor relevant to the study design.

Reporting for specific materials, systems and methods

We require information from authors about some types of materials, experimental systems and methods used in many studies. Here, indicate whether each material, system or method listed is relevant to your study. If you are not sure if a list item applies to your research, read the appropriate section before selecting a response.

Materials & experimental systems

n/a	Involved in the study
[ ]	[ ] Antibodies
[ ]	[ ] Eukaryotic cell lines
[ ]	[ ] Palaeontology and archaeology
[ ]	[ ] Animals and other organisms
[ ]	[x] Clinical data
[ ]	[ ] Dual use research of concern
[ ]	[ ] Plants

Methods

n/a	Involved in the study
[ ]	[ ] ChIP-seq
[ ]	[ ] Flow cytometry
[ ]	[x] MRI-based neuroimaging

Antibodies

Antibodies used

Describe all antibodies used in the study; as applicable, provide supplier name, catalog number, clone name, and lot number.

Validation

Describe the validation of each primary antibody for the species and application, noting any validation statements on the manufacturer's website, relevant citations, antibody profiles in online databases, or data provided in the manuscript.

Eukaryotic cell lines

Policy information about cell lines and Sex and Gender in Research

Cell line source(s)

State the source of each cell line used and the sex of all primary cell lines and cells derived from human participants or vertebrate models.

Authentication

Describe the authentication procedures for each cell line used OR declare that none of the cell lines used were authenticated.

Mycoplasma contamination

Confirm that all cell lines tested negative for mycoplasma contamination OR describe the results of the testing for mycoplasma contamination OR declare that the cell lines were not tested for mycoplasma contamination.

Commonly misidentified lines
(See ICLAC register)

Name any commonly misidentified cell lines used in the study and provide a rationale for their use.

Palaeontology and Archaeology

Specimen provenance

Provide provenance information for specimens and describe permits that were obtained for the work (including the name of the issuing authority, the date of issue, and any identifying information). Permits should encompass collection and, where applicable, export.

Specimen deposition

Indicate where the specimens have been deposited to permit free access by other researchers.

Dating methods

If new dates are provided, describe how they were obtained (e.g. collection, storage, sample pretreatment and measurement), where they were obtained (i.e. lab name), the calibration program and the protocol for quality assurance OR state that no new dates are provided.

[ ] Tick this box to confirm that the raw and calibrated dates are available in the paper or in Supplementary Information.

Ethics oversight

Identify the organization(s) that approved or provided guidance on the study protocol, OR state that no ethical approval or guidance was required and explain why not.

Note that full information on the approval of the study protocol must also be provided in the manuscript.

Animals and other research organisms

Policy information about studies involving animals; ARRIVE guidelines recommended for reporting animal research, and Sex and Gender in Research

Laboratory animals

For laboratory animals, report species, strain and age OR state that the study did not involve laboratory animals.

Wild animals

Provide details on animals observed in or captured in the field; report species and age where possible. Describe how animals were caught and transported and what happened to captive animals after the study (if killed, explain why and describe method; if released, say where and when) OR state that the study did not involve wild animals.

Reporting on sex

Indicate if findings apply to only one sex; describe whether sex was considered in study design, methods used for assigning sex. Provide data disaggregated for sex where this information has been collected in the source data as appropriate; provide overall numbers in this Reporting Summary. Please state if this information has not been collected. Report sex-based analyses where performed, justify reasons for lack of sex-based analysis.

Field-collected samples	*For laboratory work with field-collected samples, describe all relevant parameters such as housing, maintenance, temperature, photoperiod and end-of-experiment protocol OR state that the study did not involve samples collected from the field.*
Ethics oversight	*Identify the organization(s) that approved or provided guidance on the study protocol, OR state that no ethical approval or guidance was required and explain why not.*

Note that full information on the approval of the study protocol must also be provided in the manuscript.

Clinical data

Policy information about clinical studies

All manuscripts should comply with the ICMJE guidelines for publication of clinical research and a completed CONSORT checklist must be included with all submissions.

Clinical trial registration	NCT00001246
Study protocol	The HCP data re deiidentified and their usage do not fall under IRB review
Data collection	Data collection procedures are described in The WU-Minn Human Connectome Project: An Overview (https://pmc.ncbi.nlm.nih.gov/articles/PMC3724347/).
Outcomes	We evaluated sex differences in brain activation, cortical volume, and behavior primarily using mixed-effects and regression models.

Dual use research of concern

Policy information about dual use research of concern

Hazards

Could the accidental, deliberate or reckless misuse of agents or technologies generated in the work, or the application of information presented in the manuscript, pose a threat to:

No	Yes	
x		Public health
x		National security
x		Crops and/or livestock
x		Ecosystems
x		Any other significant area

Experiments of concern

Does the work involve any of these experiments of concern:

No	Yes	
x		Demonstrate how to render a vaccine ineffective
x		Confer resistance to therapeutically useful antibiotics or antiviral agents
x		Enhance the virulence of a pathogen or render a nonpathogen virulent
x		Increase transmissibility of a pathogen
x		Alter the host range of a pathogen
x		Enable evasion of diagnostic/detection modalities
x		Enable the weaponization of a biological agent or toxin
		Any other potentially harmful combination of experiments and agents

Plants

Seed stocks

Report on the source of all seed stocks or other plant material used. If applicable, state the seed stock centre and catalogue number. If plant specimens were collected from the field, describe the collection location, date and sampling procedures.

Novel plant genotypes

Describe the methods by which all novel plant genotypes were produced. This includes those generated by transgenic approaches, gene editing, chemical/radiation-based mutagenesis and hybridization. For transgenic lines, describe the transformation method, the number of independent lines analyzed and the generation upon which experiments were performed. For gene-edited lines, describe the editor used, the endogenous sequence targeted for editing, the targeting guide RNA sequence (if applicable) and how the editor was applied.

Authentication

Describe any authentication procedures for each seed stock used or novel genotype generated. Describe any experiments used to assess the effect of a mutation and, where applicable, how potential secondary effects (e.g. second site T-DNA insertions, mosiacism, off-target gene editing) were examined.

ChIP-seq

Data deposition

- [ ] Confirm that both raw and final processed data have been deposited in a public database such as GEO.
- [ ] Confirm that you have deposited or provided access to graph files (e.g. BED files) for the called peaks.

Data access links
May remain private before publication.

For "Initial submission" or "Revised version" documents, provide reviewer access links. For your "Final submission" document, provide a link to the deposited data.

Files in database submission

Provide a list of all files available in the database submission.

Genome browser session
(e.g. UCSC)

Provide a link to an anonymized genome browser session for "Initial submission" and "Revised version" documents only, to enable peer review. Write "no longer applicable" for "Final submission" documents.

Methodology

Replicates

Describe the experimental replicates, specifying number, type and replicate agreement.

Sequencing depth

Describe the sequencing depth for each experiment, providing the total number of reads, uniquely mapped reads, length of reads and whether they were paired- or single-end.

Antibodies

Describe the antibodies used for the ChIP-seq experiments; as applicable, provide supplier name, catalog number, clone name, and lot number.

Peak calling parameters

Specify the command line program and parameters used for read mapping and peak calling, including the ChIP, control and index files used.

Data quality

Describe the methods used to ensure data quality in full detail, including how many peaks are at FDR 5% and above 5-fold enrichment.

Software

Describe the software used to collect and analyze the ChIP-seq data. For custom code that has been deposited into a community repository, provide accession details.

Flow Cytometry

Plots

Confirm that:

- [ ] The axis labels state the marker and fluorochrome used (e.g. CD4-FITC).
- [ ] The axis scales are clearly visible. Include numbers along axes only for bottom left plot of group (a 'group' is an analysis of identical markers).
- [ ] All plots are contour plots with outliers or pseudocolor plots.
- [ ] A numerical value for number of cells or percentage (with statistics) is provided.

Methodology

Sample preparation

Describe the sample preparation, detailing the biological source of the cells and any tissue processing steps used.

Instrument

Identify the instrument used for data collection, specifying make and model number.

Software

Describe the software used to collect and analyze the flow cytometry data. For custom code that has been deposited into a community repository, provide accession details.

Cell population abundance | *Describe the abundance of the relevant cell populations within post-sort fractions, providing details on the purity of the samples and how it was determined.*

Gating strategy | *Describe the gating strategy used for all relevant experiments, specifying the preliminary FSC/SSC gates of the starting cell population, indicating where boundaries between "positive" and "negative" staining cell populations are defined.*

☐ Tick this box to confirm that a figure exemplifying the gating strategy is provided in the Supplementary Information.

Magnetic resonance imaging

Experimental design

Design type	Task fMRI, structural MRI
Design specifications	The HCP task fMRI dataset comprises seven tasks (14 tfMRI runs) with a total scan time of approximately one hour. Three tasks were acquired in one session and the remaining four in a separate session. Full details are provided in the HCP S1200 Release Reference Manual (https://www.humanconnectome.org/storage/app/media/documentation/s1200/HCP_S1200_Release_Reference_Manual.pdf).
Behavioral performance measures	In-scanner task performance data (reaction time and accuracy) were recorded for tasks requiring a subject response to verify task completion.

Acquisition

Imaging type(s)	functional, structural
Field strength	3T
Sequence & imaging parameters	Task fMRI data were acquired using whole-brain EPI sequences with a 32-channel head coil on a customized 3T Siemens Skyra scanner (TR = 720 ms, TE = 33.1 ms, flip angle = 52°, bandwidth = 2290 Hz/Px, in-plane FOV = 208 × 180 mm, 72 slices, 2.0 mm isotropic voxels, multiband acceleration factor = 8). Structural images were acquired using a T1-weighted MPRAGE sequence (TR = 2400 ms, TE = 2.14 ms, TI = 1000 ms, flip angle = 8°, FOV = 224 × 224 mm, isotropic voxel size = 0.7 mm).
Area of acquisition	Whole brain

Diffusion MRI ☐ Used ☒ Not used

Preprocessing

Preprocessing software	Preprocessing was performed using the HCP pipelines (version 3), which were specifically developed to leverage the high quality of the HCP data. Full details are available at https://github.com/Washington-University/HCPpipelines.
Normalization	Normalization was performed using the Multimodal Surface Matching (MSM-All) method, which integrates cortical areal features from folding and resting-state fMRI into a joint multimodal framework to improve intersubject alignment of task fMRI data (Glasser et al., 2016).
Normalization template	To enhance signal-to-noise ratio (SNR) and achieve dimensionality reduction while preserving individual variability, imaging analyses were conducted using the HCP-MMP1.0 cortical parcellation (180 regions per hemisphere), derived directly from multimodal HCP data (Glasser et al., 2016).
Noise and artifact removal	For task fMRI, within-subject fixed-effects analyses were conducted using the minimal preprocessing pipeline, which included motion correction, distortion correction, registration to standard space, high-pass filtering (200 s), spatial smoothing (4 mm FWHM), slice-timing correction, temporal autocorrelation correction, and model estimation with a general linear model (GLM) implemented in FSL's FILM (Glasser et al., 2013).
Volume censoring	To minimize loss of information, volume censoring was not applied in the HCP preprocessing pipeline; instead, alternative noise-removal techniques described above were employed.

Statistical modeling & inference

Model type and settings	Within-subject fixed-effects analyses were summarized above and are comprehensively detailed in Glasser et al. (2013). To examine sex differences, we applied linear mixed-effects models, including age as a fixed effect and a nested random effect term for person|family.
Effect(s) tested	We examined both task-by-sex interaction effects and main effects of sex.

Specify type of analysis: ☒ Whole brain ☐ ROI-based ☐ Both

Statistic type for inference (See Eklund et al. 2016)	Statistical comparisons were performed within each of the 360 regions defined by the HCP-MMP1.0 cortical parcellation.

Correction | False discovery rate (FDR) correction was applied to adjust for multiple comparisons across 360 cortical regions.

Models & analysis

n/a	Involved in the study
☐	☐ Functional and/or effective connectivity
☐	☐ Graph analysis
☐	☒ Multivariate modeling or predictive analysis

Functional and/or effective connectivity

Report the measures of dependence used and the model details (e.g. Pearson correlation, partial correlation, mutual information).

Graph analysis

Report the dependent variable and connectivity measure, specifying weighted graph or binarized graph, subject- or group-level, and the global and/or node summaries used (e.g. clustering coefficient, efficiency, etc.).

Multivariate modeling and predictive analysis

Machine learning models were used to predict sex independently from brain activation patterns across seven task fMRI experiments, cortical volumes derived from T1-weighted structural MRI, and behavioral measures collected from the same cohort of participants.We applied a partial least squares discriminant analysis (PLS-DA) model using repeated 10-fold cross-validation for sampling and the partial least squares algorithm for model tuning. The model was optimized by selecting the number of PLS components that maximized performance, as measured by the area under the receiver operating characteristic (ROC) curve. PLS-DA integrates dimensionality reduction with discriminant analysis in a single framework and is widely used for predictive modeling of high-dimensional data because it makes no assumptions about data distribution, offers flexibility, and remains linear in its parameters, thereby enhancing interpretability.
