# Supplementary material for: Robust but independent sex differences in human brain function, structure, and behavior
Source: Nat Commun. 2026 May 21;17:6694. doi: 10.1038/s41467-026-73262-2 (PMC13385741; doi:10.1038/s41467-026-73262-2)
Supplement: Supplementary file 15 — Transparent Peer Review file [file 41467_2026_73262_MOESM15_ESM.pdf]

nature portfolio

Peer Review File

# Robust but independent sex differences in human brain function, structure and behavior

Corresponding Author: Dr Armin Raznahan

**This file contains all reviewer reports in order by version, followed by all author rebuttals in order by version.**

Version 0:

Reviewer comments:

Reviewer #1

(Remarks to the Author)
In this manuscript, Liu and colleagues report a comprehensive set of analyses performed to determine sex differences in brain function, structure, and behavior.

They outline 4 key findings: (1) there are sex differences in average regional brain activation patterns across 7 different tasks and observed regional sex differences are sex-specific, (2) regional sex differences in brain activation are not spatially coupled to regional sex differences in brain volume, (3) brain activation, brain volume, and behavioral measures can be used to predict an individual's sex, but the sex predictions are only weakly correlated across measures, (4) associations between brain activation patterns and behaviors are largely shared across the sexes.

Major strengths include the use of a large cohort; extensive examination of sex differences in brain activation, brain structure, and behaviors, as well as relationships between them; and the implementation of strict tests to ensure their results are valid. These results are likely to be of broad interest to researchers. However, I do have one major concern and a suggestion for improvement. Please find them, along with other minor points, below.

I'm signing my review and am happy to be contacted by the authors if they have any questions or require clarification.
- Elvisha Dhamala

First, I am concerned about the data leakage in these analyses. Authors first used the HCP-YA cohort to identify sex differences in brain activation, brain structure, and behaviors. They then specifically used the regions and behaviors that exhibited sex differences as their input for the machine learning models to predict sex. This may be contributing to inflated accuracies for the sex prediction analyses. To prevent data leakage, the sample used to identify the sex differences should be distinct from the sample in which sex is predicted.

Second, authors used the HCP-YA cohort for these analyses which includes participants between the ages of 22-37. This range may cover perhaps the most "stable" time period in terms of brain function, structure, and behavior across the sexes (relative to earlier in life or later in life). However, there are many reported sex differences in these brain and behavioral measures in developmental and aging populations. It would be helpful if the authors could briefly comment on how the findings reported here may or may not translate to other ages.

1. There are a lot of non-standard acronyms used throughout the manuscript (e.g., SDAs, tsSDAs, tgSDAs, SDVs, SDBs) that decrease the readability of the manuscript.
2. Figure S4d - it is hard to distinguish between the triangles and squares denoting sex.
3. Figure S4d, Figure 1C - I find the use of line graphs a bit confusing here as the order of the tasks along the x axis is not necessarily meaningful. It might also be more informative if all 4 clusters were shown on a single graph to make it easier to compare how they differ across tasks.
4. Figure S10c, typo: "relative"
5. The figure captions for Figure S11c-d are a bit confusing.
6. Figure S13, typo: "beharviorial"
7. Figure S13, what do the labels "sensory", "motor", "alertness", "emotion", "personality", "in-scanner task performance",

“cognition”, “substance use”, and psychiatric and life function” correspond to?

Reviewer #2

(Remarks to the Author)
This paper has four major aims, all of which aim to improve our understanding of cerebral sex differences.
First, the authors examine sex differences in brain activation during a variety of fMRI tasks. They ask whether these sex differences vary by task or are consistent across tasks. They find that sex differences in brain activation do exist, but most are task-specific (with the exception of sensorimotor regions, which show a female bias). These task-specific differences can be reduced to four clusters of regional brain activity. Importantly, sex differences in performance did not drive or bias these differences in activation.
Second, the paper asks whether sex differences in brain activation (SDAs) overlap with sex differences in brain volume (SDVs). They find that these occur in largely different parts of the brain, with little to no spatial correspondence. Even in regions where females show both larger volumes and greater activation (e.g., motor cortices), there is no interindividual correlation: Having a larger region doesn’t mean that there is stronger activation.
Third, the authors examine how brain activation, brain volume, and behavior predict sex classification. They show that multivariate patterns of brain activity, brain anatomy, and behavior are all highly predictive of participant sex. However, these predictors are orthogonal: being sex-typical in one domain (e.g., structure) doesn’t mean being sex-typical in another (e.g., function or behavior).
Fourth, the study looks at whether sex differences in brain activation can predict sex differences in behavioral performance. They first tested brain-behavior associations separately within each sex, and then compared whether these associations differed between sexes. The results show that brain-behavior associations are largely conserved across males and females. The few subtle differences that do exist are not concentrated in sex-biased behaviors.

Overall, this paper makes a significant contribution to the literature on sex differences in the brain and is of high quality.

My main concern is that the paper contains a substantial amount of main and secondary analyses, and that the neuroscience jargon is quite heavy, which makes it difficult for readers of a multidisciplinary journal to parse and remember.

My main suggestion would be to try to alleviate the neuroscience jargon and move some of the secondary (yet very interesting!) analyses to the supplements, so that we can focus on your four main research questions and results. For instance, the authors may choose to move the analyses “to understand better how SDAs relate to independent descriptions of functional neuroanatomy” to the supplements.

Minor comments
1. Why was the sex x age interaction omitted from the models?
2. “These SDVs have been previously described in the HCP dataset50 and closely resemble those reported in independent datasets54.” I believe you meant to cite reference 53 and not 54.
3. You state that the task-specific sex differences are highly debated. How do your findings support and conflict with the previous literature?
4. It’s hard to believe there’s a sex difference when looking at Supplementary Fig. 4e.
5. Could you discuss why we might see most sex differences in clusters for gambling, compared to other tasks?
6. You looked at the spatial convergence of sex differences in brain activation during tasks and brain anatomy, but what about brain activation at rest? Wouldn’t we expect more concordance between brain activation at rest and brain anatomy?

Reviewer #3

(Remarks to the Author)
In the current work Liu and co-authors investigate task-based activation and behaviour in the HCP dataset to understand the brain basis of sex differences and how it relates to behavioral outcomes. They find there is if at all a very weak link between task-based activity patterns and behavioral differences between sexes, even if both measures show differences.

Overall, it is nice to see evidence of sex-difference in brain and behaviour but not a direct link. I think also the approach was concise and well articulated in the paper. I have various comments that I hope will improve the work further.

1. It is great the study uses all the benefits of HCP, however a feeling of 'again' HCP is also there for me. Would these findings reproduce elsewhere?
2. For the discussion I am missing a bit the interpretation of the why? If these levels are not really connected, why could this be the case? What are things that can or should be investigate to explain what is going on? Are similar observations made in other domains/species?
3. Did the authors take into account the reliability of the variables when investigating sex difference? Could it be explained by low reliability, high variance in one sex and not the other for example?
4. Why are the GMV findings in this study different than those published with the same dataset / authors in PNAS?

Version 1:

Reviewer comments:

Reviewer #1

(Remarks to the Author)
Authors have addressed all of my comments.

(Remarks on code availability)
I get a 404 error with the link provided.

Reviewer #2

(Remarks to the Author)
The authors have addressed all of my comments. I look forward to citing this publication.

(Remarks on code availability)
The URL does not work for me: https://github.com/siyuanliu8/HCPsexdifferences

Reviewer #3

(Remarks to the Author)
My comments have been addressed! Thanks!

(Remarks on code availability)

**Open Access** This Peer Review File is licensed under a Creative Commons Attribution 4.0 International License, which permits use, sharing, adaptation, distribution and reproduction in any medium or format, as long as you give appropriate credit to the original author(s) and the source, provide a link to the Creative Commons license, and indicate if changes were made.
In cases where reviewers are anonymous, credit should be given to 'Anonymous Referee' and the source.
The images or other third party material in this Peer Review File are included in the article's Creative Commons license, unless indicated otherwise in a credit line to the material. If material is not included in the article's Creative Commons license and your intended use is not permitted by statutory regulation or exceeds the permitted use, you will need to obtain permission directly from the copyright holder.
To view a copy of this license, visit https://creativecommons.org/licenses/by/4.0/

**Response to Reviewers**

We thank the reviewers for their insightful feedback and constructive suggestions, which have strengthened our manuscript. We have revised the paper accordingly and provide our point-by-point responses below. For clarity, the original reviewer comments are in black, and our responses are in blue. Any new or altered text from the manuscript is quoted, *italicized*, and followed by the corresponding line number. The revised manuscript (with changes highlighted in yellow), figures, and supplementary materials are attached.

## REVIEWER COMMENTS

### Reviewer #1 (Remarks to the Author):

**Comment 1**

In this manuscript, Liu and colleagues report a comprehensive set of analyses performed to determine sex differences in brain function, structure, and behavior.

They outline 4 key findings: (1) there are sex differences in average regional brain activation patterns across 7 different tasks and observed regional sex differences are sex-specific, (2) regional sex differences in brain activation are not spatially coupled to regional sex differences in brain volume, (3) brain activation, brain volume, and behavioral measures can be used to predict an individual's sex, but the sex predictions are only weakly correlated across measures, (4) associations between brain activation patterns and behaviors are largely shared across the sexes.

Major strengths include the use of a large cohort; extensive examination of sex differences in brain activation, brain structure, and behaviors, as well as relationships between them; and the implementation of strict tests to ensure their results are valid. These results are likely to be of broad interest to researchers. However, I do have one major concern and a suggestion for improvement. Please find them, along with other minor points, below.

I'm signing my review and am happy to be contacted by the authors if they have any questions or require clarification.
- Elvisha Dhamala

**Response 1**
We thank Prof. Dhamala for her positive and constructive feedback. We have addressed the helpful suggestions for improvement as detailed below.

**Comment 2**
First, I am concerned about the data leakage in these analyses. Authors first used the HCP-YA cohort to identify sex differences in brain activation, brain structure, and behaviors. They then specifically used the regions and behaviors that exhibited sex differences as their input for the machine learning models to predict sex. This may be contributing to inflated accuracies for the sex prediction analyses. To prevent data leakage, the sample used to identify the sex differences should be distinct from the sample in which sex is predicted.

**Response 2**
We fully agree with the reviewer on this well-made point and have now added the requested sensitivity analyses where available data exist. Because there are no alternative datasets with similar task fMRI and behavioral measures as the HCP, we are only able to test for stability of accuracy for sex prediction based on independent structural MRI data, which we sourced from the UK Biobank dataset.

In these additional sensitivity analyses, we processed structural MRI data from a subset of the UK Biobank of similar size to the HCP dataset and mapped significant sex differences in gray matter volume (GMV), surface area (SA), and cortical thickness (CT) within these data, controlling for age and relevant global measures (**Supplementary Fig. 12a**). The effect sizes maps of sex differences derived from UK Biobank and HCP were highly correlated across brain regions for all three metrics (r>0.63 and Pspin<0.0001, **Supplementary Fig. 12b**). As expected from this close spatial correspondence, sex-prediction accuracy and Sex Typicality Scores in the HCP dataset (**Supplementary Fig. 12c**) were essentially unchanged in new supplementary analyses that prevented data leakage by using a mask of sex differences defined in the UK Biobank dataset rather than the HCP dataset. These new analyses, results and a **Supplementary Fig. 12** have been added to the manuscript and are reproduced below.

<u>Results (Lines 265-278):</u> "*It is important to note that these sex-prediction accuracy and associated sex typicality scores are based on 10-fold cross-validation where the definition of sex-biased brain regions and behavioral traits for input to the Partial Least Squares Discriminant Algorithm was conducted in the same HCP dataset where held-out individuals were used to test model performance. Because the HCP dataset is unique in its large battery of fMRI tasks and behavioral traits, we were only able to directly test for evidence of potential data leakage in analyses based on structural MRI data, which are extensively available in large independent datasets such as the UK Biobank[65]. We therefore defined maps of sex-biased cortical gray matter volume, surface area, and cortical thickness in this independent dataset (**Methods**) — which were highly similar to those in the HCP dataset (spatial correlations > 0.63; **Supplementary Fig. 12a,b**) — and used these to define input data for Partial Least Squares Discriminant Algorithm classification of sex in the HCP dataset. These supplementary analyses found almost identical sex prediction accuracy and Sex Typicality Scores as our primary analyses in the HCP (**Supplementary Fig. 12c**), arguing for good generalizability of our findings for structural MRI across datasets.*"

<u>Methods (Lines 544-555):</u> "*We additionally leveraged the UK Biobank dataset[65] (www.ukbiobank.ac.uk) as an independent replication cohort in sensitivity analyses designed to validate the regional distribution of sex differences and sex prediction performance in brain structure observed in the HCP dataset. Because the structure of task fMRI and behavioral data differs substantially between the HCP and UK Biobank datasets, these validation analyses were restricted to brain anatomy, for which high-quality structural MRI data are available in both cohorts. To closely match the age range and degrees of freedom between datasets, we selected the youngest subset of healthy UKB participants (44–50 years; 529 males and 637 females) who were scanned at a single site (site #11025). T1-weighted anatomical images (1-mm isotropic resolution) from these participants were processed using the same structural MRI pipeline applied to the HCP*

*dataset. Details regarding UK Biobank participant recruitment and data acquisition procedures are described in Miller et al., 2016[78]. All participants provided informed consent.”*

Methods (Lines 816-841): “***Cross-validation of anatomical sex differences and sex prediction in UK Biobank dataset***

*In two sensitivity analyses, we assessed the spatial reproducibility of sex differences in brain anatomy using an independent UK Biobank dataset and evaluated whether sex prediction performance in the HCP dataset was affected when the masks of significant sex differences in brain anatomy used to define training data were derived from the UK Biobank dataset (as opposed to an HCP dataset mask as in our primary analyses).*

*Structural MRI data from the UK Biobank sample were preprocessed using the same FreeSurfer pipeline applied to the HCP dataset. Two participants were excluded due to poor surface reconstruction quality (Euler number < −217), resulting in a final sample of 528 males (49.0[1.0] yrs) and 636 females (48.9[1.1] yrs). Using the same Model 4, we identified significant regional sex differences (q < 0.05) in gray matter volume, surface area, and cortical thickness beyond global measures in the UK Biobank sample (**Supplementary Fig. 12a**).*

*First, to quantify the spatial correspondence of sex differences between the HCP and UK Biobank datasets, we computed spatial correlations of effect sizes (Cohen’s d) across the 360 regions defined by the HCP parcellation. Statistical significance of this spatial correlation was assessed using spin tests with 10k spatial permutations (**Supplementary Fig. 12b**).*

*Second, to address potential inflation of sex prediction accuracy resulting from using the same dataset to define sex differences and perform prediction, we repeated the machine-learning analyses described above using masks of significant sex differences derived from the UK Biobank dataset (**Supplementary Fig. 12c**), rather than from the HCP dataset (original results shown in **Supplementary Fig. 10c**). We then quantified changes in prediction accuracy between these two conditions (**Supplementary Fig. 12c**). We also compared model outputs by computing Pearson correlations of sex typicality scores (STS) across participants between the primary model based on a sex difference mask defined in the HCP and the alternative model where this mask was defined in the UK Biobank (**Supplementary Fig. 12c**).”*

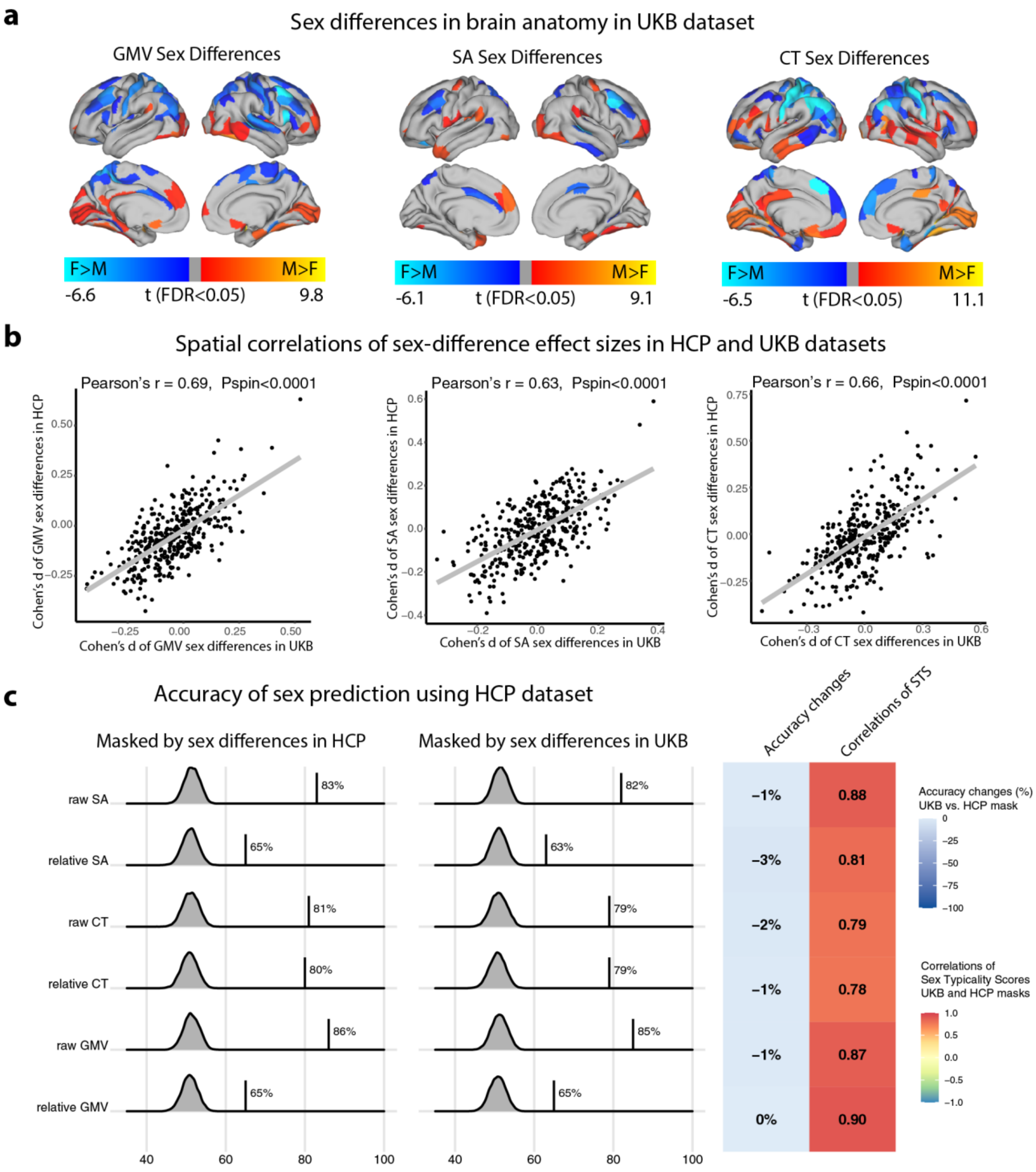


***Supplementary Fig. 12: Cross-validation of sex differences and sex prediction performance in brain anatomy using an independent UK Biobank dataset.***

*(**a**) We repeated the structural MRI analyses applied to the primary HCP dataset in an independent UK Biobank (UKB) sample and identified statistically significant sex differences in regional gray matter volume (GMV), surface area (SA), and cortical thickness (CT). Color maps show t values for regional sex differences after controlling for corresponding global measures (total GMV, total SA, and mean CT) and correcting for*

*multiple comparisons across brain regions (FDR q < 0.05). (**b**) Scatter plots showing strong spatial correspondence between structural sex differences derived from the UKB (x-axis) and HCP (y-axis) datasets. Each point represents Cohen's d for one of 360 brain regions defined by the HCP parcellation. Effect sizes were highly correlated (r > 0.63) and significantly exceeded chance levels based on 10,000 spin permutations (Pspin<0.0001). (**c**) Effects of using different datasets to define the mask of significant sex differences for sex prediction. In the left column, we reproduced the original sex prediction accuracy for each anatomical feature set (bars; see **Supplementary Fig. 10c**), where the HCP dataset was used for both mask definition and prediction, relative to a null distribution generated from 10,000 sex-label permutations. In the right column, masks of significant sex differences derived from the UKB dataset (panel **a**) were used as model inputs, and sex prediction was performed in the HCP dataset. The first color bar indicates changes in prediction accuracy between the two approaches, showing only a modest reduction (<3%) when using the independent UKB dataset for masking. The second color bar shows high correlations (r > 0.78) between sex typicality scores (STS) across participants obtained from the two modeling approaches, indicating that shared interindividual information was used for sex prediction in both cases.*

**Comment 3**

Second, authors used the HCP-YA cohort for these analyses which includes participants between the ages of 22-37. This range may cover perhaps the most "stable" time period in terms of brain function, structure, and behavior across the sexes (relative to earlier in life or later in life). However, there are many reported sex differences in these brain and behavioral measures in developmental and aging populations. It would be helpful if the authors could briefly comment on how the findings reported here may or may not translate to other ages.

**Response 3**

We thank the reviewer for raising this important point and have now added this in Discussion.

<u>Discussion (Lines 491-495):</u> "*It may also be that sex differences in the neuroimaging and behavioral features examined herein show stronger inter-relationships during dynamic windows of sex-biased brain development[17,79,80] and ageing[81]. It will be important to test these hypotheses in future work as necessary multi-task and multimodal datasets in these lifespan stages grow.*"

**Comment 4**

Minor comment: There are a lot of non-standard acronyms used throughout the manuscript (e.g., SDAs, tsSDAs, tgSDAs, SDVs, SDBs) that decrease the readability of the manuscript.

**Response 4**

We fully agree with this concern and found this issue challenging as well. We now have simplified these novel abbreviations by replacing tsSDA and tgSDA with just SDA (using

words to specify if these are task-specific or task-general). We felt bound to retain SDA, SDV and SDB given word count limits, and our recurrent usage of these terms.

In addition to simplifying the novel abbreviations, we have now also carefully reviewed the manuscript and reduced abbreviation usage elsewhere whenever possible — resulting in an overall removal of ~ 50% of abbreviations.

Finally, to further improve readability, we spelled out all abbreviations in headings and figure titles and redefined any abbreviations used in figure legends.

**Comment 5**

Minor comment: Figure S4d - it is hard to distinguish between the triangles and squares denoting sex.

Minor comment: Figure S4d, Figure 1C - I find the use of line graphs a bit confusing here as the order of the tasks along the x axis is not necessarily meaningful. It might also be more informative if all 4 clusters were shown on a single graph to make it easier to compare how they differ across tasks.

**Response 5**

We thank the reviewer for raising these points, which we have addressed through 3 changes.

First, we have now substantially revised **Fig. S4d,e** (new **Fig. S4e,f**) to replace the triangle and square points connected by lines with violin plot distributions for activation in males and females – separately for each task in each cluster. This new figure does not have connecting lines between tasks, and now newly emphasizes the overlap in activation distributions between sexes.

Second, we have also added a new panel **Fig. S4d** which uses a heatmap to show all 4 clusters on a single plot to facilitate comparison across tasks.

Third, we have added new text to the legends of **Fig. 1c** and **1e** to clarify the intended purpose of the line graphs. As the reviewer correctly noted, the tasks along the x-axis in **Fig. 1c,e** are ordered alphabetically and do not imply any specific sequencing. Rather, the connecting lines are intended to illustrate how sex differences in activation vary across tasks. Specifically, they highlight task-dependent variation in sex differences across the four clusters exhibiting task-specific sex differences in activation (**Fig. 1c**), in contrast to the task-invariant pattern observed in regions showing task-general sex differences in activation (**Fig. 1e**).

The new **Fig. 1** legend and new supplementary **Fig. S4d,e,f** with legend are reproduced below.

**Fig. 1: Humans show task-specific and task-general sex differences in brain activation.**

… (**c**) *Each cluster exhibited a distinct line-graph profile of sex differences in activation across the seven tasks, reflected by task-dependent variation in t values comparing males and females that differed between clusters;* … (**e**) *Profiles of sex differences in activation for regions exhibiting task-general sex differences in activation across all seven tasks, represented by line graphs, showing consistently negative t values (i.e., female-biased activation) for the male–female contrast, indicating sex differences that are independent of task variation…*

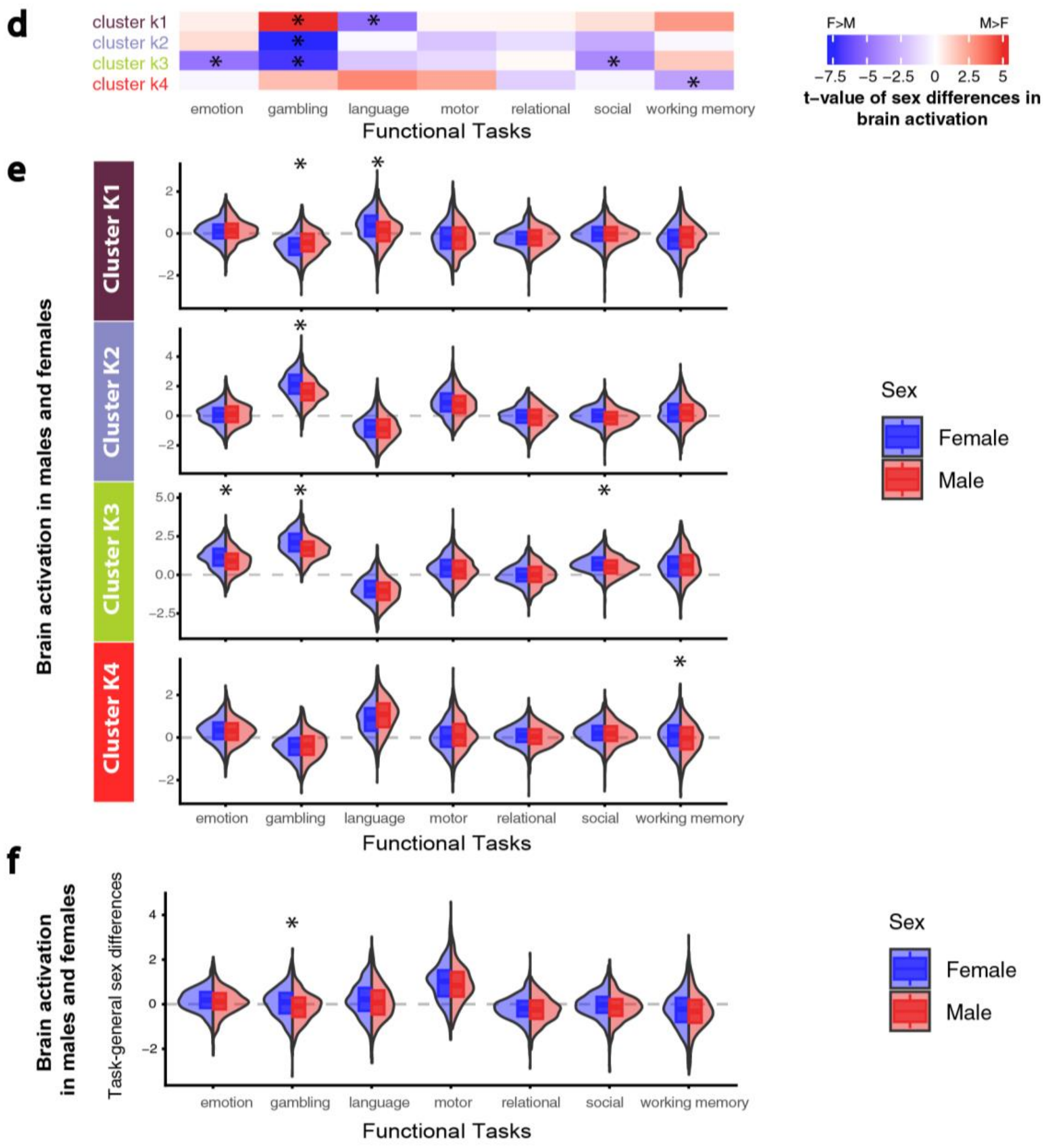


**Supplementary Fig. 4: k-mean stability of task-specific sex differences in brain activation and sex-stratified brain activation profiles across tasks.**

*… (**d**) Heatmap showing t values of sex differences in brain activation across tasks for all four clusters of task-specific sex differences in activation (**Fig. 1b,c**), facilitating comparisons across clusters. (**e,f**) Combined violin and box plots showing male (in red) vs. female (in blue) distributions of brain activation in each task for the four clusters of task-specific sex differences in activation (**Fig. 1b,c**) and for regions exhibiting task-general sex differences in activation (**Fig. 1d,e**). Consistent with their definitions, the four task-specific clusters show no consistent direction of sex bias across the seven tasks (panel **e**), whereas regions exhibiting task-general sex differences show consistently greater activation in females than in males across tasks (panel **f**). In d-f, Asterisks indicate statistically significant sex differences in activation for a given task following Bonferroni correction ($P < 0.05/7/4$ for the four clusters of task-specific sex differences in activation in panel **d**, **e**; $P < 0.05/7$ for regions exhibiting task-general sex differences in*

*activation in panel **f**). Exact P values and effect sizes for all regions of interest are reported in **Supplementary Table 4**.*

**Comment 6**

Minor comment: Figure S10c, typo: "relative"

**Response 6**

We thank the reviewer for pointing this out and the typo has been corrected in **Fig. S10c**.

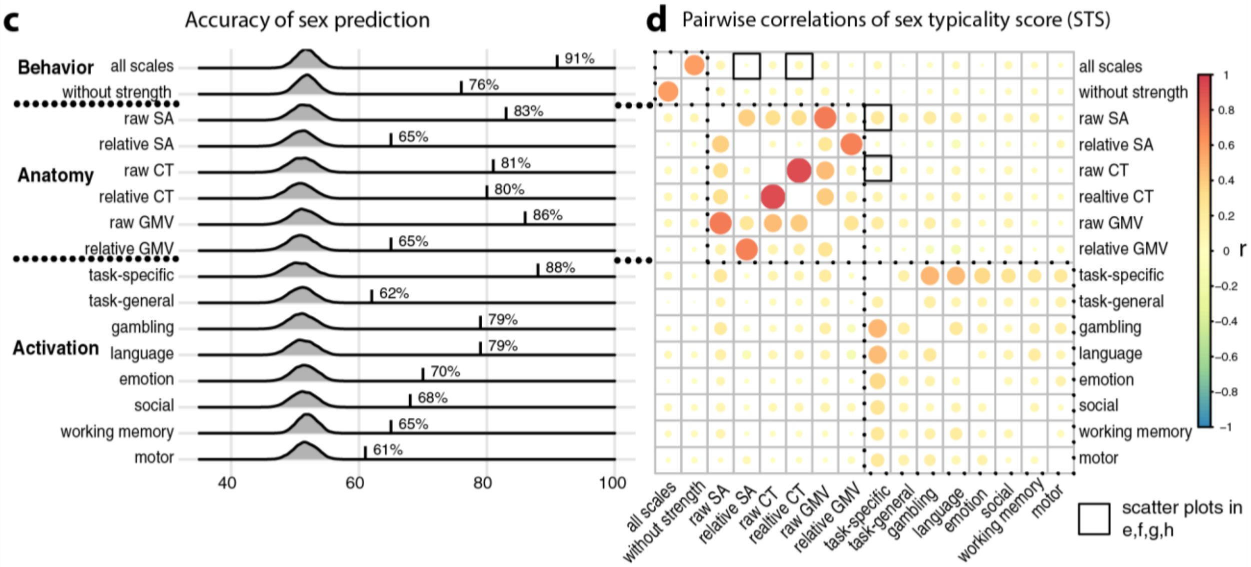


**Comment 7**

Minor comment: The figure captions for Figure S11c-d are a bit confusing.

**Response 7**

We agree with the reviewer and have revised the legend for **Fig. S11c,d** to clarify this point.

**Supplementary Fig. 11: Person-level sex typicality scores and percentage of individuals whose sex was misclassified across 16 sex prediction models** … (**c,d**) *Histograms showing the proportion of individuals as a function of the number of models (0–16) in which their sex was misclassified, separately for females (**c**) and males (**d**). For example, the bin at x = 0 in panel **c** represents the percentage of females who were misclassified in none of the 16 models—that is, females who were correctly classified by all models. Using this criterion, only 6.2% of females (panel **c**) and 1.4% of males (panel **d**) were correctly classified across all 16 models, corresponding to the leftmost bins in each panel.*

**Comment 8**

Minor comment: Figure S13, typo: "beharviorial"

**Response 8**

We thank the reviewer for pointing this out and the typo has been corrected in **Fig. S14** (original **S13**), reproduced in response 9 below.

**Comment 9**

Minor comment: Figure S13, what do the labels “sensory”, “motor”, “alertness”, “emotion”, “personality”, “in-scanner task performance”, “cognition”, “substance use”, and psychiatric and life function” correspond to?

**Response 9**

We thank the reviewer for pointing this out. These labels correspond to the behavioral categories defined by the HCP consortium, and this information has now been added to **Fig. S14** (original **S13**), reproduced below.

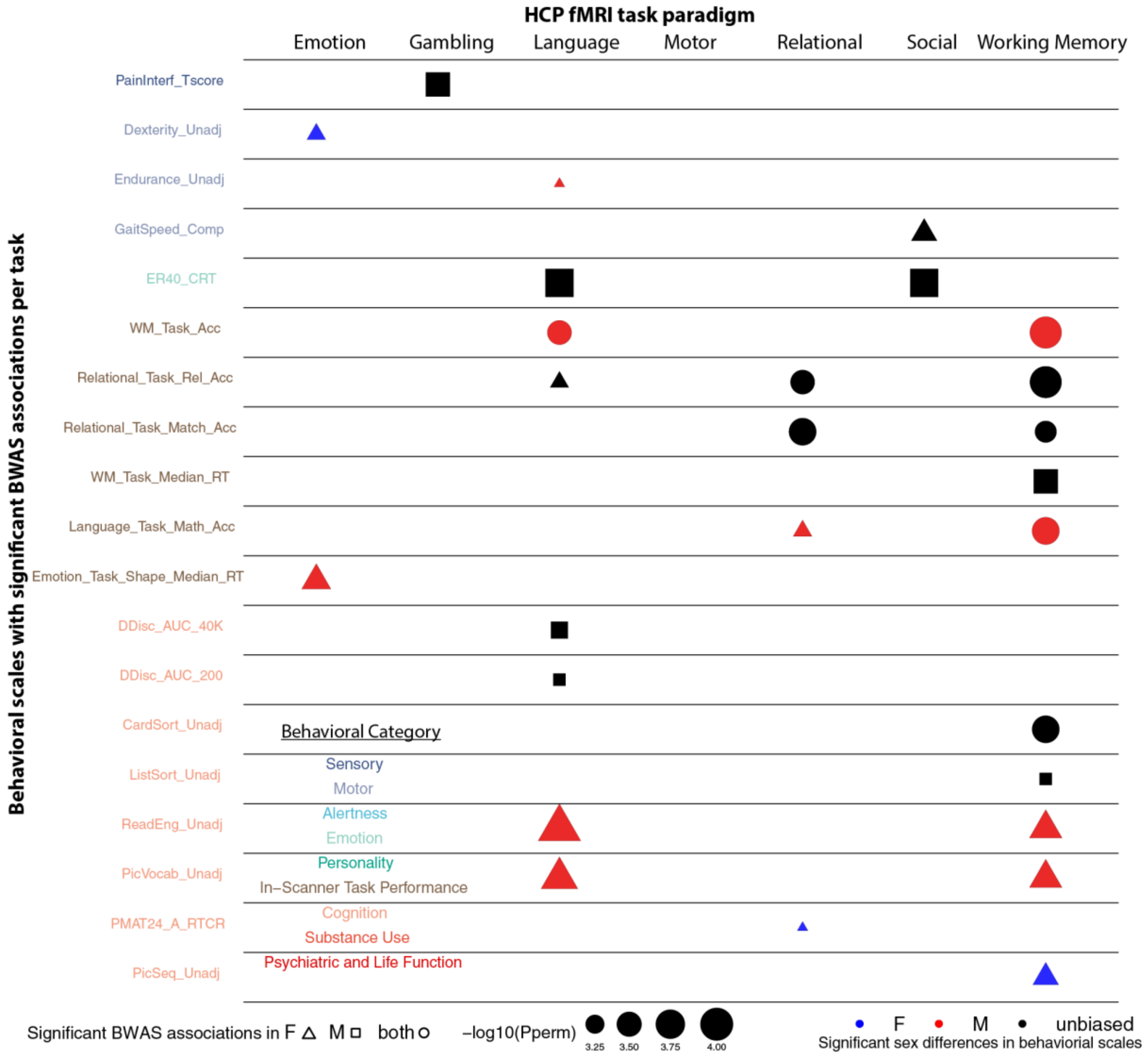

## Reviewer #2 (Remarks to the Author):

**Comment 1**
This paper has four major aims, all of which aim to improve our understanding of cerebral sex differences.

First, the authors examine sex differences in brain activation during a variety of fMRI tasks. They ask whether these sex differences vary by task or are consistent across tasks. They find that sex differences in brain activation do exist, but most are task-specific (with the exception of sensorimotor regions, which show a female bias). These task-specific differences can be reduced to four clusters of regional brain activity. Importantly, sex differences in performance did not drive or bias these differences in activation.

Second, the paper asks whether sex differences in brain activation (SDAs) overlap with sex differences in brain volume (SDVs). They find that these occur in largely different parts of the brain, with little to no spatial correspondence. Even in regions where females show both larger volumes and greater activation (e.g., motor cortices), there is no interindividual correlation: Having a larger region doesn't mean that there is stronger activation.

Third, the authors examine how brain activation, brain volume, and behavior predict sex classification. They show that multivariate patterns of brain activity, brain anatomy, and behavior are all highly predictive of participant sex. However, these predictors are orthogonal: being sex-typical in one domain (e.g., structure) doesn't mean being sex-typical in another (e.g., function or behavior).

Fourth, the study looks at whether sex differences in brain activation can predict sex differences in behavioral performance. They first tested brain-behavior associations separately within each sex, and then compared whether these associations differed between sexes. The results show that brain-behavior associations are largely conserved across males and females. The few subtle differences that do exist are not concentrated in sex-biased behaviors.

Overall, this paper makes a significant contribution to the literature on sex differences in the brain and is of high quality.

> **Response 1**
> We thank the reviewer for their thoughtful and comprehensive summary of our work. We appreciate their clear articulation of the study's four major aims and findings, as well as their recognition of the rigor and quality of the analyses.

**Comment 2**
My main concern is that the paper contains a substantial amount of main and secondary analyses, and that the neuroscience jargon is quite heavy, which makes it difficult for readers of a multidisciplinary journal to parse and remember.

My main suggestion would be to try to alleviate the neuroscience jargon and move some of the secondary (yet very interesting!) analyses to the supplements, so that we can focus on your four main research questions and results. For instance, the authors may choose to move the analyses "to understand better how SDAs relate to independent descriptions of functional neuroanatomy" to the supplements.

**Response 2**

We are grateful for the reviewer's constructive suggestion. We have moved the section mentioned to **Supplementary Text 1**, substantially shorted mention of these results in the main manuscript (see below), and merged **Fig. 1f** with the original **Fig. S6** to create a revised **Fig. S6**.

Results (Lines 165-172): "*To contextualize sex differences in brain activation (SDAs) against regional functional neuroanatomy, using Spin Tests*[44,45]*, we assessed their spatial correspondence with meta-analytic brain activation maps (Neurosynth*[46–48]*) and canonical resting-state connectivity networks (Yeo-Krienen 17 network parcellation*[49]*, **Supplementary Text 1**). Both task-specific and task-general SDAs were enriched within distributed systems supporting motor, attentional, language, and social processes, suggesting differential engagement of functional networks by males and females during task performance. Importantly however, this spatial alignment alone does not speak to whether SDAs are relevant for domains of behavior associated with each highlighted brain systems"*

Supplementary Text 1: "**Alignment of sex differences in brain activation with canonical functional systems and networks** To better understand how sex differences in activation (SDAs) relate to independent descriptions of functional neuroanatomy, we used Spin Tests[1,2] (**Methods**) to compare the spatial distribution of SDA clusters (**Fig. 1b,d**) with meta-analytically defined maps of brain activation for 24 different neuropsychological domains derived from latent discriminant analysis of text and reported activations for >11k fMRI studies (Neurosynth[3–5], **Supplementary Fig. 6a**). All 4 task-specific SDA and the task-general SDA cluster showed significant spatial overlaps with canonical functional brain systems — establishing a statistically significant concentration of SDAs with brain systems subserving motor function (K1, task-general SDA), attention and working memory (K2, K3), language (K3), and social cognition (K3, K4). We also found task-specific and task general SDAs to be enriched within distinct canonical functional brain networks defined by independent resting state fMRI data (Yeo-Krienen 17 network parcellation[6], **Methods**, **Supplementary Fig. 6b**). These observations imply that distinct functional systems can be differentially activated by males and females during execution of the same task. For example, the K2 cluster of task-specific SDA – which shows stronger mean activation in females than males during gambling – overlaps with brain systems that are involved in spatial attention and motor planning (**Supplementary Fig. 6a**) and reside within visual and dorsal attention resting state networks (**Supplementary Fig. 6b**). Thus, brain regions showing distinct SDA profiles are spatially aligned with distributed brain networks subserving different aspects of cognition, behavior

and emotion. However, this spatial alignment does not speak to the relevance of SDAs for any cognitive, behavioral or emotional traits.”

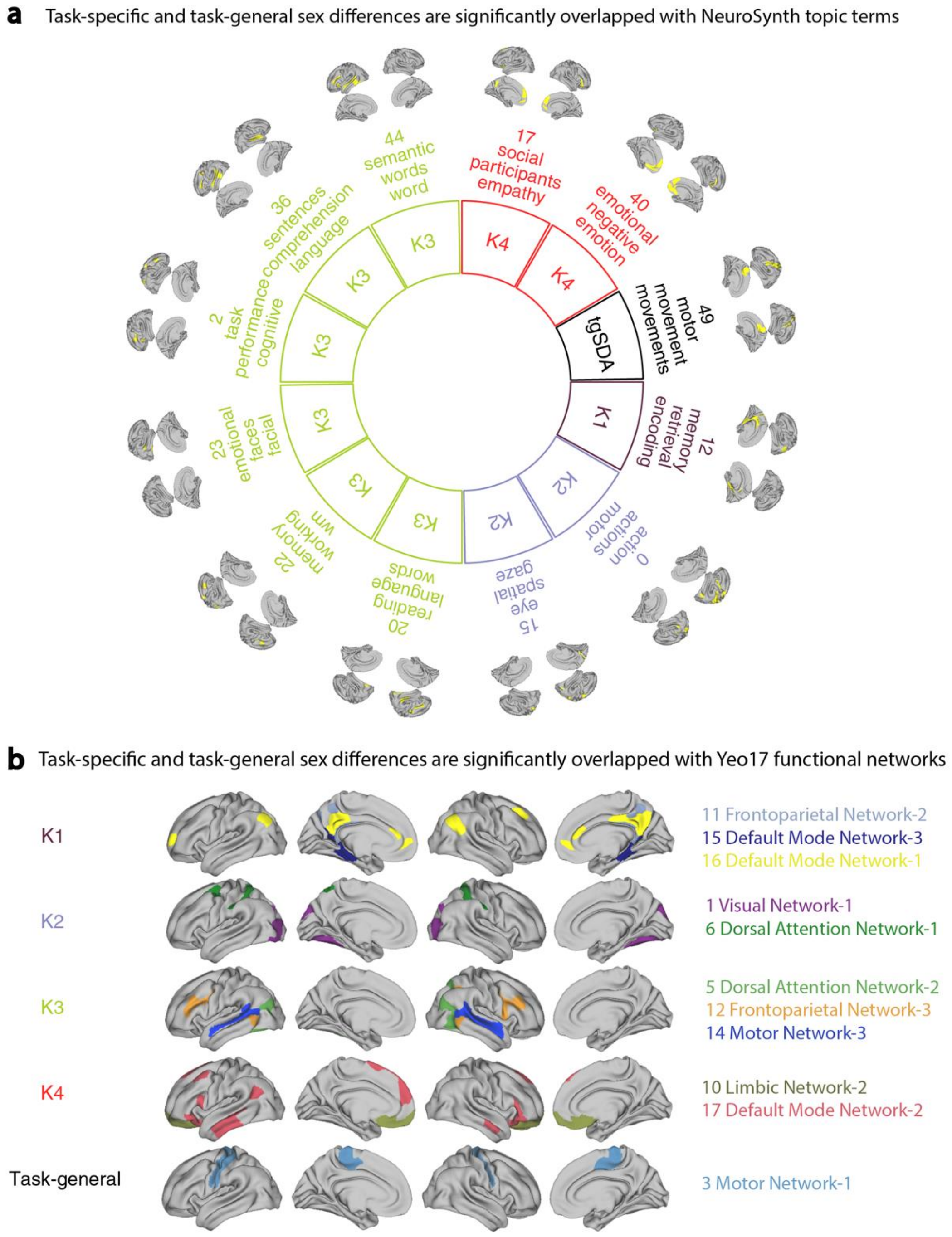


**Supplementary Fig. 6: Brain regions exhibiting significant sex differences in activation are enriched within canonical functional systems and networks.** (**a**) Conjunction maps (surface projections in the outer circle) for each of the metanalytic Neurosynth “topic terms” (texts in the middle circle) that showed regional activations which significantly overlapped with each cluster of task specific sex differences in activation (task-specific SDA) in (**a**) and with regions exhibiting

task-general sex differences in activation (task-general SDA) in (**b**) (labeled in the inner circle) based on Spin Tests with 10k spatial permutations and additional Bonferroni correction for 24 topic terms ($P_{spin}$< 0.05/24, **Methods**). NeuroSynth topics that significantly overlap with each cluster based on Spin Tests are referred to by the number and topic terms (https://neurosynth.org/analyses/topics/v4-topics-50/). Colored middle and inner circles match the cluster colors from (**Fig. 1b**). (**b**) Conjunction maps of the 4 task-specific SDA clusters (colors of K1-K4 match the cluster colors from **Fig. 1b**) and the 1 set of task-general SDA regions with those Yeo-Krienen resting state connectivity cortical networks[6] that showed statistically significant overlaps with SDAs based on 10k Spin Test permutations and Bonferroni correction of resulting empirical p values for multiple comparisons across 17 networks of the Yeo-Krienen parcellation ($P_{spin}$<0.05/17).

**Comment 3**
Minor comment: Why was the sex x age interaction omitted from the models?

**Response 3**
We thank the reviewer for highlighting the importance of a potential age-by-sex interaction. We tested this interaction in the primary Model 1; as it was not significant, it was not retained in the final model. This clarification has now been added to the manuscript.

Methods (Lines 641-642): "*We additionally tested for a sex-by-age interaction by including a Sex × Age term in Model 1; as this interaction was not significant in any brain region, it was excluded from the final model.*"

**Comment 4**
Minor comment: "These SDVs have been previously described in the HCP dataset50 and closely resemble those reported in independent datasets54." I believe you meant to cite reference 53 and not 54.

**Response 4**
We thank the reviewer for pointing this out and have corrected it.

Results (Lines 186-187): "*These SDVs have been previously described in the HCP dataset[50] and closely resemble those reported in independent datasets[53].*"

**Comment 5**
Minor comment: You state that the task-specific sex differences are highly debated. How do your findings support and conflict with the previous literature?

**Response 5**
We have now incorporated these suggested comparisons into the Discussion section of our manuscript as reproduced below.

Discussion (Lines 404-413): "*Task-specific sex differences were evident across multiple clusters of brain regions that exhibited sex differences in distinct individual tasks, including gambling, language, and social cognition, with gambling emerging across several clusters (potentially reflecting the robust capacity of this task to strongly modulate activity across the brain,* ***Supplementary Fig. 1****). These findings echo prior reports of sex differences observed in isolated task-based fMRI paradigms of gambling[29,30], verbal processing[22,23], and social interaction[31,32], as well as in behavioral studies of gambling-related decision-making and impulsivity[9,10]. Together with the small effect sizes observed here, this heterogeneity in task-specific sex differences in brain function may help explain the inconsistent findings reported in previous meta-analyses[4,33,34] of largely small-sample studies.*"

**Comment 6**

Minor comment: It's hard to believe there's a sex difference when looking at Supplementary Fig. 4e.

**Response 6**

We agree with the reviewer, as also noted by Reviewer 1 (Comment 5), that the original **Fig. S4d,e** (new **Fig. S4e,f**) could be misleading because it displayed only mean activation values for males and females. We have therefore revised this figure to use combined violin and box plots, which display the full distribution of brain activation for males and females in each task and for each set of brain regions exhibiting task-specific (new **Fig. S4e**) and task-general (new **Fig. S4f**) sex differences in activation.

Specifically, in new **Fig. S4f** (original **Fig. S4e**), females consistently show higher activation than males across all seven tasks within the set of regions exhibiting task-general sex differences. Although sex differences within individual tasks are not uniformly significant (reaching significance only for the gambling task, indicated by an asterisk), the consistent direction of female-biased activation across tasks gives rise to a statistically significant main effect of sex in Model 2. By definition, this pattern reflects sex differences that are independent of task context and therefore constitute task-general sex differences in activation.

For clarity, the new **Fig. S4e,f** (original **Fig. S4d,e**) and the updated legend are reproduced below.

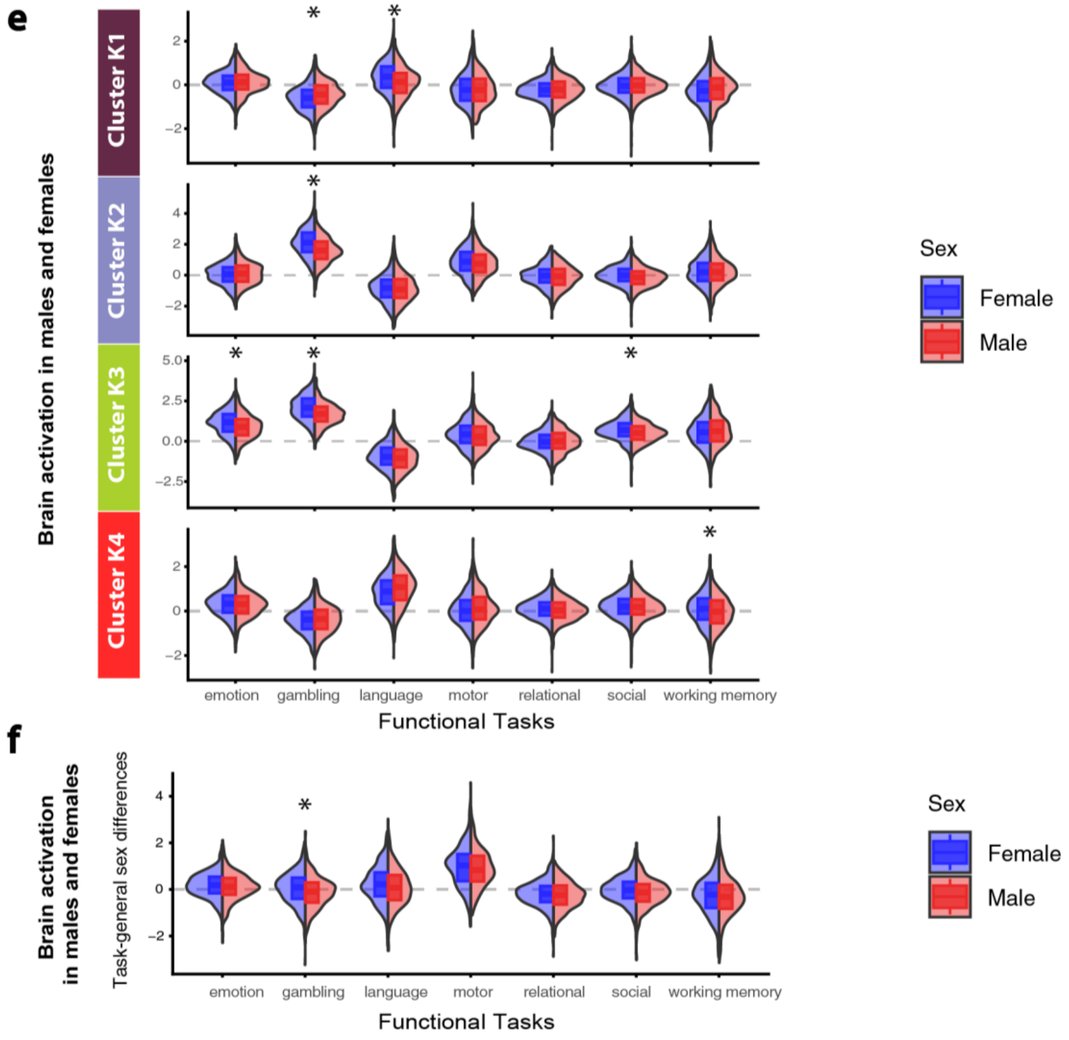


**Supplementary Fig. 4: k-mean stability of task-specific sex differences in brain activation and sex-stratified brain activation profiles across tasks.**

*… (**e,f**) Combined violin and box plots showing male (in red) vs. female (in blue) distributions of brain activation in each task for the four clusters of task-specific sex differences in activation (**Fig. 1b,c**) and for regions exhibiting task-general sex differences in activation (**Fig. 1d,e**). Consistent with their definitions, the four task-specific clusters show no consistent direction of sex bias across the seven tasks (panel **e**), whereas regions exhibiting task-general sex differences show consistently greater activation in females than in males across tasks (panel **f**). In d-f, Asterisks indicate statistically significant sex differences in activation for a given task following Bonferroni correction ($P < 0.05/7/4$ for the four clusters of task-specific sex differences in activation in panel **d**, **e**; $P < 0.05/7$ for regions exhibiting task-general sex differences in activation in panel **f**). Exact P values and effect sizes for all regions of interest are reported in **Supplementary Table 4**.*

**Comment 7**
Minor comment: Could you discuss why we might see most sex differences in clusters for gambling, compared to other tasks?

**Response 7**
We thank the reviewer for highlighting this interesting finding and have added a possible explanation in Discussion.

Discussion (Lines 404-413): "*Task-specific sex differences were evident across multiple clusters of brain regions that exhibited sex differences in distinct individual tasks, including gambling, language, and social cognition, with gambling emerging across several clusters (potentially reflecting the robust capacity of this task to strongly modulate activity across the brain,* ***Supplementary Fig. 1****). These findings echo prior reports of sex differences observed in isolated task-based fMRI paradigms of gambling*[29,30]*, verbal processing*[22,23]*, and social interaction*[31,32]*, as well as in behavioral studies of gambling-related decision-making and impulsivity*[9,10]*. Together with the small effect sizes observed here, this heterogeneity in task-specific sex differences in brain function may help explain the inconsistent findings reported in previous meta-analyses*[4,33,34] *of largely small-sample studies."*

**Comment 8**
Minor comment: You looked at the spatial convergence of sex differences in brain activation during tasks and brain anatomy, but what about brain activation at rest? Wouldn't we expect more concordance between brain activation at rest and brain anatomy?

**Response 8**
We thank the reviewer for raising this important point. We agree that sex differences in resting-state brain activation may plausibly show greater concordance with brain anatomy than task-evoked activation. However, an independent study provides preliminary evidence that this may not be the case. We briefly noted this point in the original Discussion and have now expanded it to provide a more detailed discussion.

Discussion (Lines 430-435): "Interestingly, our findings for task-based measures of brain activation are consistent with recent studies of sex-differences in resting state brain activation – which were also found to be independent of sex differences in cortical morphometry [74]. *Nevertheless, future studies that directly compare sex differences in task-evoked and resting-state functional organization alongside brain anatomy will be valuable for determining whether sex differences in resting-state function show greater spatial congruence with anatomical sex differences than task-evoked activation."*

**Reviewer #3 (Remarks to the Author):**

**Comment 1**

In the current work Liu and co-authors investigate task-based activation and behaviour in the HCP dataset to understand the brain basis of sex differences and how it relates to behavioral outcomes. They find there is if at all a very weak link between task-based activity patterns and behavioral differences between sexes, even if both measures show differences.

Overall, it is nice to see evidence of sex-difference in brain and behaviour but not a direct link. I think also the approach was concise and well articulated in the paper. I have various comments that I hope will improve the work further.

**Response 1**

We thank the reviewer for their thoughtful evaluation of our study and for their positive comments on the clarity and articulation of the approach. We appreciate their constructive feedback and address their helpful comments as detailed below.

**Comment 2**

It is great the study uses all the benefits of HCP, however a feeling of 'again' HCP is also there for me. Would these findings reproduce elsewhere?

**Response 2**

We fully agree with the reviewer on this well-made point and have now added the requested sensitivity analyses where available data exist. Because there are no alternative datasets with similar task fMRI and behavioral measures as the HCP, we are only able to test for stability of accuracy for sex prediction based on independent structural MRI data — which we sourced from the UK Biobank dataset. These analyses find a strong reproducibility of regional anatomical sex difference, sex prediction accuracy and sex typicality scores between the HCP and UK Biobank datasets — which are detailed in new text and figures in our manuscript provided below.

In these additional sensitivity analyses, we processed structural MRI data from a subset of the UK Biobank of similar size to the HCP dataset and mapped significant sex differences in gray matter volume (GMV), surface area (SA), and cortical thickness (CT) within these data, controlling for age and relevant global measures (**Supplementary Fig. 12a**). The effect sizes maps of sex differences derived from UK Biobank and HCP were highly correlated across brain regions for all three metrics ($r>0.63$ and $P_{spin}<0.0001$, **Supplementary Fig. 12b**). As expected from this close spatial correspondence, sex-prediction accuracy and Sex Typicality Scores in the HCP dataset (**Supplementary Fig. 12c**) were essentially unchanged in new supplementary analyses that prevented data leakage by using a mask of sex differences defined in the UK Biobank dataset rather than the HCP dataset. These new analyses, results and a **Supplementary Fig. 12** have been added to the manuscript and are reproduced below.

Results (Lines 265-278): "*It is important to note that these sex-prediction accuracy and associated sex typicality scores are based on 10-fold cross-validation where the definition of sex-biased brain regions and behavioral traits for input to the Partial Least Squares Discriminant Algorithm was conducted in the same HCP dataset where held-out individuals were used to test model performance. Because the HCP dataset is unique in its large battery of fMRI tasks and behavioral traits, we were only able to directly test for evidence of potential data leakage in analyses based on structural MRI data, which are extensively available in large independent datasets such as the UK Biobank[65]. We therefore defined maps of sex-biased cortical gray matter volume, surface area, and cortical thickness in this independent dataset (**Methods**) — which were highly similar to those in the HCP dataset (spatial correlations > 0.63; **Supplementary Fig. 12a,b**) — and used these to define input data for Partial Least Squares Discriminant Algorithm classification of sex in the HCP dataset. These supplementary analyses found almost identical sex prediction accuracy and Sex Typicality Scores as our primary analyses in the HCP (**Supplementary Fig. 12c**), arguing for good generalizability of our findings for structural MRI across datasets.*"

Methods (Lines 544-555): "*We additionally leveraged the UK Biobank dataset[65] (www.ukbiobank.ac.uk) as an independent replication cohort in sensitivity analyses designed to validate the regional distribution of sex differences and sex prediction performance in brain structure observed in the HCP dataset. Because the structure of task fMRI and behavioral data differs substantially between the HCP and UK Biobank datasets, these validation analyses were restricted to brain anatomy, for which high-quality structural MRI data are available in both cohorts. To closely match the age range and degrees of freedom between datasets, we selected the youngest subset of healthy UKB participants (44–50 years; 529 males and 637 females) who were scanned at a single site (site #11025). T1-weighted anatomical images (1-mm isotropic resolution) from these participants were processed using the same structural MRI pipeline applied to the HCP dataset. Details regarding UK Biobank participant recruitment and data acquisition procedures are described in Miller et al., 2016[78]. All participants provided informed consent.*"

Methods (Lines 816-841): "***Cross-validation of anatomical sex differences and sex prediction in UK Biobank dataset***

*In two sensitivity analyses, we assessed the spatial reproducibility of sex differences in brain anatomy using an independent UK Biobank dataset and evaluated whether sex prediction performance in the HCP dataset was affected when the masks of significant sex differences in brain anatomy used to define training data were derived from the UK Biobank dataset (as opposed to an HCP dataset mask as in our primary analyses).*

*Structural MRI data from the UK Biobank sample were preprocessed using the same FreeSurfer pipeline applied to the HCP dataset. Two participants were excluded due to*

*poor surface reconstruction quality (Euler number < −217), resulting in a final sample of 528 males (49.0[1.0] yrs) and 636 females (48.9[1.1] yrs). Using the same Model 4, we identified significant regional sex differences (q < 0.05) in gray matter volume, surface area, and cortical thickness beyond global measures in the UK Biobank sample (**Supplementary Fig. 12a**).*

*First, to quantify the spatial correspondence of sex differences between the HCP and UK Biobank datasets, we computed spatial correlations of effect sizes (Cohen's d) across the 360 regions defined by the HCP parcellation. Statistical significance of this spatial correlation was assessed using spin tests with 10k spatial permutations (**Supplementary Fig. 12b**).*

*Second, to address potential inflation of sex prediction accuracy resulting from using the same dataset to define sex differences and perform prediction, we repeated the machine-learning analyses described above using masks of significant sex differences derived from the UK Biobank dataset (**Supplementary Fig. 12c**), rather than from the HCP dataset (original results shown in **Supplementary Fig. 10c**). We then quantified changes in prediction accuracy between these two conditions (**Supplementary Fig. 12c**). We also compared model outputs by computing Pearson correlations of sex typicality scores (STS) across participants between the primary model based on a sex difference mask defined in the HCP and the alternative model where this mask was defined in the UK Biobank (**Supplementary Fig. 12c**)."*

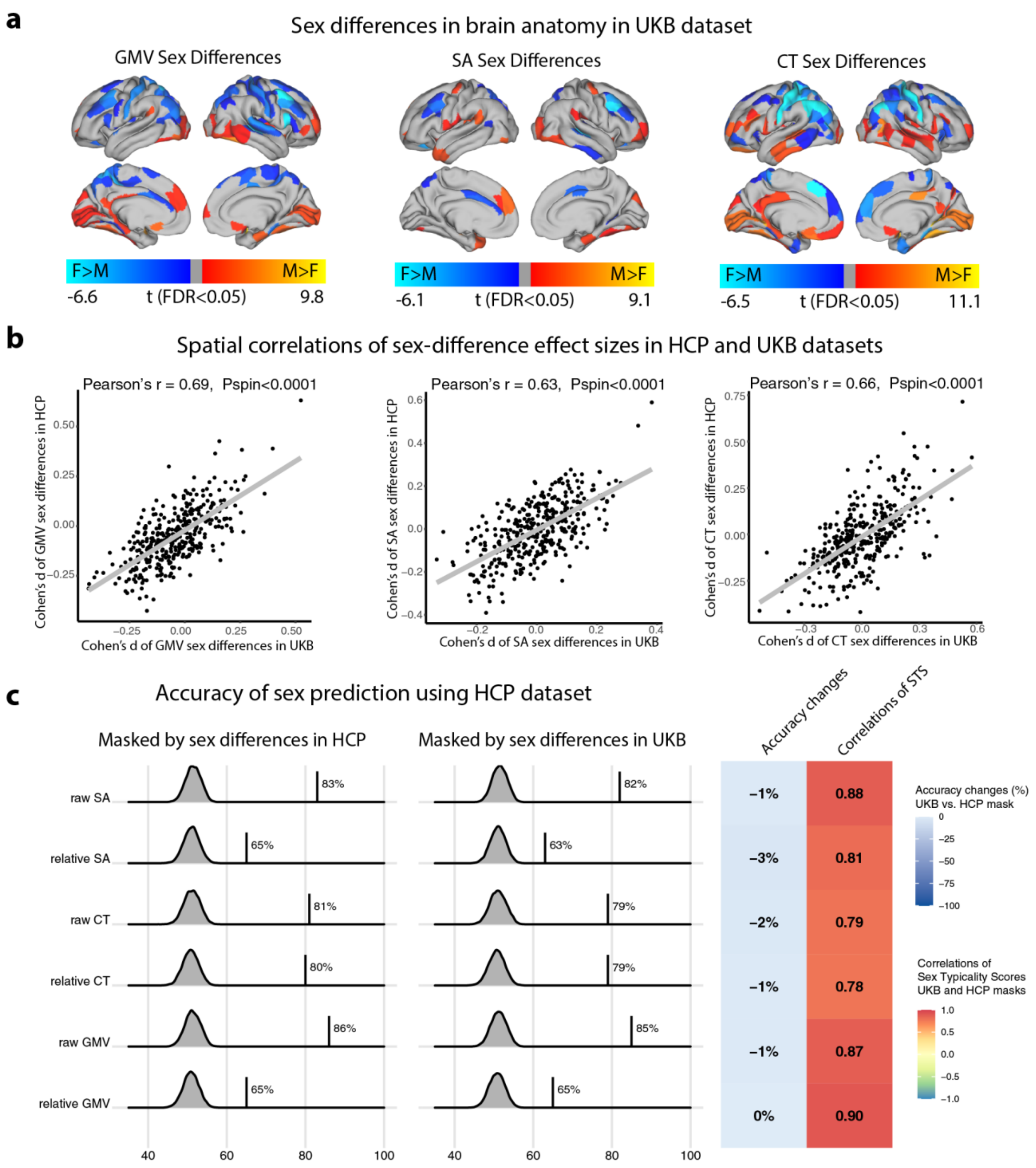


***Supplementary Fig. 12: Cross-validation of sex differences and sex prediction performance in brain anatomy using an independent UK Biobank dataset.***

*(**a**) We repeated the structural MRI analyses applied to the primary HCP dataset in an independent UK Biobank (UKB) sample and identified statistically significant sex differences in regional gray matter volume (GMV), surface area (SA), and cortical thickness (CT). Color maps show t values for regional sex differences after controlling for corresponding global measures (total GMV, total SA, and mean CT) and correcting for*

*multiple comparisons across brain regions (FDR q < 0.05). (**b**) Scatter plots showing strong spatial correspondence between structural sex differences derived from the UKB (x-axis) and HCP (y-axis) datasets. Each point represents Cohen's d for one of 360 brain regions defined by the HCP parcellation. Effect sizes were highly correlated (r > 0.63) and significantly exceeded chance levels based on 10,000 spin permutations (Pspin<0.0001). (**c**) Effects of using different datasets to define the mask of significant sex differences for sex prediction. In the left column, we reproduced the original sex prediction accuracy for each anatomical feature set (bars; see **Supplementary Fig. 10c**), where the HCP dataset was used for both mask definition and prediction, relative to a null distribution generated from 10,000 sex-label permutations. In the right column, masks of significant sex differences derived from the UKB dataset (panel **a**) were used as model inputs, and sex prediction was performed in the HCP dataset. The first color bar indicates changes in prediction accuracy between the two approaches, showing only a modest reduction (<3%) when using the independent UKB dataset for masking. The second color bar shows high correlations (r > 0.78) between sex typicality scores (STS) across participants obtained from the two modeling approaches, indicating that shared interindividual information was used for sex prediction in both cases.*

**Comment 3**

For the discussion I am missing a bit the interpretation of the why? If these levels are not really connected, why could this be the case? What are things that can or should be investigate to explain what is going on? Are similar observations made in other domains/species?

**Response 3**

We thank the reviewer for raising this important point. We have now added a new Discussion paragraph that speaks to the interesting questions they pose.

Discussion (Lines 471-498): "*Our overall finding of broad orthogonality between sex differences in brain activation, brain anatomy and behavior is striking, and could reflect several possible scenarios that are not mutually exclusive. One theoretical possibility is that sex differences in the constructs we consider here are in fact inter-related, but that this is obscured by error in our current measurement tools. While there remain active concerns about the reliability and validity of measures from functional neuroimaging[75], we note that sex-differences maps for most studied neuroimaging phenotypes showed high split-sample spatial reproducibility, and that reproducible correlations were evident between interindividual variation in brain activation and behavior within each sex. Nevertheless, it will be important to revisit this work as measurement accuracy and reliability improve. Another possibility is that — despite the unprecedented breadth of HCP fMRI task and behavioral data — there are some as-yet untested combinations of task paradigms and behaviors that could elicit prominent sex differences in the relationship between brain activation and behavior. Future tests of this idea could perhaps focus on sex-biased behaviors that are more pronounced[76,76] and conserved across species (e.g. suckling, physical aggression) and the subcortical circuits that support these behaviors[78].*

*Moreover, task-based fMRI represents only one of many approaches to measuring brain function, and cortical morphology captures only a subset of anatomical variation. While these measures account for a substantial proportion of the human neuroimaging literature, other functional modalities (e.g., resting-state fMRI, Electroencephalography, or Magnetoencephalography) and structural measures (e.g., diffusion-weighted imaging, volume-based morphometry, or T1/T2 ratio) may show stronger concordance with behavior and greater sensitivity to sex modulation effects than those examined here. It may also be that sex differences in the neuroimaging and behavioral features examined herein show stronger inter-relationships during dynamic windows of sex-biased brain development[17,79,80] and ageing[81]. It will be important to test these hypotheses in future work as necessary multi-task and multimodal datasets in these lifespan stages grow. There is also the more far-reaching possibility that in vivo neuroimaging features are fundamentally constrained in the proportion of interindividual behavioral variation that they can explain, which consequently limits their ability to detect group differences in brain-behavior correlations[66].”*

**Comment 4**
Did the authors take into account the reliability of the variables when investigating sex difference? Could it be explained by low reliability, high variance in one sex and not the other for example?

**Response 4**
We thank the reviewer for pointing this out and have now fully discussed this as a potential explanation for the absence of significant sex-modulation effects. This discussion has been included in the section addressing Response 3 above and is reproduced below for convenience.

Discussion (Lines 473-479): "*One theoretical possibility is that sex differences in the constructs we consider here are in fact inter-related, but that this is obscured by error in our current measurement tools. While there remain active concerns about the reliability and validity of measures from functional neuroimaging[75], we note that sex-differences maps for most studied neuroimaging phenotypes showed high split-sample spatial reproducibility, and that reproducible correlations were evident between interindividual variation in brain activation and behavior within each sex. Nevertheless, it will be important to revisit this work as measurement accuracy and reliability improve.”*

**Comment 5**
Why are the GMV findings in this study different than those published with the same dataset / authors in PNAS?

**Response 5**

We thank the reviewer for raising this important point. The GMV sex difference maps in this paper are similar to — but not identical with — those reported in the same sample in an earlier paper which used a different image processing tool (Liu et al, PNAS, 2020). We have now added more information to the Methods section of our manuscript to underline the differences between surface- and volume-based measures of cortical volume — which are likely to account for areas of mismatch between GMV sex difference maps and underline the non-equivalence between different measures of regional GMV. We reproduce this new text below.

Methods (Lines 595-605): "*Notably, the three morphometric measures examined here, cortical volume, surface area, and cortical thickness, extend the assessment of sex differences in cortical anatomy beyond the gray matter volume analyzed in our previous study*[50]*. Importantly, in the current work, cortical volume was derived as the product of cortical thickness and surface area using a surface-based FreeSurfer pipeline, representing the volume of the cortical sheet at each vertex while accounting for cortical folding. This measure is therefore not equivalent to the voxel-based gray matter volume used previously*[50]*, which was derived from volume-based morphometry and reflects the fraction of gray matter within each voxel in three-dimensional space without explicit consideration of cortical geometry. These methodological differences and associated non-equivalence of regional GMV differences from surface- vs. volume-based morphometry mean that the two methods cannot be assumed to generate identical group difference maps*[88]*.*"
